# Frequent birth-and-death events throughout perforin-1 evolution

**DOI:** 10.1186/s12862-020-01698-1

**Published:** 2020-10-19

**Authors:** Miguel Araujo-Voces, Víctor Quesada

**Affiliations:** 1 Departamento de Bioquímica y Biología Molecular - IUOPA, Universidad de Oviedo, C/ Fernando Bongera S/N, Oviedo, 33006 Spain; 2 Centro de Investigación Biomédica en Red de Cáncer (CIBERONC), Madrid, Spain

**Keywords:** Perforin-1, Assisted annotation, Immune, Birth-and-death, Tandem duplication

## Abstract

**Background:**

Through its ability to open pores in cell membranes, perforin-1 plays a key role in the immune system. Consistent with this role, the gene encoding perforin shows hallmarks of complex evolutionary events, including amplification and pseudogenization, in multiple species. A large proportion of these events occurred in phyla for which scarce genomic data were available. However, recent large-scale genomics projects have added a wealth of information on those phyla. Using this input, we annotated perforin-1 homologs in more than eighty species including mammals, reptiles, birds, amphibians and fishes.

**Results:**

We have annotated more than 400 perforin genes in all groups studied. Most mammalian species only have one perforin locus, which may contain a related pseudogene. However, we found four independent small expansions in unrelated members of this class. We could reconstruct the full-length coding sequences of only a few avian perforin genes, although we found incomplete and truncated forms of these gene in other birds. In the rest of reptilia, perforin-like genes can be found in at least three different loci containing up to twelve copies. Notably, mammals, non-avian reptiles, amphibians, and possibly teleosts share at least one perforin-1 locus as assessed by flanking genes. Finally, fish genomes contain multiple perforin loci with varying copy numbers and diverse exon/intron patterns. We have also found evidence for shorter genes with high similarity to the C2 domain of perforin in several teleosts. A preliminary analysis suggests that these genes arose at least twice during evolution from perforin-1 homologs.

**Conclusions:**

The assisted annotation of new genomic assemblies shows complex patterns of birth-and-death events in the evolution of perforin. These events include duplication/pseudogenization in mammals, multiple amplifications and losses in reptiles and fishes and at least one case of partial duplication with a novel start codon in fishes.

## Background

Pore formation is an important step in the immune response in at least three settings: against extracellular bacteria, against virus-infected, cancer or senescent cells and against intracellular bacteria [1]. Regarding the second setting, killing damaged or malfunctioning endogenous cells (immunosurveillance) not only protects the organism against external viruses, but also fights tumor development and premature aging. In fact, this activity is linked to hallmarks of cancer (avoid immune destructuion and tumor-promoting inflammation) [2] and aging (altered intercellular communication) [3].

In vertebrates, pore formation during immunosurveillance is carried out by the product of the perforin-1 gene (*PRF1*). According to two prevalent models, pores open by PRF1, either in the plasma membrane or after endocytosis in the endosomal membrane, allow pro-apoptotic granzymes to enter the cytosol of target cells [4]. Consistent with this role, it has been recently shown that mice deficient in perforin-1 (*Prf1*^-/-^) display increased tumor burden in a mammary tumor model [5]. In a separate work, *Prf1*^-/-^ mice were also shown to suffer accelerated aging through accumulation of senescent cells [6].

Perforin-1 belongs to the *membrane attack complex / perforin family* (MACPF), which includes pore-forming and development-related proteins [7, 8]. All MACPF members contain a signal peptide and an MACPF domain, although different sub-families display widely diverse combinations of ancillary domains. Thus, PRF1 orthologs consist of a signal peptide, an MACPF domain, an EGF-like domain and a C2-like domain. Upon Ca^2+^-dependent binding of the C2 domain to a membrane of the target cell, perforin monomers assemble into pores in a fast process that allows the delivery of granzymes before the exocytic repair response reseals that membrane [9].

Given the involvement of PRF1 in immunity, cancer and aging, its coding gene is expected to be under selective pressure. In this regard, previous work have shown frequent and complex events of gene gain and loss throughout evolution [10, 11]. While annotating the genomes of giant tortoises [12], we observed multiple new gene amplifications and deletions in reptiles. Some of these events were hard to detect by automatic annotation, and they were only evident after applying our expertise-assisted annotation algorithm. We therefore decided to apply this annotation pipeline to novel and improved publicly available genome assemblies to improve our understanding of *PRF1* evolution.

## Results

**Fig. 1:**
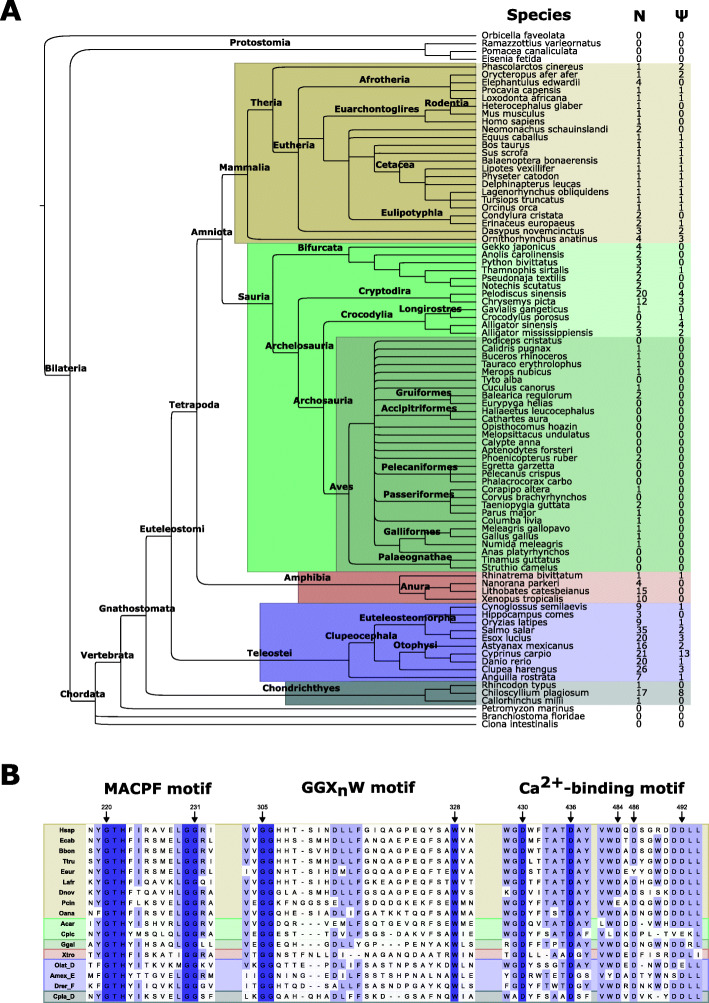
Vertebrate perforin-1 genes. **a** phylogenetic relationships between annotated species. For each species, the number of putatively functional (N) and pseudogenized (*Ψ*) genes is shown. **b** representative alignment of annotated perforin-1 amino acid sequences. Key residues in each motif are highlighted with an arrow. Numbers correspond to the human protein. Hsap, *Homo sapiens*; Ecab, *Equus caballus*; Bbon, *Balaenoptera bonaerensis*; Ttru, *Tursiops truncatus*; Eeur, *Erinaceus europaeus*; Lafr, *Loxodonta africana*, Dnov, *Dasypus novemcinctus*; Pcin, *Phascolarctos cinereus*; Oana, *Ornithorhynchus anatinus*; Acar, *Anolis carolinensis*; Cpic, *Chrysemys picta*; Ggal, *Gallus gallus*; Xtro, *Xenopus tropicalis*; Olat, *Oryzias latipes*; Amex, *Astyanax mexicanus*; Drer, *Danio rerio*; Cpla, *Chiloscyllium plagiosum*

We studied genome assemblies of 87 species, including mammals, reptiles, birds, amphibians and fishes, as well as several outgroups (Fig. 1, Additional file 1). By using assisted annotation and manual curation, we annotated 405 sequences, including 73 classified as pseudogenes due to the presence of early stop codons (Additional files 2-7). To identify probably conserved loci, we also searched for flanking genes from the sets of automatically annotated genes (Additional file 1). An alignment of sequences from every group shows the conservation of functional residues, including the MACPF signature motif, the MACPF *GGX*_*n*_*W* motif [7] and the C2 Ca^2+^-binding site (Fig. 1). The conservation of these motives suggests that the annotated genes produce proteins that share biochemical traits and belong to the same family.

### Mammals

**Fig. 2:**
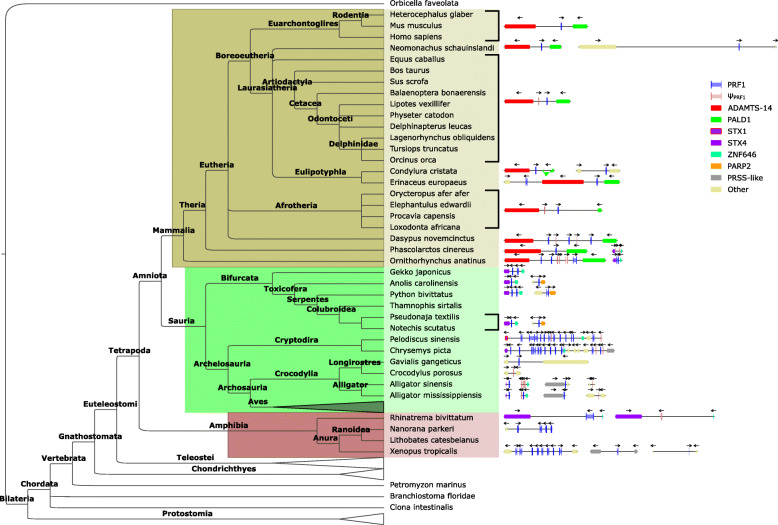
Evolution of mammalian, non-avian-reptile and amphibian perforin-1 loci. Left, phylogenetic relationship between selected species. Right, main *PRF1* loci. Perforin-1 is depicted to scale with intron/exon junctions. Other genes are shown as blocks and may be cropped for ease of presentation

Most mammalian genomes (Fig. 1, brown box) contain one functional *PRF1* gene (Fig. 2). In the case of the Euarchontoglires analyzed (humans, mice and naked mole rats), we have not found any other *PRF1*-related sequence. As described earlier in [10], this gene sits between *ADAMTS14* and *PALD1* (also known as *KIAA1274*). In almost all Afrotheria and Laurasiatheria, including Cetacea, we also found a pseudogene in the same locus, closer to *ADAMTS14*. The only exceptions come from the star-nosed mole (*Condylura cristata*), the hedgehog (*Erinaceus europaeus*) and the hawaiian monk seal (*Neomonachus schauinslandi*). The genomes of these species show two functional *PRF1* genes, one at the *ADAMTS14*-*PALD1* locus and one at a different locus. The flanking genes in the additional locus are not related in these species, which suggests that these novel genes appeared independently. A phylogenetic analysis of the protein sequences supports this hypothesis (Additional file 8).

This simple arrangement is more complex in other mammals. Thus, the genome of the armadillo (*Dasypus novemcinctus*) shows three *PRF1* genes and two related pseudogenes at the same locus as humans (Fig. 2). According to the phylogenetic analysis, these genes arose in armadillo-specific duplication events (Additional file 8). The arrangement of perforins is particularly interesting in the two non-Eutherian mammals that we have analyzed. Thus, both the genomes of koalas (*Phascolarctos cinereus*) and platypus (*Ornithorhyncus anatinus*) show one additional *PRF1* locus, between syntaxin-4 (*STX4*) and *ZNF668* (Fig. 2). This extra perforin-1 in koalas was classified as a pseudogene.

These results are compatible with the existence of two ancestral mammalian *PRF1* loci, *ADAMTS14*-*PALD1* and *STX4*-*ZNF646*, the latter arising from some common ancestor to, at least, all mammals. Then, a common ancestor to all Eutheria (Fig. 2, inside the brown box) lost the *STX4*-*ZNF646* locus (koalas seem to have lost the corresponding gene independently by pseudogenization). During mammalian evolution, different lineage-specific gene duplications, pseudogenizations and losses have increased the number of *PRF1* genes independently. The only likely common events would be a duplication/pseudogenization event in Boroeutheria at the *ADAMTS14*-*PALD1* locus and the loss of the resulting pseudogene in Euarchontoglires.

### Sauria

We also annotated perforin genes in the genomes of 43 species of sauria (Fig. 1, green boxes). The results showed widely different evolutionary patterns, as suggested by previous studies [10, 12]. Fortunately, the improved genomic assemblies for many of these species have allowed a more detailed view of perforin-1 evolution.

#### Non-avian sauria

In all Bifurcata, Cryptodira and Crocodylia (Fig. 1, light green box), we have found evidence for the *STX4*-*ZNF646* locus (Fig. 2). The only exception was in the two longirostres (Crocodylia) analyzed, whose genomes contain unique loci. Notably, the sole *PRF1* locus in *Crocodylus porosus* contains a pseudogene, which would make it the only non-avian Gnastomata species without any functional perforin-1 in its genome. However, absence of a gene in a genome assembly does not prove absence of said gene in the genome of the species. Several members of clade Toxicofera present a specific locus characterized by a flanking *PARP2* gene. Finally, turtles display multiple copies of *PRF1* within the *STX4*-*ZNF646* locus, consistent with specific events of tandem gene amplification in this group.

#### Birds

**Fig. 3:**
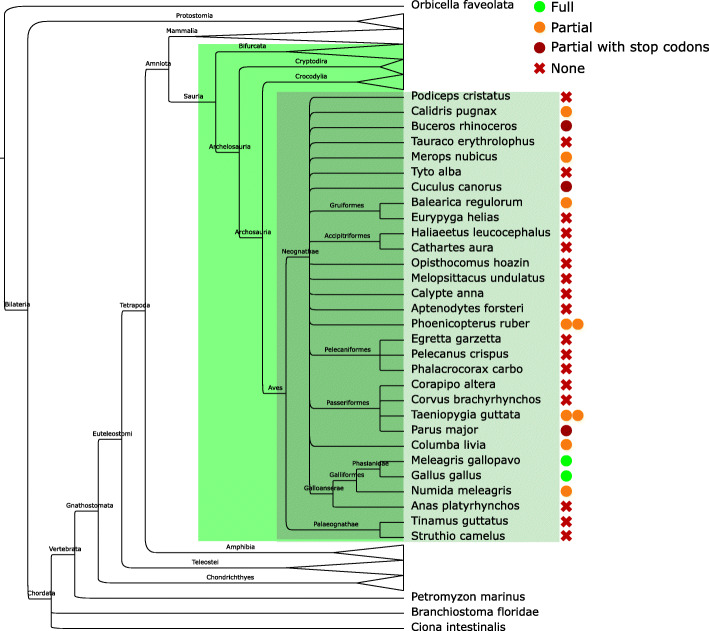
Avian perforin-1 genes. For each bird species, annotated perforin-1 sequences are depicted according to the status and completeness of the prediction

Taking advantage of recent efforts aimed at the sequencing and assembly of avian genomes [13], we have annotated perforin-1 in 30 birds (Fig. 1, dark green box). At this time we were able to annotate full-length, putatively functional avian perforin-1 genes in chicken (*Gallus gallus*) and turkey (*Meleagris gallopavo*). We also found partial *PRF1*-like sequences in other seven bird genomes (Fig. 3). These include two putative copies of this gene in zebra finch (*Taeniopygia guttata*) and in *Phoenicopterus ruber*.

We did not find any evidence of functional perforin-1 genes in most avian genomes. However, we observed that most of the partial perforin-like sequences are located in small contigs and share little similarity, which suggests that the avian *PRF1* locus may be hard to rebuild. Therefore, we looked for evidence of expression of these putative perforins. After compiling a set of publicly available RNA-Seq experiments on zebra finches (Additional file 9), we looked for reads displaying similarity to the partial sequences we had annotated. With this approach, we found multiple reads supporting the expression of the annotated sequences. Moreover, partially overlapping reads allowed us to infer parts of the *PRF1*-like gene which showed low similarity to other perforins. This result supports the hypothesis that avian perforin-1 loci are hard to identify, and hence other birds may have versions of the gene that cannot be annotated by similarity.

### Amphibians

To better characterize the perforin-1 loci in Tetrapoda, we annotated four amphibian genome assemblies (Fig. 1, pink box). These amphibians included one ranoidea (*Rhinatrema bivittatum*), in whose genome we found one *STX4*-*ZNF646* locus containing a putatively functional *PRF1* gene and a pseudogene. However, we did not find evidence of this locus in the genomes of three Anura analyzed. By contrast, in these species we found perforin-1 loci containing multiple copies of *PRF1* and with flanking genes not located around any other known *PRF1* locus (Additional file 5). These results are consistent an *STX4*-*ZNF646* perforin-1 locus in an ancestor to all Tetrapoda which was lost in Anura and not in Ranoidea.

### Fishes

In addition to providing a link to ancient Euteleostomi, teleostei display striking genomic versatility [14] and live under unique environmental challenges that make them an interesting model for immune system studies [15]. Therefore, we annotated perforin-1 homologs in the genome assemblies of 10 fish species (Fig. 1, light blue box). In this analysis, we found evidence of numerous duplication events, leading to a total of 125 fish genes and 25 pseudogenes. Although a few shared loci can be identified, most of the annotated sequences belong to species-specific loci, according to the nature of the flanking genes (Additional file 6). Additionally, we found a unique sub-family of smaller *PRF1*-like genes whose coding sequences only contain a secretion peptide and a C2-like domain. We have tentatively called this sub-family *c2PRF1*.

**Fig. 4:**
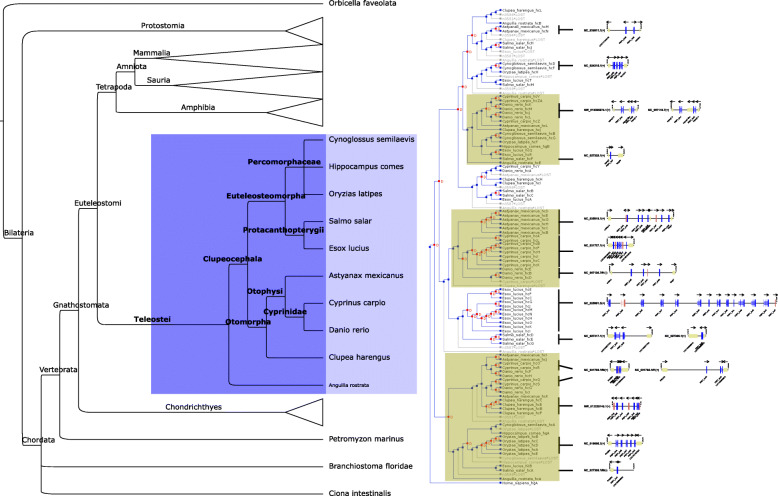
Birth-and-death in fish perforin-1 evolution. Left, phylogenetic relationship between selected teleost species. Middle, Birth-and death events according to a model of maximum parsimony. Duplications are highlighted in *red*, and predicted gene losses are depicted in *grey*. Branches showing conserved loci are highlighted in *brown*. Right, selected *PRF1* loci corresponding to branches in the birth-and-death tree

To delve into these evolutionary events, we attempted to infer the phylogenetic relationships between the annotated sequences and then interpret the resulting tree according to the accepted fish taxonomical relationships. Thus, we aligned all the inferred fish perforin-1 protein sequences that did not lack significant stretches. We added the sequence of human PRF1 for later use as an outgroup. Then, we searched for trees compatible with the alignment by both Bayesian inference (MrBayes) and maximum parsimony (TNT). Given the complexity of the result, we used Notung to infer the order of duplications and losses. As shown in Fig. 4, this method predicted multiple duplications throughout Teleostei evolution. These include several independent species-specific tandem amplifications, particularly in *Astyanax mexicanus*, *Cyprinus carpio*, *Esox lucius* and *Oryzias latipes*. The loci where these genes are located seem to be extremely diverse, with quasi-orthologous copies surrounded by unrelated genes. Nevertheless, we found at least three loci with significant, albeit imperfect, conservation. These regions are characterized by *MPEG1*-like, *S100*-like and *PAQR4*-like genes (Fig. 4).

One of the possible mechanisms for bursts of gene duplications with locus diversification is transposon activation [16]. In an exploratory analysis, we looked for known transposon-like sequences inside *PRF1* contigs from *O. latipes* and *Danio rerio* using FishTEDB. This search located numerous LTR, TIR, LINE and DNA/DNA-type transposons (Additional file 10). Most of these sequences are located in intergenic regions, close to *PRF1*-like genes. However, we also found several putative transposons in intronic regions, both inside perforins and flanking genes. In the case of *O. latipesPRF1f*, an LTR-like element is located right upstream of one of the genes, occupying the stretch corresponding to the promoter.

**Fig. 5:**
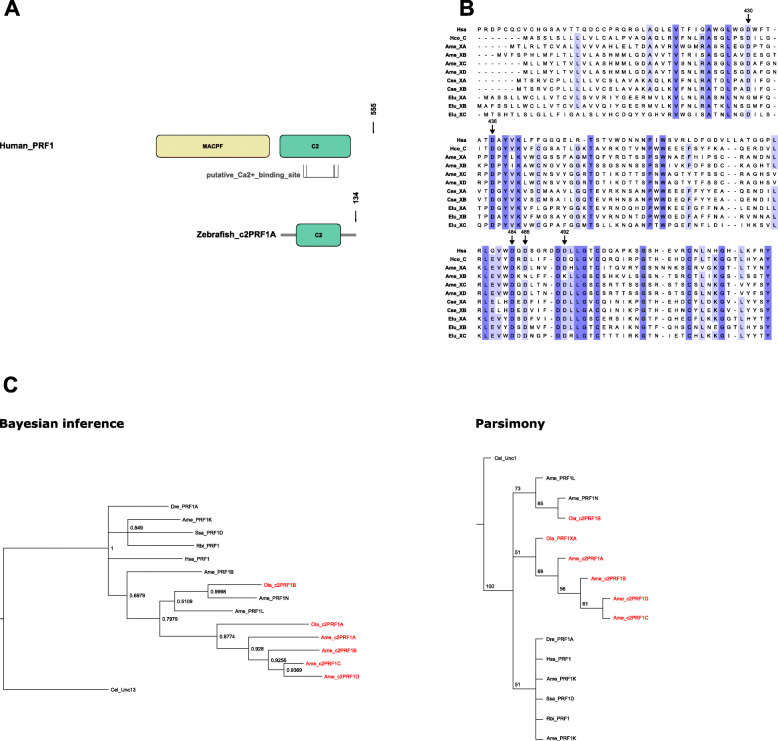
Structure and origin of fish *c2PRF1* genes. **a** domain composition of full-length perforin-1 (human) and c2PRF1 proteins (zebrafish). **b** amino acid alignment of c2PRF1 proteins (_X) with human perforin-1. Arrows highlight residues probably involved in Ca^2+^ binding. Hsa, *Homo sapiens*; Hco, *Hippocampus comes*; Ame, *Astyanax mexicanus*; Cse, *Cynoglossus semilaevis*; Elu, *Esox lucius*. **c** Bayesian inference (left) and maximum-parsimony (right) phylogenetic trees from teleost c2PRF1 (*red*) and PRF1 (*black*) protein sequences. Node numbers depict posterior probability (left) or robustness as assessed by bootstrapping (right). Dre, *Danio rerio*; Ame, *Astyanax mexicanus*; Ssa, *Salmo salar*; Rbi, *Rhinatrema bivittatum*; Ola, *Oryzias latipes* Cel, *Caenorhabditis elegans*

We also investigated the origin of *c2PRF1* genes. First, we examined the sequence of their products and confirmed that all of them are expected to possess secretion peptides according to a prediction program (Additional file 11). Then, we aligned the rest of their sequences, except for those with large missing stretches. Since we did not know a priori whether *c2PRF1* genes arose before or after perforin-1 genes, we added the C2-like domain of Unc-13 from *Caenorhabditis elegans* as an outgroup. Unfortunately, the result of this analysis cannot reliably predict the order of appearance of these two sub-families, even if it suggests that *c2PRF1* has a more recent origin (Fig. 5). Surprisingly, both inference methods robustly suggest that *c2PRF1* genes have two independent origins, most of them early during Teleost evolution and only one (*c2PRF1Xb* from *O. latipes*) much later, with a closer common ancestor to other full-length *PRF1*-like genes.

Taken together, these results suggest a complex scenario where fish *PRF1*-like genes were frequently duplicated, with bursts of tandem amplifications possibly driven by transposon jumping. Perhaps as a result of these jumps, a second family of secreted genes, whose products only contain the C2 domain, arose from two separate events.

### Cartilaginous fishes

To more precisely establish the origin of perforin-1, we annotated *PRF1* homologs in three chodrichtyes genome assemblies (Fig. 1, dark blue box). We found functional copies of this gene in all three species, which suggests that *PRF1* arose early during Gnastomata evolution. Notably, all the annotated functional copies show a single exon with no introns. While two of the three species studied display one functional *PRF1* gene, *Chiloscyllium plagiosum* contains 17 putative functional copies of this gene and 8 pseudogenes. The whale shark (*Rhincodon typus*) presents one partial gene and one full-length *PRF1* with in-frame premature stop codons. However, using RNA-Seq databases, we found reads consistent with the premature stop codons being due to assembly artifacts (Additional File 12).

## Discussion

In this work, we have used similarity- and expertise-based algorithms to annotate *PRF1* homologs in numerous species spanning most major groups belonging to Gnastomata. While much slower than automatic annotation, this approach has two important advantages for this project. First, it allows the annotation of divergent and incomplete genomic stretches if the researcher has access to other sources of information. Second, the researcher has first-hand knowledge of these problems as soon as they are detected. Thus, we were able to find *PRF1*-like sequences that had not been previously annotated in several genomic assemblies.

The results of these annotations show remarkable variations in the number and context of *PRF1* homologs in different taxonomic groups. Mammals seem to have reached a stable configuration with one conserved locus (*ADAMTS14*-*PALD1*) that is present in all the species analyzed. In fact, the most parsimonious hypothesis sets the origin of this locus at the last common ancestor of Mammalia. Our results suggest that this ancestral species had at least one additional locus, *STX4*-*ZNF646*, which is also present in reptiles, amphibians and perhaps teleosts. Therefore, this locus may have been inherited from a common ancestor to Euteleostomi (Fig. 1).

Thus, we have found *STX4*-*ZNF646* loci in most reptiles. Turtles seem to have experienced tandem duplications of *PRF1* at this common locus, as previously shown [12]. Our results in birds, with partial coding sequences confirmed and extended by RNA-Seq reads, are compatible with the existence of *PRF1*, as previously suggested [17]. Notably, while the turtle *Chrysemys picta* shows a standard *STX4*-*ZNF646* locus, the related *Pelodiscus sinensis* has an inverted copy of an *STX1*-like gene instead of *STX4*. In fact, the automatic annotation does not identify any *STX4*-like gene in this assembly. In multiple species, including *C. picta*, *STX1B* and *STX4* are located in tandem and tail-to-tail (Additional file 13). Therefore, a deletion of the *STX4* locus in an ancestor of *P. sinensis* is enough to explain this result.

In this regard, we have found two teleost loci, one in *Salmo salar* and one in *D. rerio*, with an *STX1*-like flanking gene (also discussed in [10]). However, in *D. rerio* we have found a clear ortholog of *STX4* with no perforin-like flanking genes (Additional file 10). In light of these results, it is possible that these teleost perforins stem from an existing locus from an ancestor to all Euteleostomi. In this scenario, most teleosts have lost this locus, and in other fishes *STX4* has been lost or has been transposed. The preliminary state of genome assemblies of Chondrichtyes (cartilaginous fishes) precluded a rigorous study of flanking genes in *PRF1* loci. However, we found that all functional *PRF1* genes in this group contain one exon, which suggests that they arose after retrotransposition events and therefore their loci are unrelated to those of other species. Further work on the evolution of syntaxins in these species will shed light on the relationships between these *PRF1* loci.

Aside from syntaxin-related loci, fishes display the widest variation in number and context of *PRF1*-like genes. A large proportion of amplifications seems to be species-specific, in an example of evolution through a birth-and-death process [18]. The pattern of duplication bursts with changes of location is reminiscent of amplification by transient activation of transposons. Consistent with this hypothesis, we found multiple transposon-like sequences at perforin-1 loci, both in intergenic and intronic regions. In *O. latipes*, we have found a gene (*PRF1_hcF*) with an LTR-like sequence where its promoter should be. This suggests that both sequences might share the same promoter. This situation has been described with DNA transposons, and is hypothesized to be a mechanism that improves the chances of successful fixation of transposons [19].

It has been hypothesized that transposon activation may lead to an adaptive advantage in rapidly shifting environments, such as those faced by an invasive species [20]. These putative activation episodes may mediate genomic instability and duplication of nearby genes through incorrect DNA excision or mismatched homologous recombination. Fixation of these duplicated copies will then depend on genetic drift or positive selection. Therefore, these results suggest that some teleost species may have experienced bursts of DNA transposon-mediated *PRF1* duplication which were not deleterious. It is important to notice that at least one study has ascertained the cytolysis-promoting activity of a *D. rerio* perforin-1 [21]. Further studies will assess the putative positive selection acting on these amplified *PRF1* genes, and whether the presence of a larger number of copies may confer adaptive advantages.

This apparent selective pressure acting on teleost perforin-1 may also have led to evolutionary invention of new genes. Thus, we have found a group of *PRF1*-related genes, tentatively called *c2PRF1*, which consist of a signal peptide followed by a C2-like domain. Since it has been proposed that *PRF1* is the result of a fusion between an *MPEG1*-related gene and a C2-like domain [10], this family might stem from the ancestral gene that gave rise to this second domain. However, the similarity between *c2PRF1* and the C2 domain of fish *PRF1* suggests that their common origin is more recent. Indeed, according to a preliminary phylogenetic analysis, these genes arose in two separate and independent events. The first event seems to have happened early during teleost evolution, although the precise forking sequence cannot be reliably assessed. The second event gave rise to one *c2PRF1* in *O. latipes*, and was independently and reliably predicted by two methods. This suggests that the fixation of *c2PRF1* genes may be beneficial for teleosts, in a remarkable example of convergent evolution.

The model contending that *c2PRF1* genes stem from full-length *PRF1* genes by partial duplication poses intriguing questions. For instance, it is not clear how each signal peptide originated. While these peptides display high sequence variability [22], the constraints to their sequence make them highly unlikely to appear by chance alone. Therefore, if the original *c2PRF1* arose after a partial duplication close to a functional promoter and was expressed from a random start methionine, the resulting protein would likely be intracellular for many generations, until random mutation and selection fixated a signal peptide. A different model would have the starting insertion close to an existing signal peptide. This model would predict a much higher number of duplication events, as the likelihood of one event placing a DNA fragment close to a promoter followed by a signal peptide and in the same translational frame would be exceedingly low.

Another major question concerns the biological role of the product of *c2PRF1* genes. Since the C2 domain of perforin-1 is involved in binding to the cell membrane of target cells and in oligomerization, a tentative hypothesis would consider these novel proteins as adaptors that anchor other factors to pore-like structures in cell membranes. In this regard, we have found that residues involved in Ca^2+^ binding, a necessary step for this process, are conserved in most *c2PRF1* products. Further experiments with native or recombinant proteins will be needed to confirm and develop this hypothesis.

In a broader sense, the functional consequences of both the invention of *c2PRF1* and the genomic amplification of *PRF1* paralogs may be related to the complex evolution of MACPF/CDC genes in vertebrates. Thus, the MACPF domain is present in proteins that play disparate roles. Some of these proteins, like astrotactins and BRINPs, do not seem to be involved in pore formation [23]. These diverse roles reflect a highly versatile modular architecture, with acquisitions and losses of ancillary domains. Therefore, the emergence of *c2PRF1* may be a new example of this modularity, which would complement the roles of MACPF domain-containing proteins. By contrast, the structural conservation of *PRF1* paralogs suggests that they all play a role in pore formation. In this scenario, additional copies of perforin-1 might be expressed or secreted in response to different stimuli, which would add another layer of complexity to the innate immune response in vertebrates like reptiles, fishes and amphibians. Recent reports have suggested that the innate immune response is regulated beyond the well-known generic and non-specific responses, which has led to the concept of trained immunity [24]. It will be interesting to study the regulation of *PRF1* expression in species with multiple paralogs and its putative relationship with the fine tuning of innate immunity.

In summary, the present study shows frequent and independent birth-and-death events in the evolution of *PRF1*. These events seem to have happened in bursts of duplications followed by deletions and pseudogenizations. In addition, teleosts seem to have acquired a novel family of related proteins through partial duplication of the last exon of *PRF1*. Taken together, these results point to an important role of perforin-1 throughout the evolution of Gnastomata, probably related to its function in the immune system. Future studies on the evolution of this gene may yield important data on how its activity is modulated in the context of changing environments, and how those changes affect the biology of cancer and aging.

## Conclusions

We have annotated 405 *PRF1* homologs in 87 species of mammals, reptiles, amphibians and fishes. We have found evidence for vertical inheritance of an ancestral perforin-1 locus with flanking syntaxin genes. This locus was lost in a common ancestor of most mammals, but can be found in marsupials and platypus. Numerous species and phylogenetic groups have undergone gains and losses of *PRF1* genes independently. These include turtles, Anura, multiple teleosts and cartilaginous fishes. Finally, we have found evidence for a novel related family of C2-containing proteins arising from teleost *PRF1* genes.

## Methods

### Gene annotation

To annotate perforin homologs, we used the iterative knowledge-based BATI algorithm [12]. Briefly, a set of protein sequences (human PRF1 or a more suitable ortholog) is aligned to the target reference genome with tblastn using *tbex*. Then, each set of tblastn hits corresponding to a target homolog is selected with *blastsniffer*. The exon/intron junctions are then established with *genetuner*. The results are fed into *bgmix* to check for novel paralogs of the annotated genes and the whole procedure is iterated until no more paralogs are detected. Every step is supervised by a researcher who also integrates other sources of information, such as publicly available RNA-Seq experiments. Only coding sequences were annotated. Loci were plotted with a custom script that integrates *gff* files resulting from BATI with other *gff* files with automatic annotations from the NCBI.

### Sequence characterization

To predict whether fish *c2PRF1* proteins contain secretion peptides, we used SignalP [25]. To locate putative transposon-like sequences in fish perforin-1 loci, we used the BLAST tool at FishTEDB [26]. Predictions on domain architecture and functional motives relied on the CD-BLAST tool at NCBI.

### Phylogenetic analyses

To infer the order of duplications and losses of perforin genes, we generated phylogenetic trees of specific taxonomic groups. First, we aligned the corresponding protein sequences, including one sequence belonging to a different group to use as an outgroup, with Clustal Omega v1.2.4 [27]. Then, we inspected the alignment with Mesquite v3.61 (http://www.mesquiteproject.org). We deleted sequences with large gaps and regions where the characters were too divergent at the start and end of the alignments. Given the uncertainties of annotating sequences in some of the assemblies, we converted some internal gaps into missing characters.

We then generated phylogenetic trees using MrBayes v3.2.6 [28]. We set the substitution model to Wag, invariant sites and a gamma distribution. We set two runs of at least 300,000 generations with trees sampled every 100 generations. The particular parameters for each run can be found in the Supplemental Data (see availability of data and material). The runs were evaluated with Tracer v1.7.1 [29] and the resulting trees were plotted with Figtree v1.4.4 (http://tree.bio.ed.ac.uk/software/figtree/).

To help in the interpretation of branches with low posterior probability, we also generated phylogenetic trees from the same alignments by maximum parsimony using TNT Willi Henning Society Edition v1.5 [30]. Alignments were exported from Mesquite in tnt format and fed into TNT with gaps treated as a character. First, we set the outgroup taxon. Then, we stored the tree tags and ran a resampling analysis. The type was traditional search with Bootstrap, standard (sample with replacement). The number of replicates was set to 100 and the output result was set to absolute frequencies. A cutoff bootstrap score of 50 was used to collapse groups. The resulting trees were exported as *emf* files.

For particularly complex birth-and-death scenarios, we used Notung v2.9.1.3 [31]. Species trees were obtained from Taxonomy Common Tree at the NCBI [32]. Since Notung does not accept any polytomy in the species tree, we manually added a node separating *Hippocampus comes* from a common ancestor to *C. semilaevis* and *O. latipes*.

## Supplementary information

**Additional file 1** Genome assemblies and automatic annotations used in this work

**Additional file 2** Perforin-1 loci in mammalian species. *PRF1* genes are depicted to scale with intron/exon boundaries (*blue boxes*). Pseudogenes are depicted in *pink*. Flanking genes may be cropped for ease of depiction.

**Additional file 3** Perforin-1 loci in non-avian reptiles. *PRF1* genes are depicted to scale with intron/exon boundaries (*blue boxes*). Pseudogenes are depicted in *pink*. Flanking genes may be cropped for ease of depiction.

**Additional file 4** Perforin-1 loci in birds. *PRF1* genes are depicted to scale with intron/exon boundaries (*blue boxes*). Pseudogenes are depicted in *pink*. Flanking genes may be cropped for ease of depiction.

**Additional file 5** Perforin-1 loci in amphibians. *PRF1* genes are depicted to scale with intron/exon boundaries (*blue boxes*). Pseudogenes are depicted in *pink*. Flanking genes may be cropped for ease of depiction.

**Additional file 6** Perforin-1 loci in teleosts. *PRF1* genes are depicted to scale with intron/exon boundaries (*blue boxes*). Pseudogenes are depicted in *pink*. Genes with names containing *hcX* belong to the *c2PRF1* family. Flanking genes may be cropped for ease of depiction.

**Additional file 7** Perforin-1 loci in cartilaginous fishes. *PRF1* genes are depicted to scale with intron/exon boundaries (*blue boxes*). Pseudogenes are depicted in *pink*. Flanking genes may be cropped for ease of depiction.

**Additional file 8** Phylogenetic relationships between mammalian *PRF1* genes. Bayesian inference (left) and maximum-parsimony (right) phylogenetic trees from mammalian perforin-1 sequences. Names in *red* belong to genes in loci different from *ADAMTS14*-*PALD1*. Oan, *Ornithorhynchus anatinus*; Pci, *Phascolarctos cinereus*; Mmu, *Mus musculus*; Hgl, *Heterocephalus glaber*; Dno, *Dasypus novemcinctus*; Laf, *Loxodonta africana*; Pca, *Procavia capensis*; Eeu, *Erinaceus europaeus*; Hsa, *Homo sapiens*; Cch, *Condylura cristata*; Nsc, *Neomonachus schauinslandi*; Eca, *Equus caballus*; Ssc, *Sus scrofa*; Bta, *Bos taurus*; Pca, *Physeter catodon*; Bbo, *Balaenoptera bonaerensis*; Dle, *Delphinapterus leucas*; Oor, *Orcinus orca*; Cpl, *Chiloscyllium plagiosum*.

**Additional file 9** Sequence read archive entries used to study bird *PRF1* expression

**Additional file 10** Transposon-like sequences in *PRF1* loci of *Danio rerio* and *Oryzias latipes*. *PRF1* genes are depicted to scale with intron/exon boundaries (*blue boxes*). Pseudogenes are depicted in *pink*. Transposon-like sequences are shown as *black boxes*.

**Additional file 11** SignalP prediction of secretion peptides in *c2PRF1* products

**Additional file 12** RNA-Seq reads aligned to *Rhincodon typusPRF1*. *Top*, Alignment of RNA-Seq reads from whale-shark blood cells to a genomic region belonging to *PRF1* and showing premature stop codons. The genomic sequence is shown on a *green* background. RNA-Seq reads are depicted on a *blue* (high-quality base) or *red* (low-quality base) background. *Dots* and *commas* represent bases equal to those of the corresponding genomic location. *Dashes* represent deletions of the reads as compared to the genomic sequence. *Bottom*, translation of the genomic sequence (*green background*) and two of the RNA-Seq reads from the top panel.

**Additional file 13** *STX4*-*ZNF646* locus in turtles. *PRF1* genes are depicted to scale with intron/exon boundaries (*blue boxes*). Pseudogenes are depicted in *pink*. Flanking genes may be cropped for ease of depiction.

## Data Availability

Scripts, gene annotations and gene alignments are publicly available in Github (https://github.com/vqf/PRF1).

## Abbreviations

MACPF: Membrane attack complex / perforin family
CDC: Cholesterol-dependent cytolysin
LTR: Long terminal repeats
TIR: Inverted tandem repeats
LINE: Long interspersed nuclear element.
