# Supplementary material for: Frequent birth-and-death events throughout perforin-1 evolution
Source: BMC Evol Biol. 2020 Oct 19;20:135. doi: 10.1186/s12862-020-01698-1 (PMC7574235; doi:10.1186/s12862-020-01698-1)
Supplement: Supplementary file 1 — Additional file 1 Genome assemblies and automatic annotations used in this work [file 12862_2020_1698_MOESM1_ESM.pdf]

Annotation project	Species	Assembly	Annotation
allMis4	*Alligator mississippiensis*	GCA_000281125.4	GCF_000281125.3_ASM28112v4_genomic.gff.gz
allSin1	*Alligator sinensis*	GCA_000455745.1	GCF_000455745.1_ASM45574v1_genomic.gff.gz
anaPla1.5	*Anas platyrhynchos*	GCA_003850225.1	
andRos1	*Anguilla rostrata*	GCA_001606085.1	GCF_000146605.3_Turkey_5.1_genomic.gff.gz
anoCar2.0	*Anolis carolinensis*	GCA_000090745.2	GCF_000090745.1_AnoCar2.0_genomic.gff.gz
aptFor1	*Aptenodytes forsteri*	GCA_000699145.1	
astMex2.0	*Astyanax mexicanus*	GCA_000372685.2	GCF_000372685.2_Astyanax_mexicanus-2.0_genomic.gff.gz
balBon1	*Balaenoptera bonaerensis*	GCA_000978805.1	GCA_000978805.1_ASM97880v1_genomic.gff.gz
balReg1	*Balearica regulorum*	GCA_000709895.1	
bosTau1.2	*Bos Taurus*	GCA_002263795.2	
braFlo2	*Branchiostoma floridae*	GCA_000003815.1	
bucRhi1	*Buceros rhinoceros*	GCA_000710305.1	
calPeg1	*Calidris pugnax*	GCA_001431845.1	
calMil6.1.3	*Callorhinchus milii*	GCA_000165045.2	GCF_000165045.1_Callorhinchus_milii-6.1.3_genomic.gff.gz
calAnn1	*Calypte anna*	GCA_003957555.2	
catAur1	*Cathartes aura*	GCA_000699945.1	
chiPla1	*Chiloscyllium plagiosum*	GCA_004010195.1	
chrPic3.0.3	*Chrysemys picta*	GCA_000241765.2	GCF_000241765.3_Chrysemys_picta_bellii-3.0.3_genomic.gff.gz
cioIntKH	*Ciona intestinalis*	GCA_000224145.2	
cluHar1	*Clupea harengus*	GCA_900323705.1	GCF_900700415.1_Ch_v2.0.2_genomic.gff.gz
colLiv1.0	*Columba livia*	GCA_000337935.1	
ConCri1.0	*Condylura cristata*	GCA_000260355.1	GCF_000260355.1_ConCri1.0_genomic.gff.gz
corAlt1	*Corapipo altera*	GCA_003945725.1	
corBra1	*Corvus brachyrhynchos*	GCA_000691975.1	
croPor1	*Crocodylus porosus*	GCA_001723895.1	GCF_001723895.1_CroPor_comp1_genomic.gff.gz
cucCan1	*Cuculus canorus*	GCA_000709325.1	
cynSem1.0	*Cynoglossus semilaevis*	GCA_000523025.1	GCF_000523025.1_Cse_v1.0_genomic.gff.gz
cypCar	*Cyprinus carpio*	GCA_000951615.2	GCF_000951615.1_common_carp_genome_genomic.gff.gz
danRer11	*Danio rerio*	GCA_000002035.4	GCF_000002035.6_GRCz11_genomic.gff.gz
dasNov3	*Dasypus novemcinctus*	GCA_000208655.2	GCF_000208655.1_Dasnov3.0_genomic.gff.gz
delLeu2	*Delphinapterus leucas*	GCA_002288925.3	
egrGar1	*Egretta garzetta*	GCA_000687185.1	

Efet.01	*Eisenia fetida*	GCA_003999395.1	
EleEdw1.0	*Elephantulus edwardii*	GCA_000299155.1	
equCab3.0	*Equus caballus*	GCA_002863925.1	GCF_002863925.1_EquCab3.0_genomic.gff.gz
eriEur2	*Erinaceus europaeus*	GCA_000296755.1	GCF_000296755.1_EriEur2.0_genomic.gff.gz
esoLuc3	*Esox lucius*	GCA_000721915.3	GCF_004634155.1_Eluc_v4_genomic.gff.gz
eurHel1	*Eurypyga helias*	GCA_000690775.1	
galGal6a	*Gallus gallus*	GCA_000002315.5	
gavGan1	*Gavialis gangeticus*	GCA_001723915.1	GCF_001723915.1_GavGan_comp1_genomic.gff.gz
gekJap1.1	*Gekko japonicus*	GCA_001447785.1	GCF_001447785.1_Gekko_japonicus_V1.1_genomic.gff.gz
halLeu4.0	*Haliaeetus leucocephalus*	GCA_000737465.1	
hetGla2	*Heterocephalus glaber*	GCA_000247695.1	
hipCam1	*Hippocampus comes*	GCA_001891065.1	GCF_001891065.1_H_comes_QL1_v1_genomic.gff.gz
lagObl1	*Lagenorhynchus obliquidens*	GCA_003676395.1	
lipVex1	*Lipotes vexillifer*	GCA_000442215.1	
loxAfr3	*Loxodonta Africana*	GCA_000001905.1	GCF_000001905.1_Loxafr3.0_genomic.gff.gz
melGall5.0	*Meleagris gallopavo*	GCA_000146605.4	GCF_000146605.3_Turkey_5.1_genomic.gff.gzAnguilla_rostrata
melUnd6.3	*Melopsittacus undulatus*	GCA_000238935.1	
merNub1	*Merops nubicus*	GCA_000691845.1	
nsch1	*Neomonachus schauinslandi*	GCA_002201575.1	GCF_002201575.1_ASM220157v1_genomic.gff.gz
notScu2	*Notechis scutatus*	GCA_900518725.1	GCF_900518725.1_TS10Xv2-PRI_genomic.gff.gz
numMel1.0	*Numida meleagris*	GCA_002078875.2	
opiTho1	*Opisthocomus hoazin*	GCA_000692075.1	
OfaDov1	*Orbicella faveolata*	GCA_002042975.1	
orcOrc1.1	*Orcinus orca*	GCA_000331955.2	
ornAna1.p.v1	*Ornithorhynchus anatinus*	GCA_004115215.2	GCF_004115215.1_mOrnAna1.p.v1_genomic.gff.gz
OryAfe1.0	*Orycteropus afer afer*	GCA_000298275.1	
oryLat1	*Oryzias latipes*	GCA_002234675.1	GCF_002234675.1_ASM223467v1_genomic.gff.gz
parMaj1.1	*Parus major*	GCA_001522545.3	
pelCri1	*Pelecanus crispus*	GCA_000687375.1	
pelSin1.0	*Pelodiscus sinensis*	GCA_000230535.1	GCF_000230535.1_PelSin_1.0_genomic.gff.gz
petMar1.0	*Petromyzon marinus*	GCA_002833325.1	
phaCar1	*Phalacrocorax carbo*	GCA_000708925.1	
phaCin4.1	*Phascolarctos cinereus*	GCA_002099425.1	GCF_002099425.1_phaCin_unsw_v4.1_genomic.gff.gz

phoRub1	*Phoenicopterus ruber*	GCA_000687265.1	
phyCat1	*Physeter catodon*	GCA_002837175.2	GCF_002837175.2_ASM283717v2_genomic.gff.gz
podCri1	*Podiceps cristatus*	GCA_000699545.1	
pomCan1	*Pomacea canaliculate*	GCA_003073045.1	
proCap1	*Procavia capensis*	GCA_004026925.2	
pseNaj2	*Pseudonaja textilis*	GCA_900518735.1	GCF_900518735.1_EBS10Xv2-PRI_genomic.gff.gz
pytBiv5.0.2	*Python bivittatus*	GCA_000186305.2	GCF_000186305.1_Python_molurus_bivittatus-5.0.2_genomic.gff.gz
ramVar4.0	*Ramazzottius varieornatus*	GCA_001949185.1	
rhiTyp2	*Rhincodon typus*	GCA_001642345.2	GCF_001642345.1_ASM164234v2_genomic.gff.gz
salSal2	*Salmo salar*	GCA_000233375.4	GCF_000233375.1_ICSASG_v2_genomic.gff.gz
strCam1	*Struthio camelus*	GCA_000698965.1	
susScr11.1	*Sus scrofa*	GCA_000003025.6	
tauEry1	*Tauraco erythrolophus*	GCA_000709365.1	
thaSir1	*Thamnophis sirtalis*	GCA_001077635.2	GCF_001077635.1_Thamnophis_sirtalis-6.0_genomic.gff.gz
tinGut2	*Tinamus guttatus*	GCA_000705375.2	
turTru1	*Tursiops truncatus*	GCA_001922835.1	
tytAlb1	*Tyto alba*	GCA_000687205.1	
aRhiBiv1.1	*Rhinatrema bivittatum*	GCA_901001135.1	GCF_901001135.1_aRhiBiv1.1_genomic.gff.gz
nanPar1	*Nanorana parkeri*	GCA_000935625.1	GCF_000935625.1_ASM93562v1_genomic.gff.gz
RCv2.1	*Lithobates catesbeianus*	GCA_002284835.2	GCA_002284835.2_RCv2.1_genomic.gff.gz
xenTro10	*Xenopus tropicalis*	GCA_000004195.4	GCF_000004195.4_UCB_Xtro_10.0_genomic.gff.gz
TaeGut1	*Zebra finch*	GCA_003957565.2	GCF_003957565.1_bTaeGut1_v1.p_genomic.gff.gz
