# Supplementary material for: Frequent birth-and-death events throughout perforin-1 evolution
Source: BMC Evol Biol. 2020 Oct 19;20:135. doi: 10.1186/s12862-020-01698-1 (PMC7574235; doi:10.1186/s12862-020-01698-1)
Supplement: Supplementary file 2 — Additional file 2 Perforin-1 loci in mammalian species. PRF1 genes are depicted to scale with intron/exon boundaries (blue boxes). Pseudogenes are depicted in pink. Flanking genes may be cropped for ease of depiction. [file 12862_2020_1698_MOESM2_ESM.pdf]

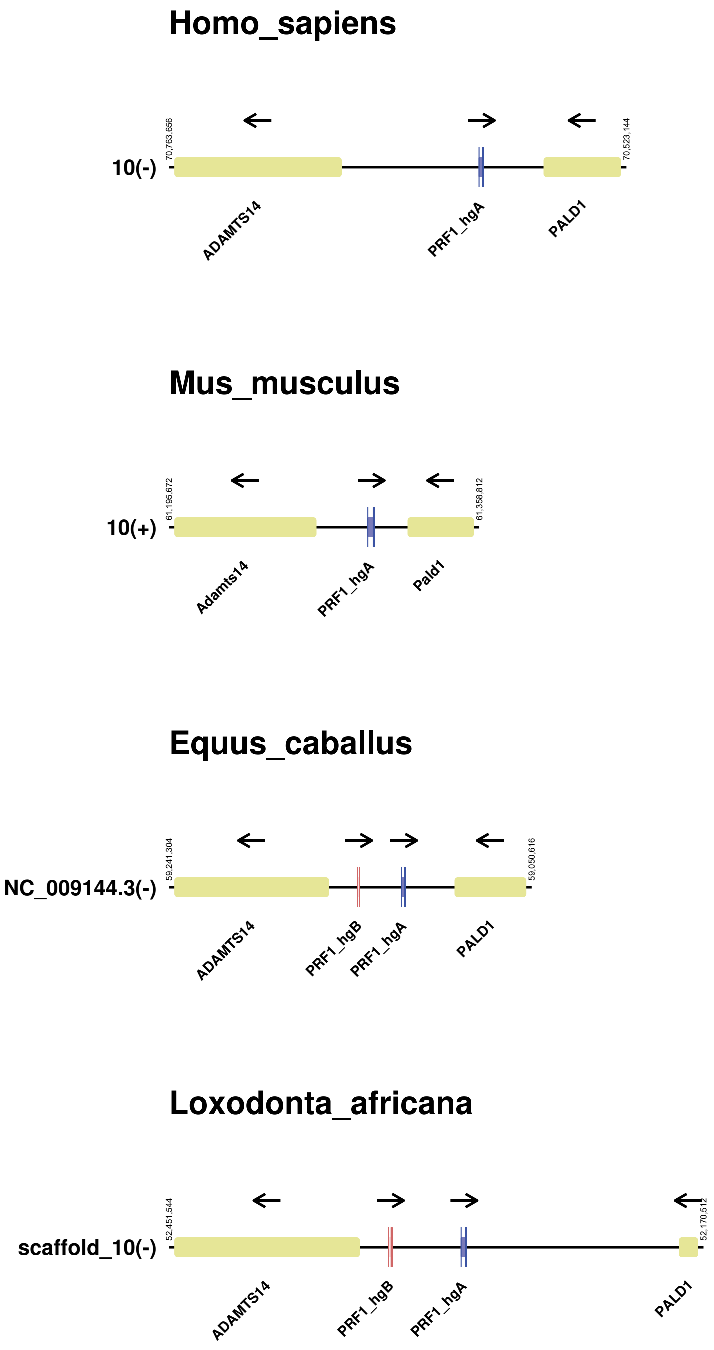
Homo_sapiens
10(-)
70,763,656
70,523,144
ADAMTS14
PRF1_hgA
PALD1
Mus_musculus
10(+)
61,195,672
61,358,812
Adamts14
PRF1_hgA
Pald1
Equus_caballus
NC_009144.3(-)
59,241,304
59,050,616
ADAMTS14
PRF1_hgB
PRF1_hgA
PALD1
Loxodonta_africana
scaffold_10(-)
52,451,544
52,170,512
ADAMTS14
PRF1_hgB
PRF1_hgA
PALD1

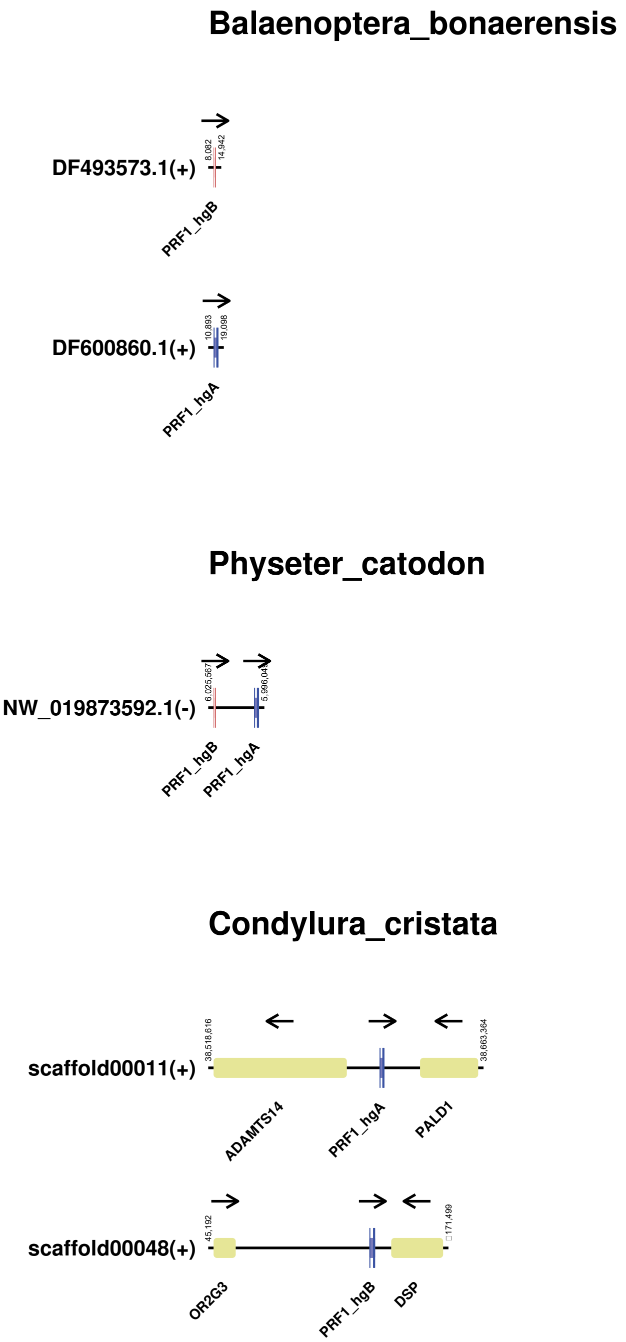

Balaenoptera_bonaerensis
DF493573.1(+)
8,082
14,942
PRF1_hgB
DF600860.1(+)
10,893
19,098
PRF1_hgA
Physeter_catodon
NW_019873592.1(-)
6,025,567
PRF1_hgB
PRF1_hgA
Condylura_cristata
scaffold00011(+)
38,518,616
38,663,364
ADAMTS14
PRF1_hgA
PALD1
scaffold00048(+)
45,192
171,499
OR2G3
PRF1_hgB
DSP

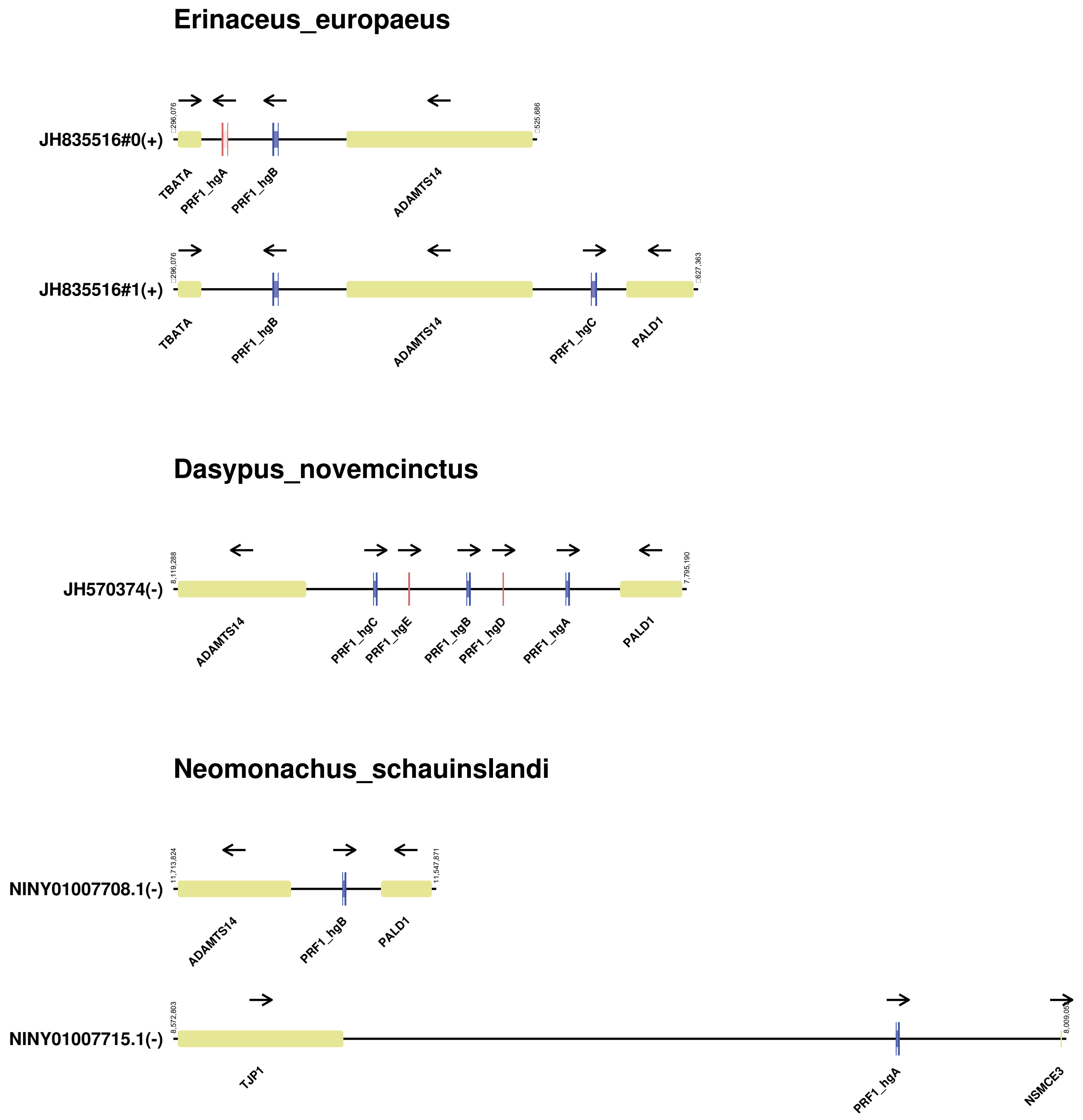
Erinaceus_europaeus
JH835516#0(+)
296,076
525,686
TBATA
PRF1_hgA
PRF1_hgB
ADAMTS14
JH835516#1(+)
296,076
627,363
TBATA
PRF1_hgB
ADAMTS14
PRF1_hgC
PALD1
Dasypus_novemcinctus
JH570374(-)
8,119,288
7,795,190
ADAMTS14
PRF1_hgC
PRF1_hgE
PRF1_hgB
PRF1_hgD
PRF1_hgA
PALD1
Neomonachus_schauinslandi
NINY01007708.1(-)
11,713,824
11,547,871
ADAMTS14
PRF1_hgB
PALD1
NINY01007715.1(-)
8,572,803
TJP1
PRF1_hgA
NSMCE3

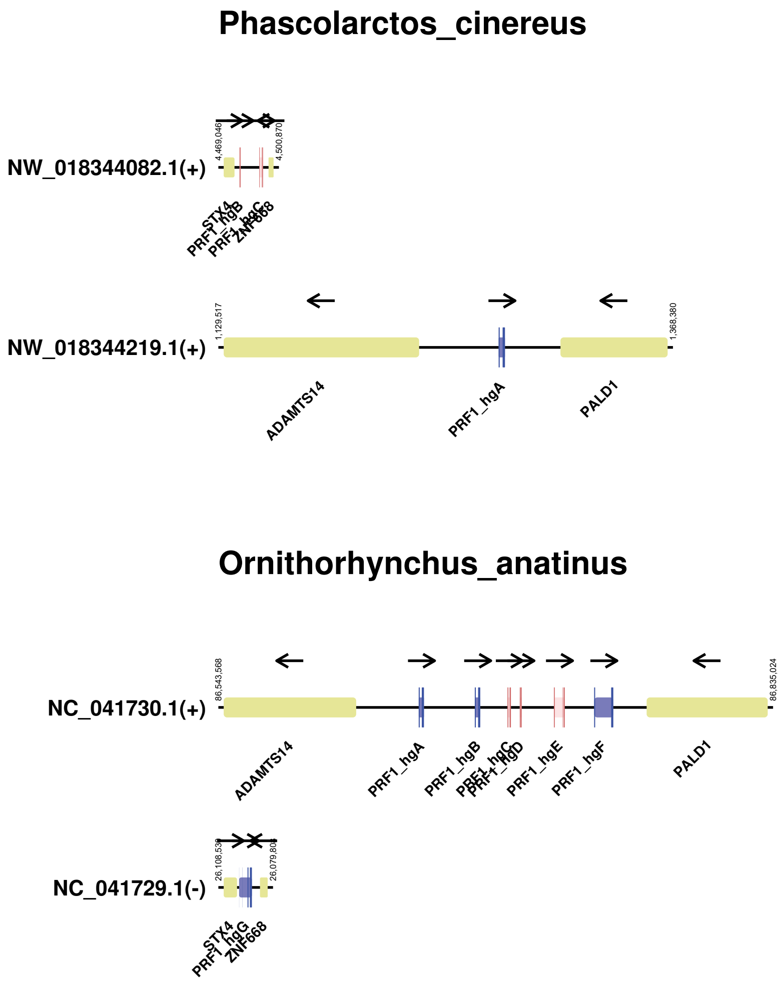
Phascolarctos_cinereus
NW_018344082.1(+)
4,469,046
4,500,870
STX4
PRF1_hgB
PRF1_hgC
ZNF668
NW_018344219.1(+)
1,129,517
1,368,380
ADAMTS14
PRF1_hgA
PALD1
Ornithorhynchus_anatinus
NC_041730.1(+)
86,543,568
86,835,024
ADAMTS14
PRF1_hgA
PRF1_hgB
PRF1_hgC
PRF1_hgD
PRF1_hgE
PRF1_hgF
PALD1
NC_041729.1(-)
STX4
PRF1_hgG
ZNF668
