# Supplementary material for: Frequent birth-and-death events throughout perforin-1 evolution
Source: BMC Evol Biol. 2020 Oct 19;20:135. doi: 10.1186/s12862-020-01698-1 (PMC7574235; doi:10.1186/s12862-020-01698-1)

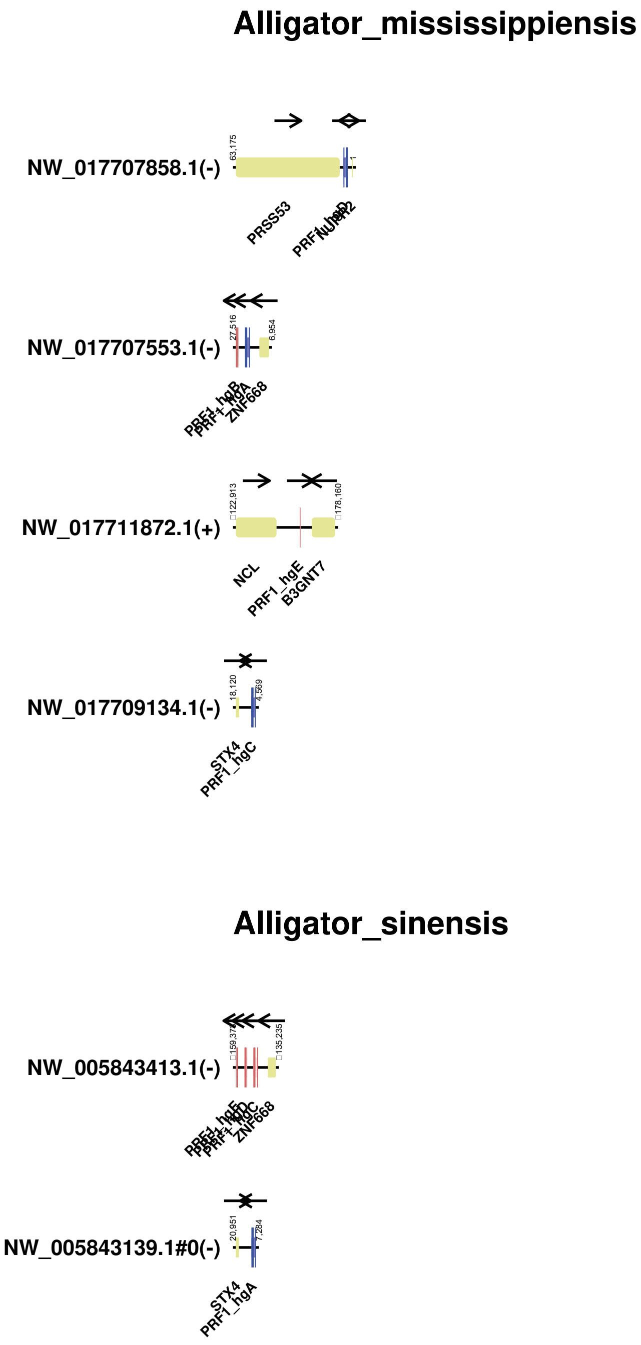

Alligator_mississippiensis
NW_017707858.1(-)
63,175
1
PRSS53
PRF1_hgD
NUPR2
NW_017707553.1(-)
27,516
6,954
PRF1_hgB
PRF1_hgA
ZNF668
NW_017711872.1(+)
122,913
178,160
NCL
PRF1_hgE
B3GNT7
NW_017709134.1(-)
18,120
4,569
STX4
PRF1_hgC
Alligator_sinensis
NW_005843413.1(-)
135,235
PRF1_hgC
ZNF668
NW_005843139.1#0(-)
20,951
7,284
STX4
PRF1_hgA

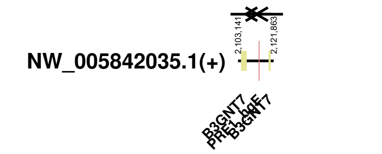

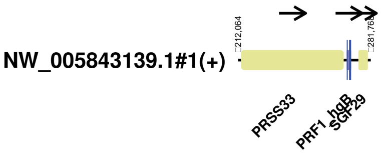

Crocodylus_porosus

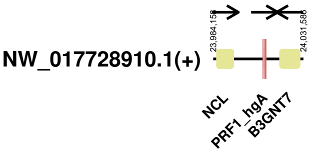

Gavialis_gangeticus

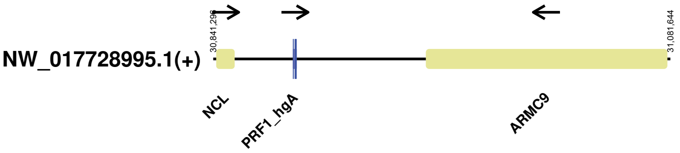

Chrysemys_picta

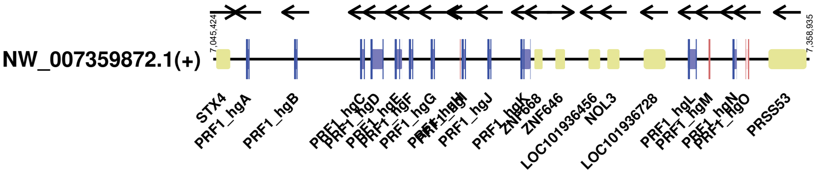

Pelodiscus_sinensis

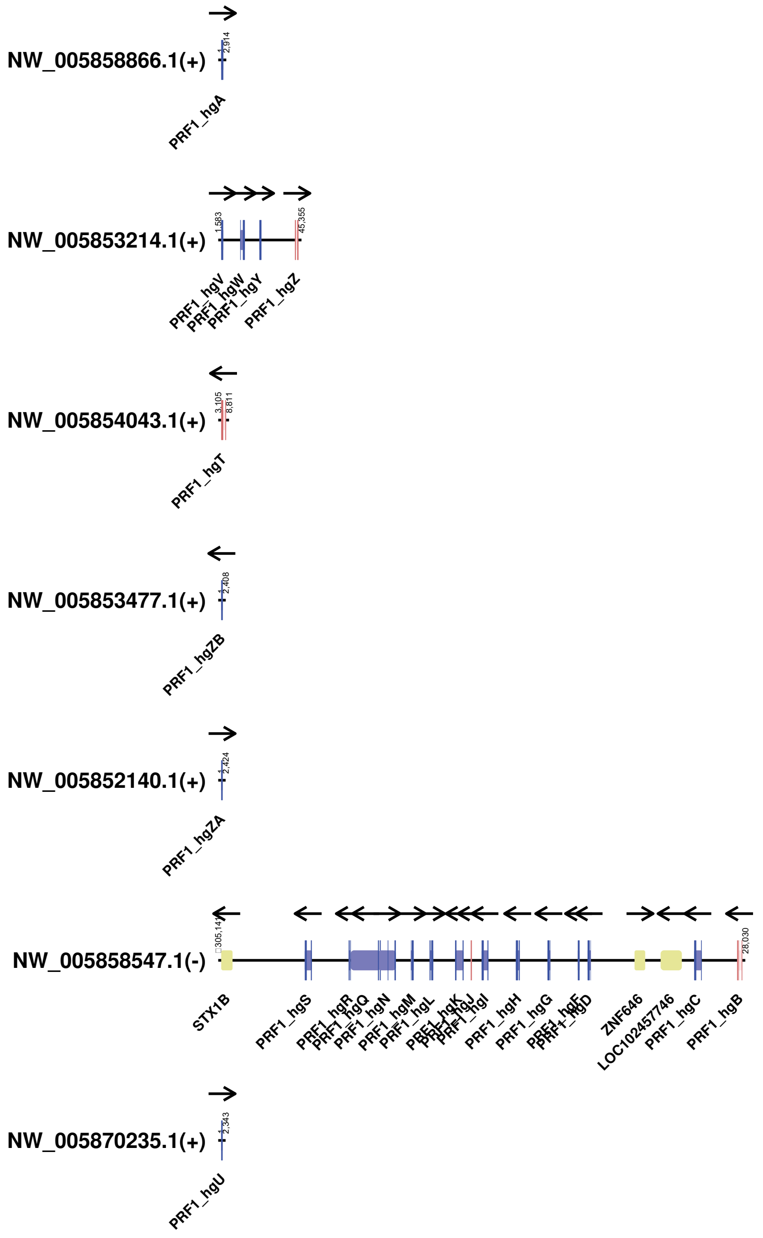

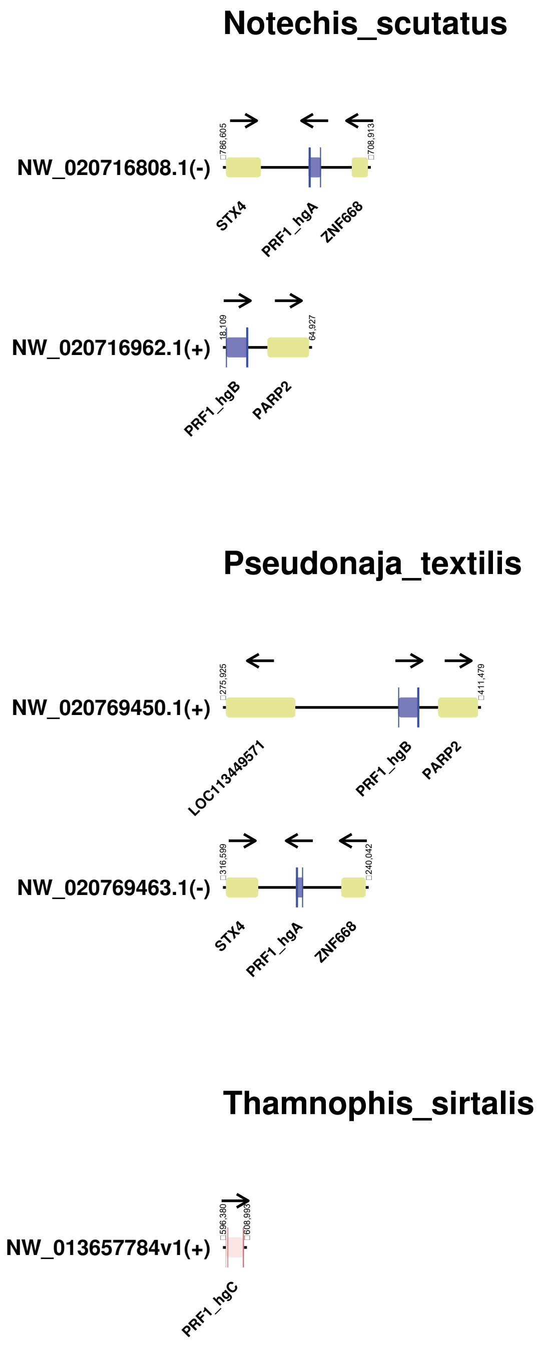
Notechis_scutatus
NW_020716808.1(-)
786,605
708,913
STX4
PRF1_hgA
ZNF668
NW_020716962.1(+)
18,109
64,927
PRF1_hgB
PARP2
Pseudonaja_textilis
NW_020769450.1(+)
275,925
411,479
LOC113449571
PRF1_hgB
PARP2
NW_020769463.1(-)
316,599
240,042
STX4
PRF1_hgA
ZNF668
Thamnophis_sirtalis
NW_013657784v1(+)
596,380
608,993
PRF1_hgC

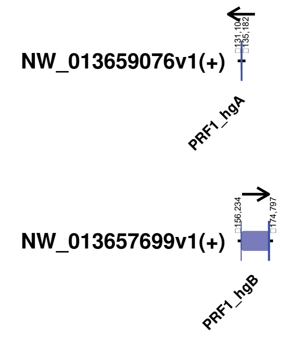

Python_bivittatus

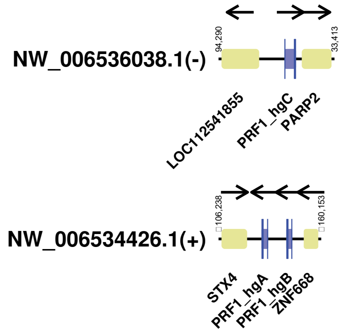

Anolis_carolinensis

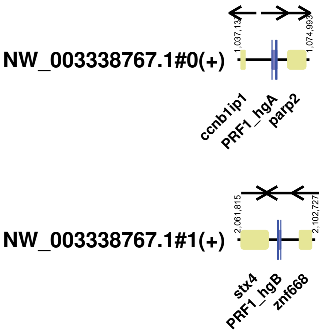

Gekko_japonicus

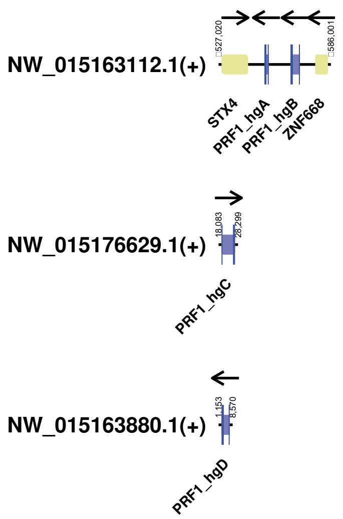

Supplement: Supplementary file 3 — Additional file 3 Perforin-1 loci in non-avian reptiles. PRF1 genes are depicted to scale with intron/exon boundaries (blue boxes). Pseudogenes are depicted in pink. Flanking genes may be cropped for ease of depiction. [file 12862_2020_1698_MOESM3_ESM.pdf]
