# Supplementary figures and images for: Frequent birth-and-death events throughout perforin-1 evolution
Source: BMC Evol Biol. 2020 Oct 19;20:135. doi: 10.1186/s12862-020-01698-1 (PMC7574235; doi:10.1186/s12862-020-01698-1)

Melleagris\_gallopavo

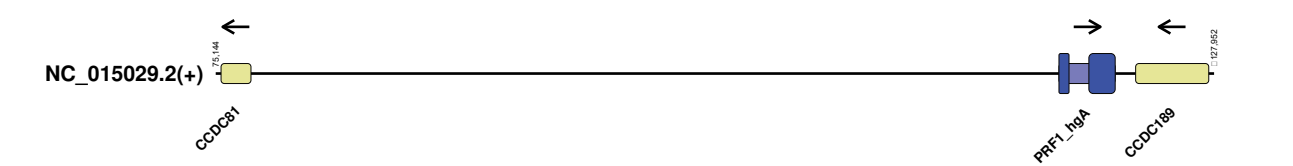

Zebra\_finch

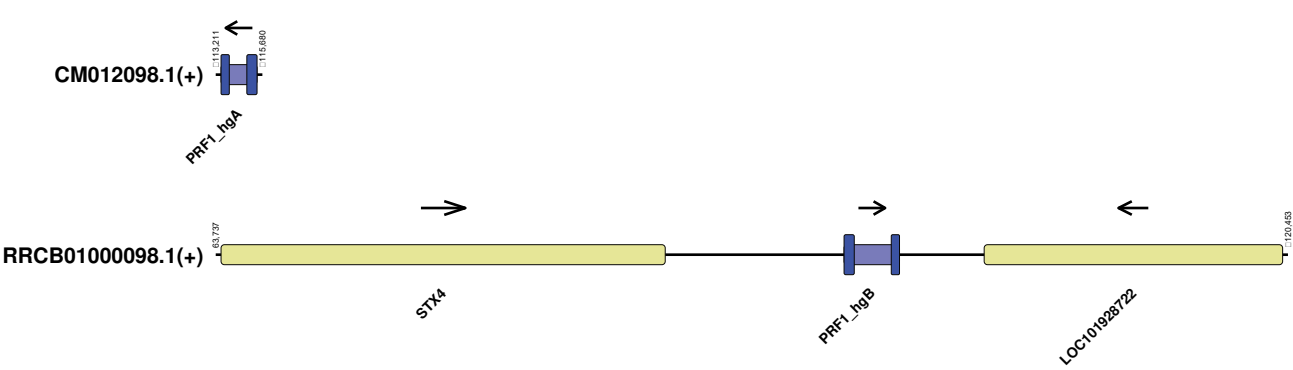

Gallus\_gallus

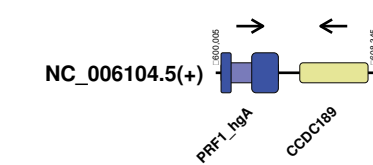

Supplement: Supplementary file 4 — Additional file 4 Perforin-1 loci in birds. PRF1 genes are depicted to scale with intron/exon boundaries (blue boxes). Pseudogenes are depicted in pink. Flanking genes may be cropped for ease of depiction. [file 12862_2020_1698_MOESM4_ESM.pdf]

Rhincodon\_typus

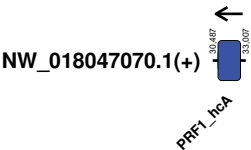

Chiloscyllium\_plagiosum

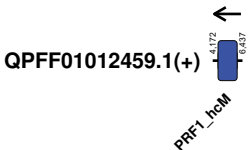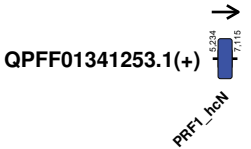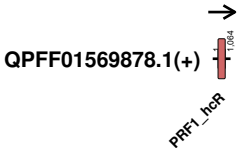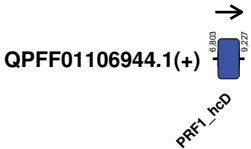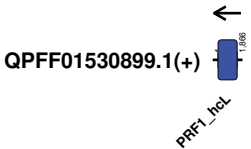

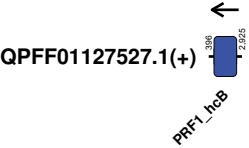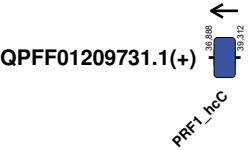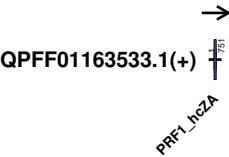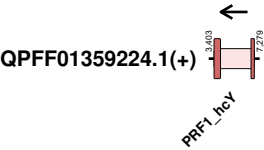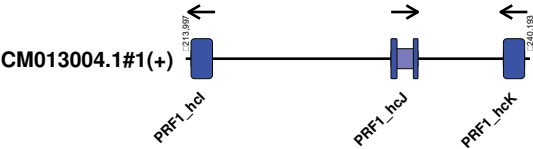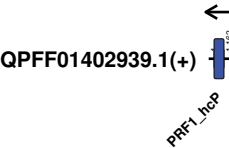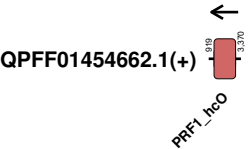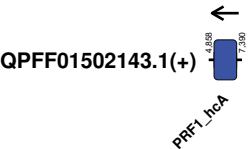

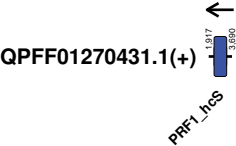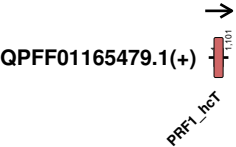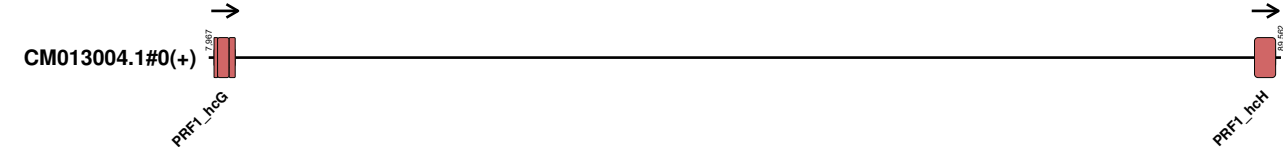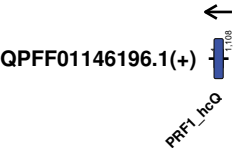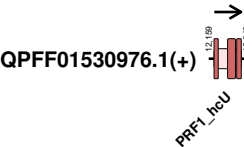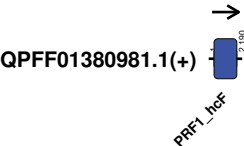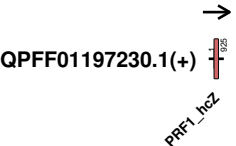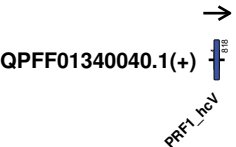

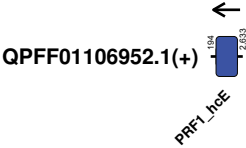

**Callorhinchus\_milii**

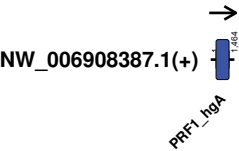

Supplement: Supplementary file 7 — Additional file 7 Perforin-1 loci in cartilaginous fishes. PRF1 genes are depicted to scale with intron/exon boundaries (blue boxes). Pseudogenes are depicted in pink. Flanking genes may be cropped for ease of depiction. [file 12862_2020_1698_MOESM7_ESM.pdf]

Bayesian inference

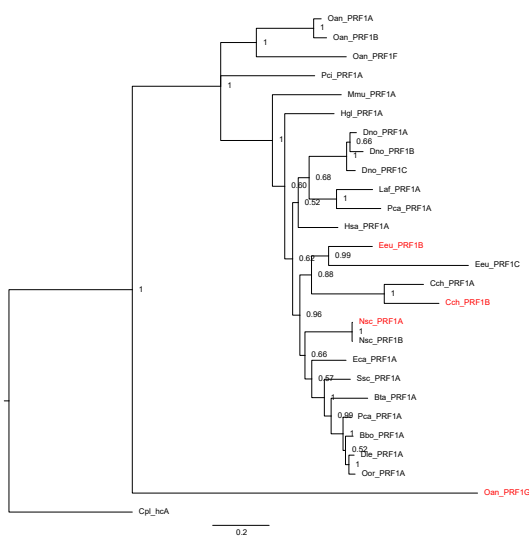

Parsimony

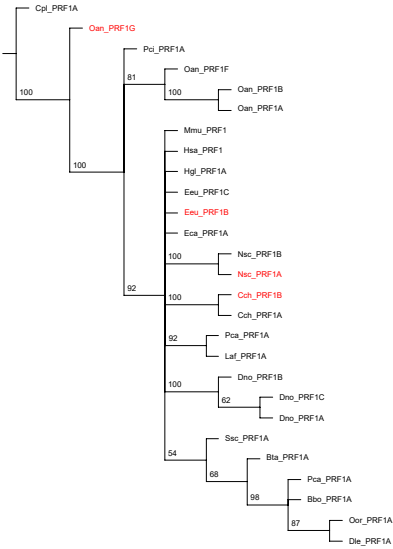

Supplement: Supplementary file 8 — Additional file 8 Phylogenetic relationships between mammalian PRF1 genes. Bayesian inference (left) and maximum-parsimony (right) phylogenetic trees from mammalian perforin-1 sequences. Names in red belong to genes in loci different from ADAMTS14-PALD1. Oan, Ornithorhynchus anatinus; Pci, Phascolarctos cinereus; Mmu, Mus musculus; Hgl, Heterocephalus glaber; Dno, Dasypus novemcinctus; Laf, Loxodonta africana; Pca, Procavia capensis; Eeu, Erinaceus europaeus; Hsa, Homo sapiens; Cch, Condylura cristata; Nsc, Neomonachus schauinslandi; Eca, Equus caballus; Ssc, Sus scrofa; Bta, Bos taurus; Pca, Physeter catodon; Bbo, Balaenoptera bonaerensis; Dle, Delphinapterus leucas; Oor, Orcinus orca; Cpl, Chiloscyllium plagiosum. [file 12862_2020_1698_MOESM8_ESM.pdf]

## Danio\_rerio

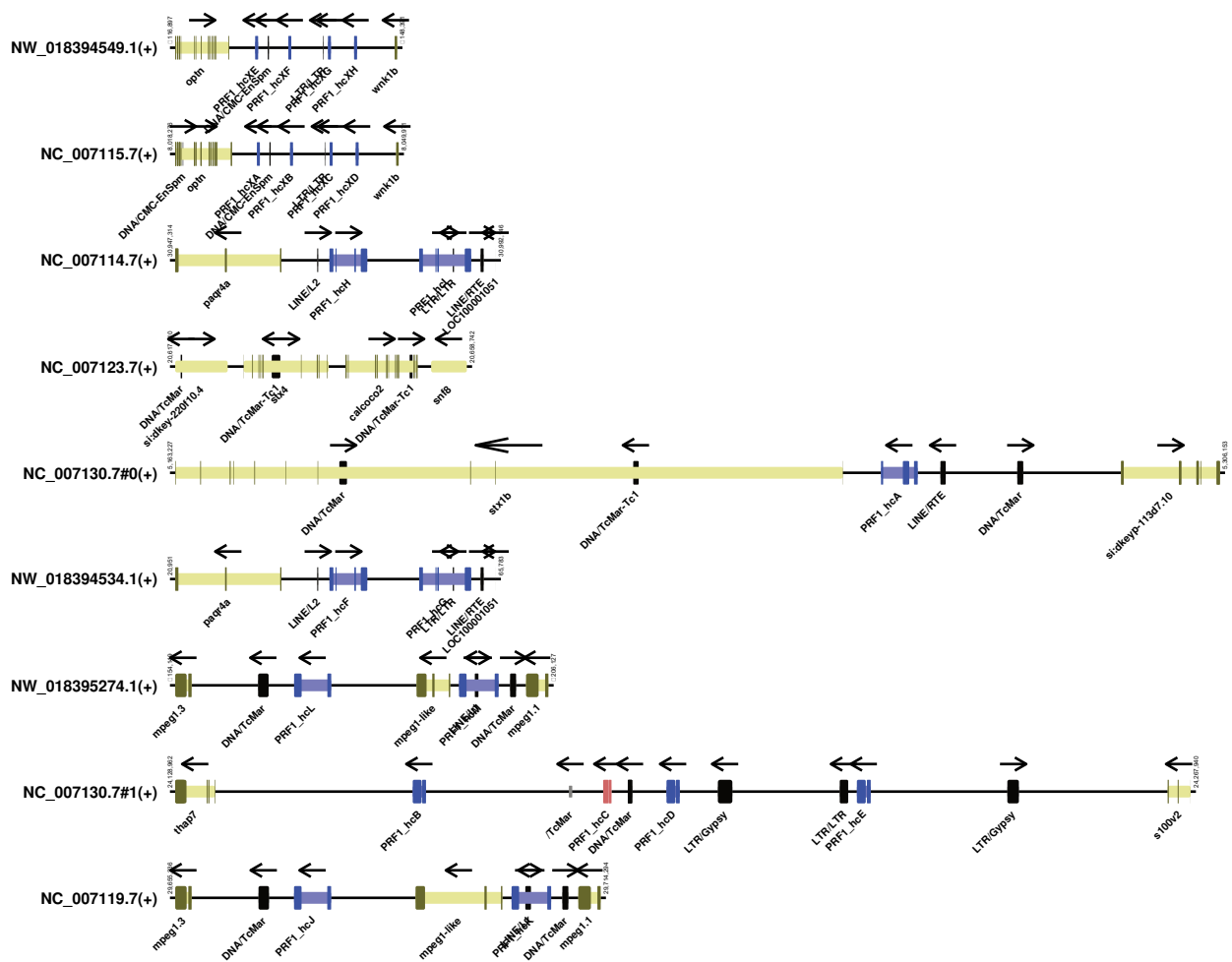

Oryzias\_latipes

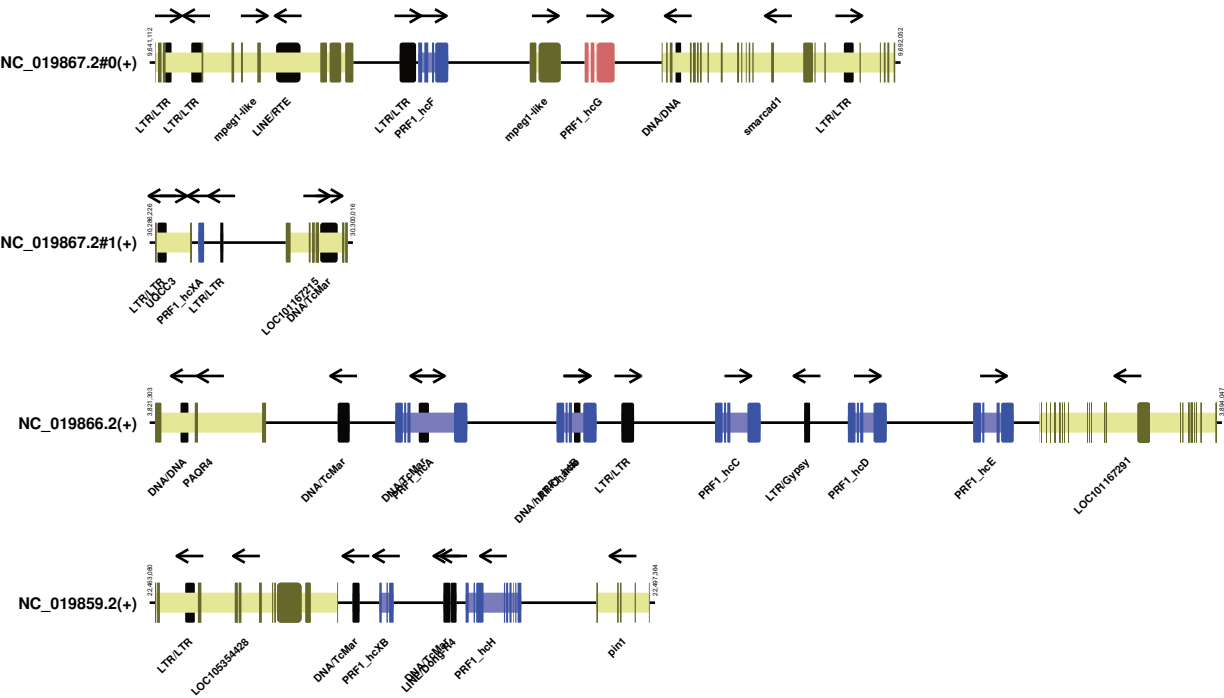

Supplement: Supplementary file 10 — Additional file 10 Transposon-like sequences in PRF1 loci of Danio rerio and Oryzias latipes. PRF1 genes are depicted to scale with intron/exon boundaries (blue boxes). Pseudogenes are depicted in pink. Transposon-like sequences are shown as black boxes. [file 12862_2020_1698_MOESM10_ESM.pdf]

# Chrysemys\_picta

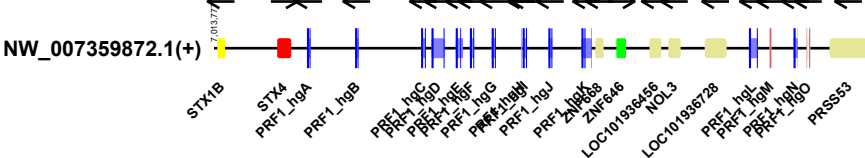

# Pelodiscus\_sinensis

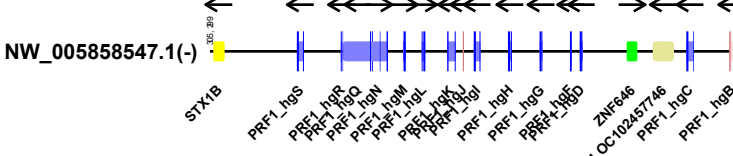

Supplement: Supplementary file 13 — Additional file 13 STX4-ZNF646 locus in turtles. PRF1 genes are depicted to scale with intron/exon boundaries (blue boxes). Pseudogenes are depicted in pink. Flanking genes may be cropped for ease of depiction. [file 12862_2020_1698_MOESM13_ESM.pdf]
