# Supplementary material for: Frequent birth-and-death events throughout perforin-1 evolution
Source: BMC Evol Biol. 2020 Oct 19;20:135. doi: 10.1186/s12862-020-01698-1 (PMC7574235; doi:10.1186/s12862-020-01698-1)
Supplement: Supplementary file 5 — Additional file 5 Perforin-1 loci in amphibians. PRF1 genes are depicted to scale with intron/exon boundaries (blue boxes). Pseudogenes are depicted in pink. Flanking genes may be cropped for ease of depiction. [file 12862_2020_1698_MOESM5_ESM.pdf]

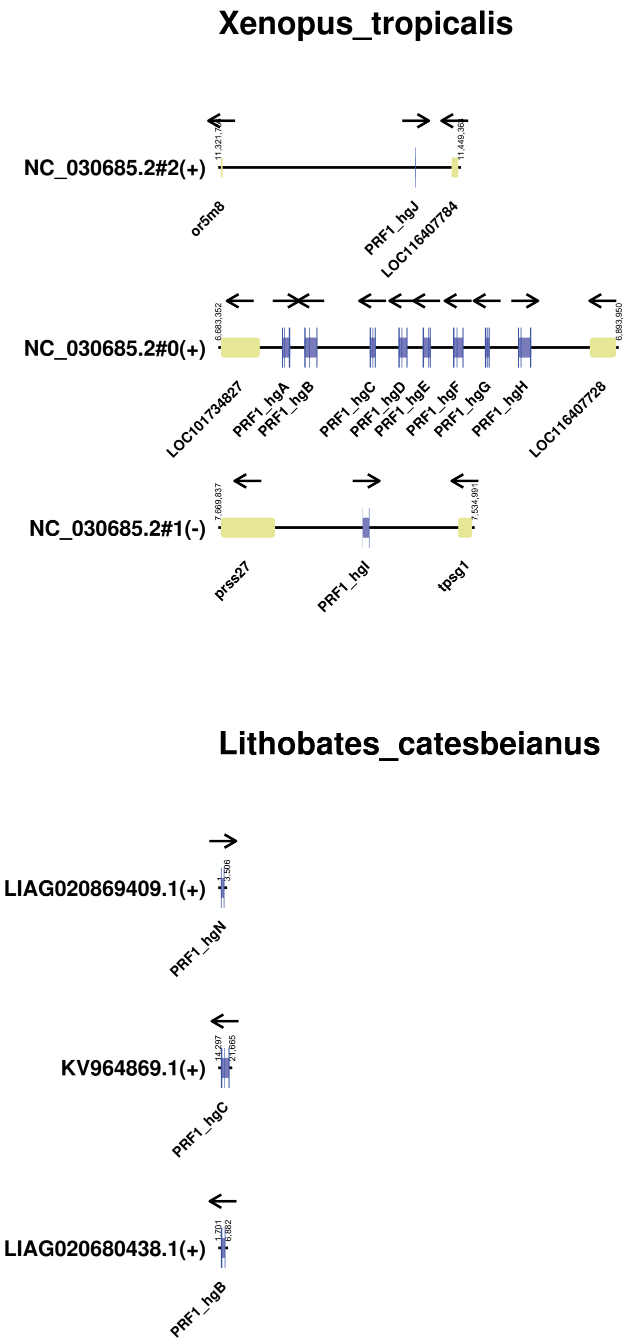

Xenopus_tropicalis
NC_030685.2#2(+)
or5m8
PRF1_hgJ
LOC116407784
NC_030685.2#0(+)
6,683,352
6,893,950
LOC101734827
PRF1_hgA
PRF1_hgB
PRF1_hgC
PRF1_hgD
PRF1_hgE
PRF1_hgF
PRF1_hgG
PRF1_hgH
LOC116407728
NC_030685.2#1(-)
7,669,837
7,534,991
prss27
PRF1_hgI
tpsg1
Lithobates_catesbeianus
LIAG020869409.1(+)
3,506
PRF1_hgN
KV964869.1(+)
14,297
21,665
PRF1_hgC
LIAG020680438.1(+)
1,701
6,882
PRF1_hgB

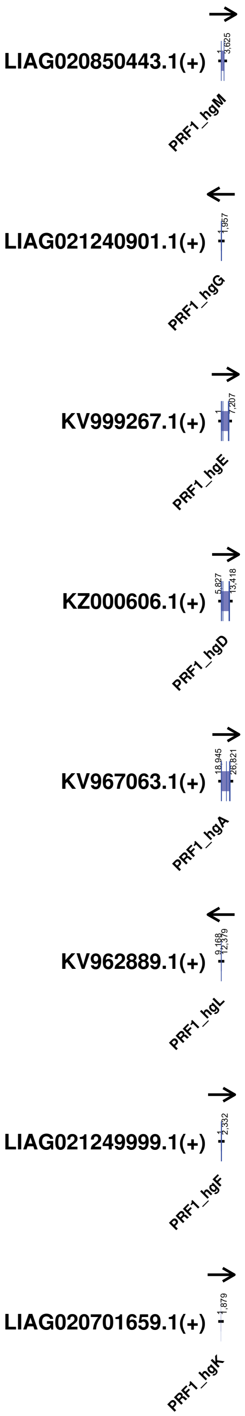

LIAG020850443.1(+)
3,625
PRF1_hgM
LIAG021240901.1(+)
1,957
PRF1_hgG
KV999267.1(+)
7,207
PRF1_hgE
KZ000606.1(+)
5,827
13,418
PRF1_hgD
KV967063.1(+)
18,945
26,821
PRF1_hgA
KV962889.1(+)
12,379
PRF1_hgL
LIAG021249999.1(+)
2,332
PRF1_hgF
LIAG020701659.1(+)
1,879
PRF1_hgK

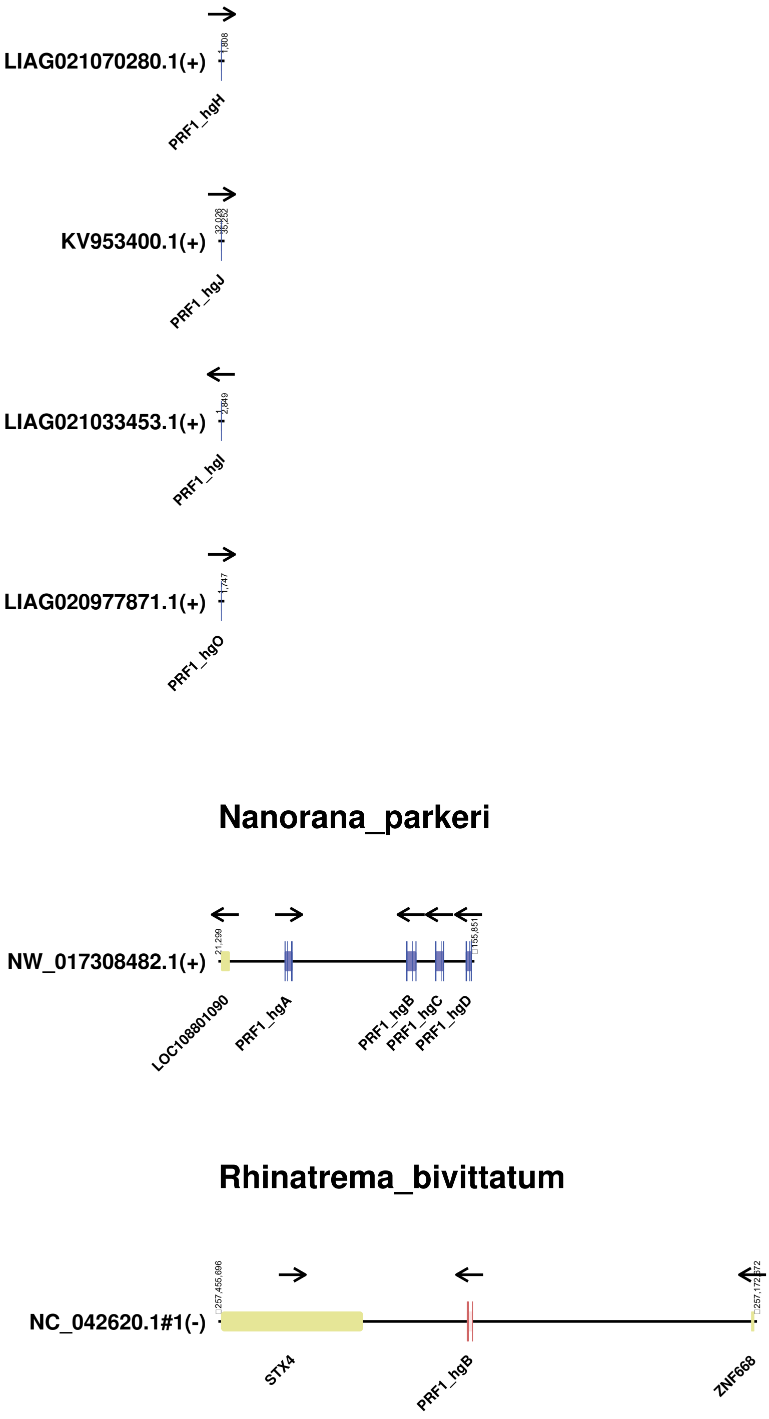
LIAG021070280.1(+)
PRF1_hgH
KV953400.1(+)
PRF1_hgJ
LIAG021033453.1(+)
PRF1_hgI
LIAG020977871.1(+)
PRF1_hgO
Nanorana_parkeri
NW_017308482.1(+)
LOC108801090
PRF1_hgA
PRF1_hgB
PRF1_hgC
PRF1_hgD
Rhinatrema_bivittatum
NC_042620.1#1(-)
STX4
PRF1_hgB
ZNF668

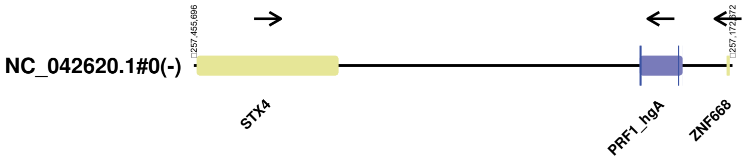
NC_042620.1#0(-)
257,455,696
STX4
PRF1_hgA
ZNF668
