# Supplementary material for: Frequent birth-and-death events throughout perforin-1 evolution
Source: BMC Evol Biol. 2020 Oct 19;20:135. doi: 10.1186/s12862-020-01698-1 (PMC7574235; doi:10.1186/s12862-020-01698-1)
Supplement: Supplementary file 6 — Additional file 6 Perforin-1 loci in teleosts. PRF1 genes are depicted to scale with intron/exon boundaries (blue boxes). Pseudogenes are depicted in pink. Genes with names containing hcX belong to the c2PRF1 family. Flanking genes may be cropped for ease of depiction. [file 12862_2020_1698_MOESM6_ESM.pdf]

Salmo_salar

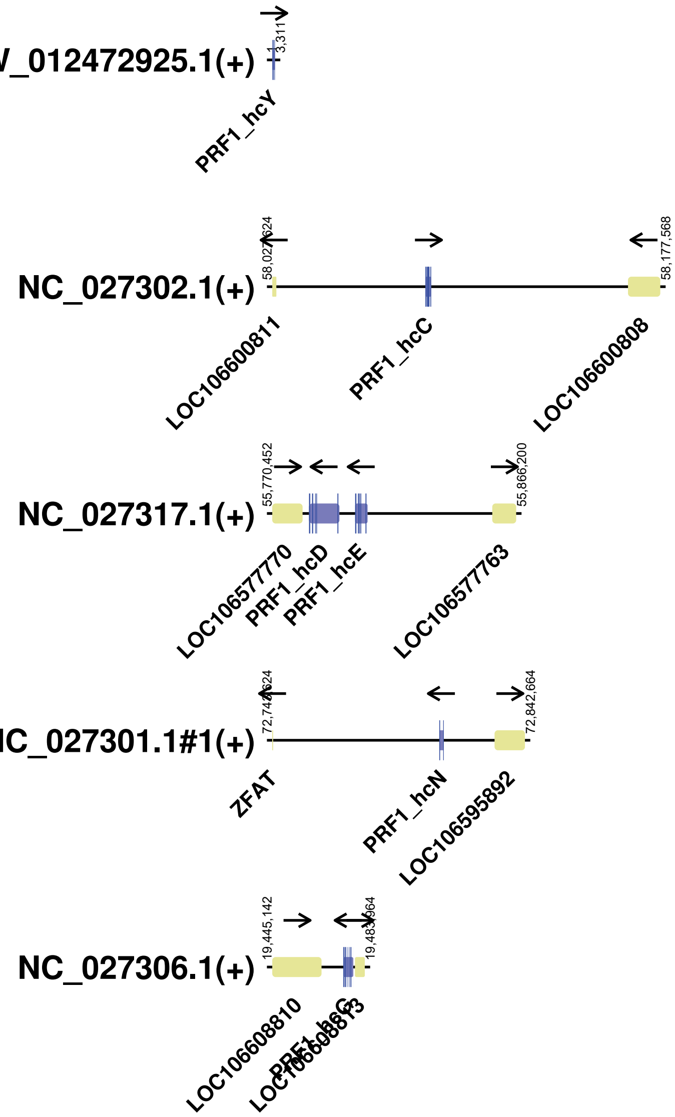

_012472925.1(+)
3,311
PRF1_hcY
NC_027302.1(+)
58,177,568
LOC106600811
PRF1_hcC
LOC106600808
NC_027317.1(+)
55,770,452
55,866,200
LOC106577770
PRF1_hcD
PRF1_hcE
LOC106577763
IC_027301.1#1(+)
72,842,664
ZFAT
PRF1_hcN
LOC106595892
NC_027306.1(+)
19,445,142
LOC106608810

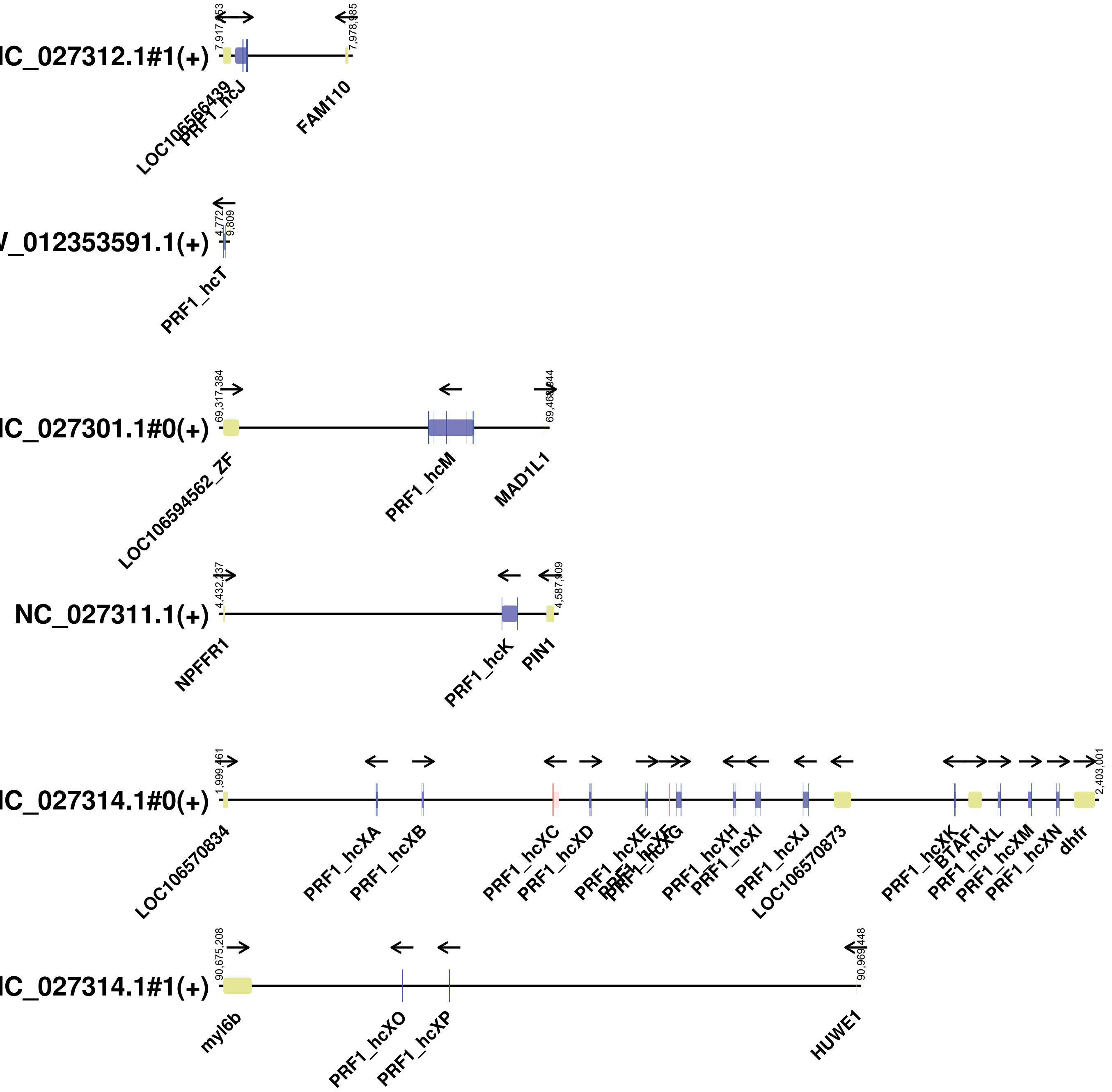

IC_027312.1#1(+)
7,917,463
7,978,985
LOC106566439
PRF1_hcJ
FAM110
/_012353591.1(+)
4,772
9,809
PRF1_hcT
IC_027301.1#0(+)
69,317,384
LOC106594562_ZF
PRF1_hcM
MAD1L1
NC_027311.1(+)
4,432,137
4,587,909
NPFFR1
PRF1_hcK
PIN1
IC_027314.1#0(+)
1,999,461
2,403,001
LOC106570834
PRF1_hcXA
PRF1_hcXB
PRF1_hcXC
PRF1_hcXD
PRF1_hcXE
PRF1_hcXH
PRF1_hcXI
PRF1_hcXJ
LOC106570873
PRF1_hcXK
BTAF1
PRF1_hcXL
PRF1_hcXM
PRF1_hcXN
dhfr
IC_027314.1#1(+)
90,675,208
90,969,448
myl6b
PRF1_hcXO
PRF1_hcXP
HUWE1

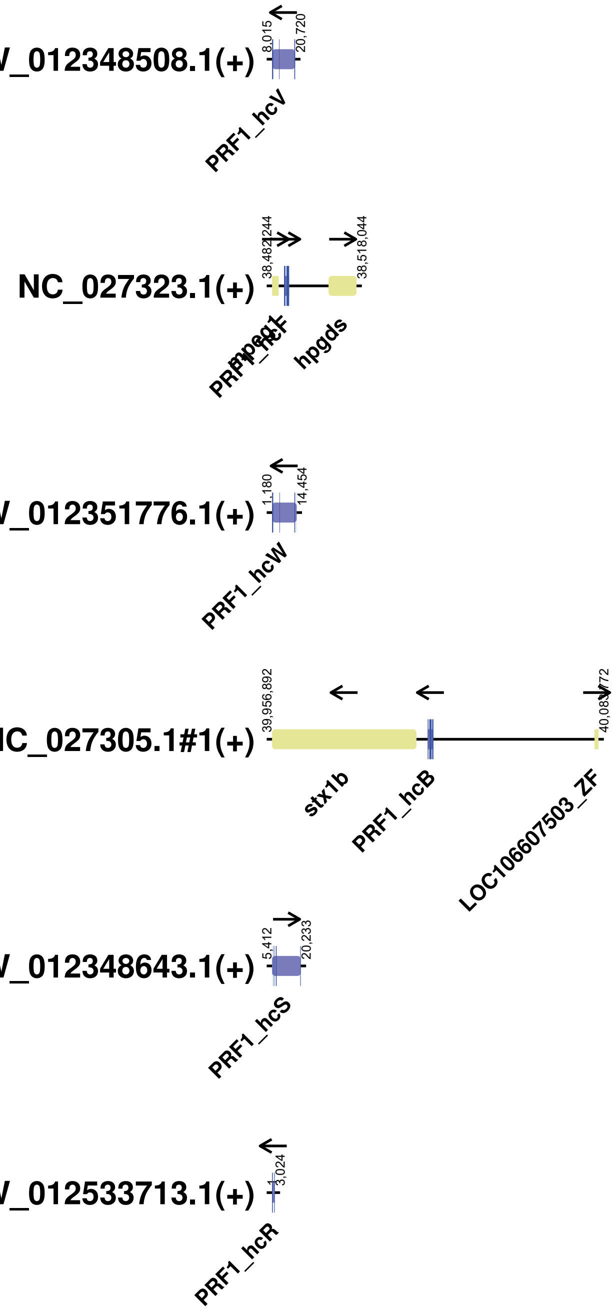

_012348508.1(+)
8,015
20,720
PRF1_hcV
NC_027323.1(+)
38,518,044
PRF1_hcF
hpgds
_012351776.1(+)
1,180
14,454
PRF1_hcW
C_027305.1#1(+)
39,956,892
stx1b
PRF1_hcB
LOC106607503_ZF
_012348643.1(+)
5,412
20,233
PRF1_hcS
_012533713.1(+)
3,024
PRF1_hcR

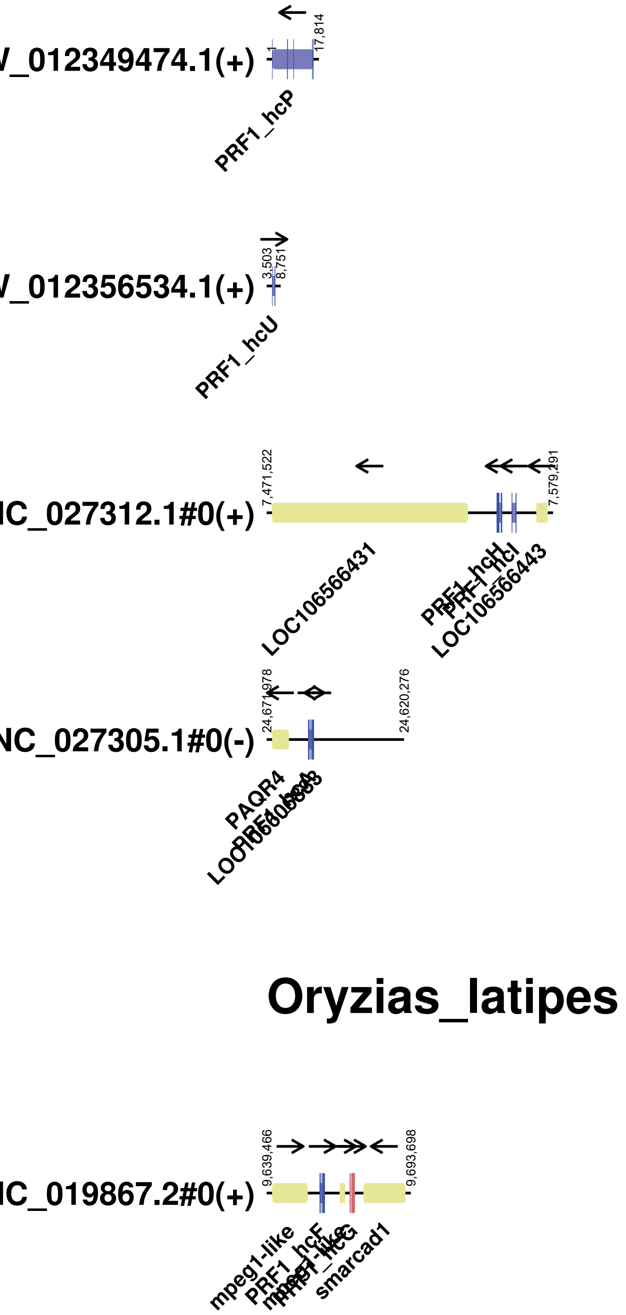

_012349474.1(+)
17,814
PRF1_hcP
_012356534.1(+)
3,503
8,751
PRF1_hcU
C_027312.1#0(+)
7,471,522
7,579,191
LOC106566431
PRF1_hcH
PRF1_hcI
LOC106566443
C_027305.1#0(-)
24,671,978
24,620,276
PAQR4
Oryzias_latipes
C_019867.2#0(+)
9,639,466
9,693,698
mpeg1-like
PRF1_hcF
PRF1_hcG
smarcad1

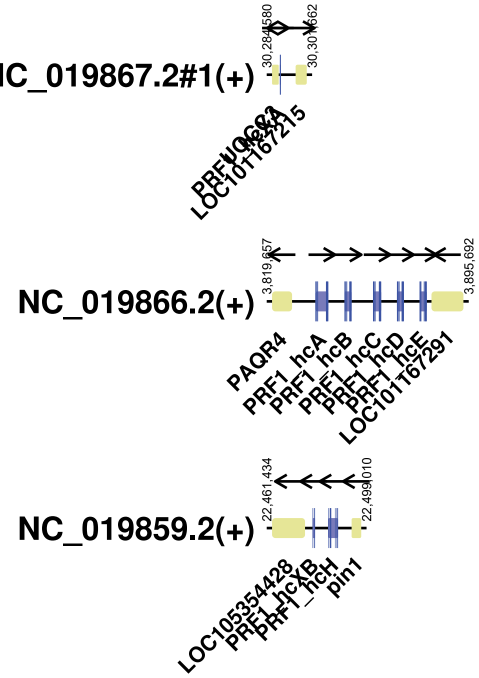

Hippocampus_comes

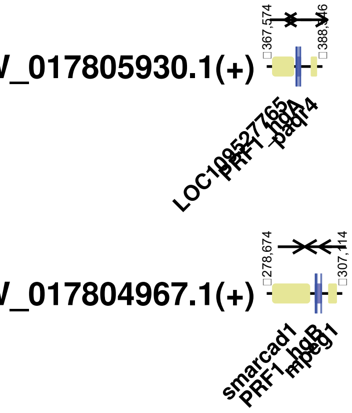

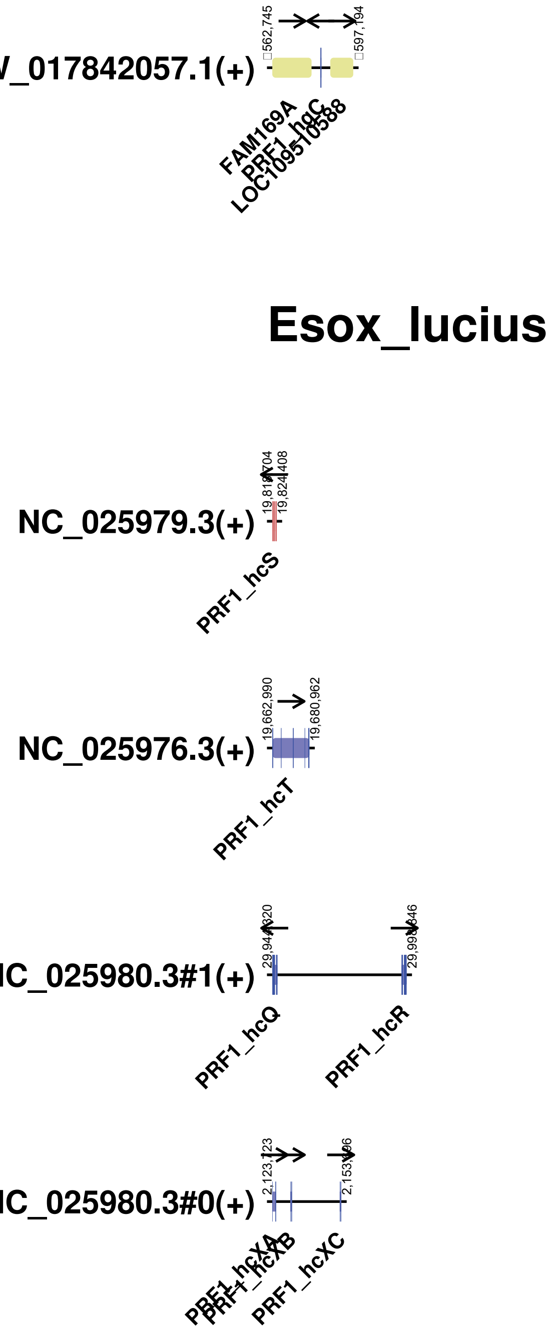
_017842057.1(+)
562,745
597,194
FAM169A
PRF1_hqC
LOC109510588
Esox_lucius
NC_025979.3(+)
PRF1_hcS
NC_025976.3(+)
19,662,990
19,680,962
PRF1_hcT
NC_025980.3#1(+)
PRF1_hcQ
PRF1_hcR
NC_025980.3#0(+)
PRF1_hcXA
PRF1_hcXB
PRF1_hcXC

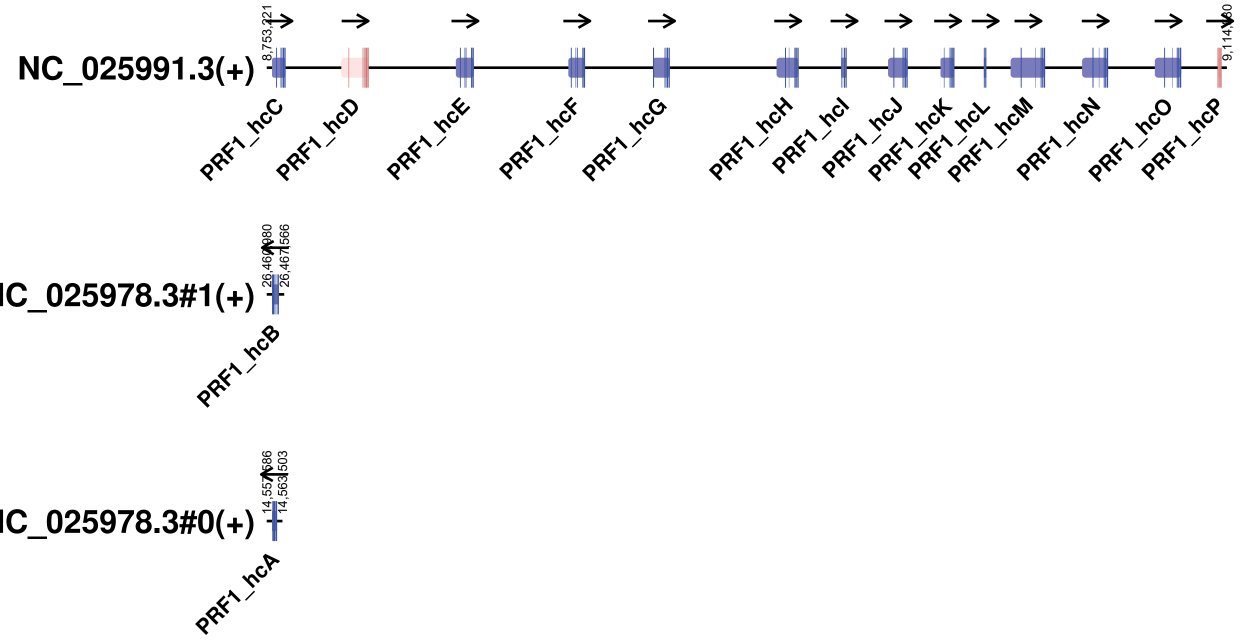
NC_025991.3(+)
8,753,321
9,114,130
PRF1_hcC
PRF1_hcD
PRF1_hcE
PRF1_hcF
PRF1_hcG
PRF1_hcH
PRF1_hcI
PRF1_hcJ
PRF1_hcK
PRF1_hcL
PRF1_hcM
PRF1_hcN
PRF1_hcO
PRF1_hcP
IC_025978.3#1(+)
26,467,566
PRF1_hcB
IC_025978.3#0(+)
14,563,503
PRF1_hcA

Danio_rerio

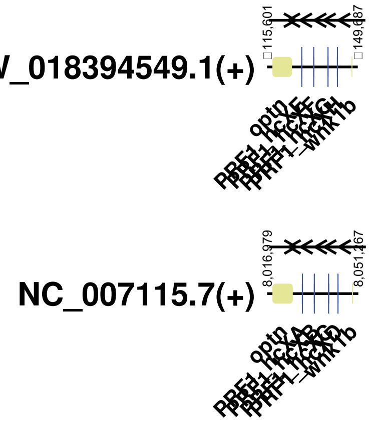
/_018394549.1(+)
115,601
149,187
NC_007115.7(+)
8,016,979
8,051,167

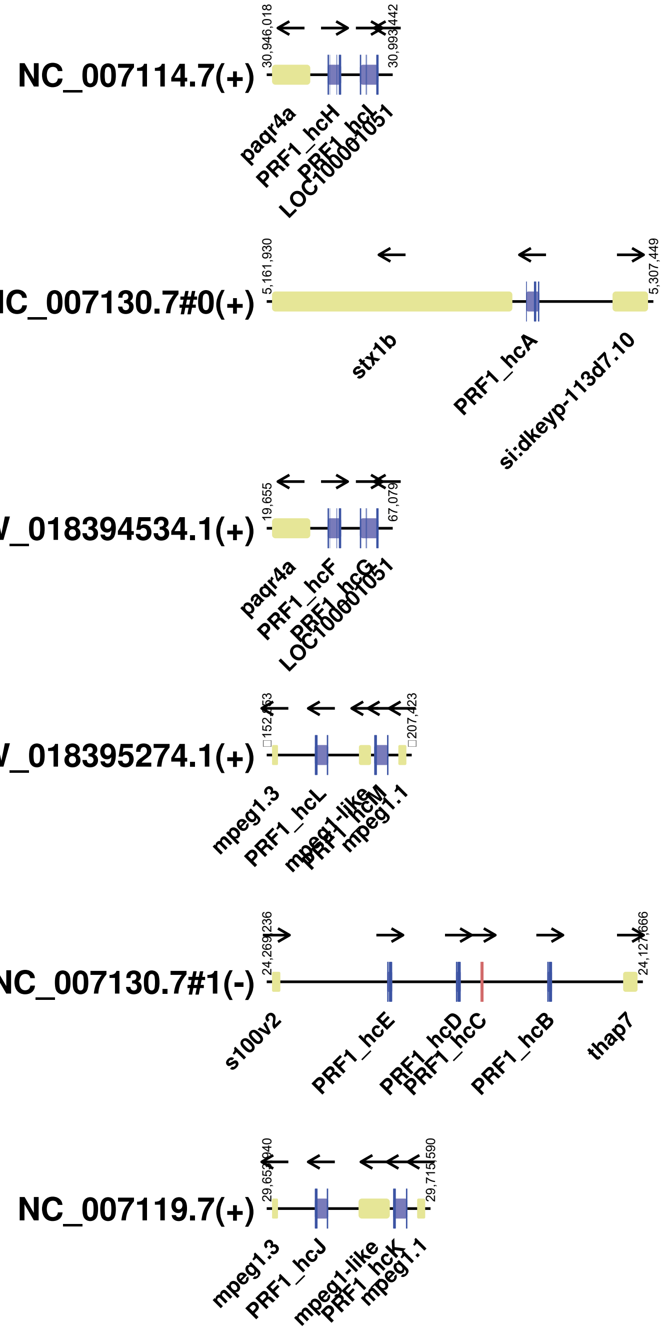
NC_007114.7(+)
30,946,018
30,993,442
paqr4a
PRF1_hcH
LOC100001051
IC_007130.7#0(+)
5,161,930
5,307,449
stx1b
PRF1_hcA
si:dkeyp-113d7.10
V_018394534.1(+)
19,655
67,079
paqr4a
PRF1_hcF
LOC100001051
V_018395274.1(+)
mpeg1.3
PRF1_hcL
mpeg1-like
mpeg1.1
IC_007130.7#1(-)
s100v2
PRF1_hcE
PRF1_hcD
PRF1_hcC
PRF1_hcB
thap7
NC_007119.7(+)
mpeg1.3
PRF1_hcJ
mpeg1-like
PRF1_hcK
mpeg1.1

Cyprinus_carpio

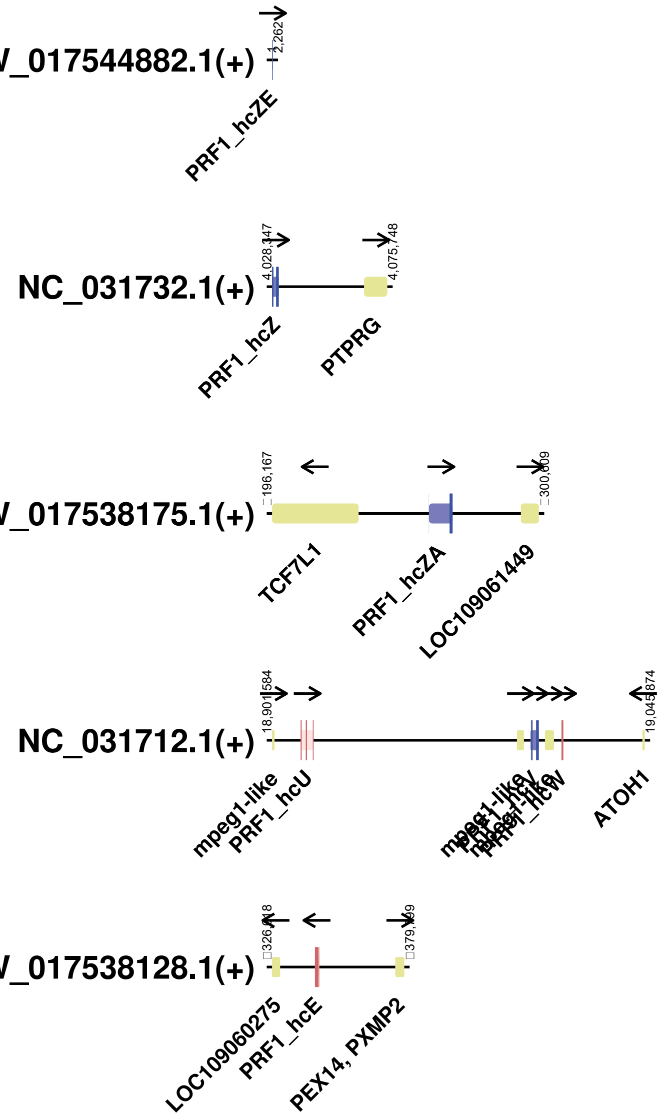

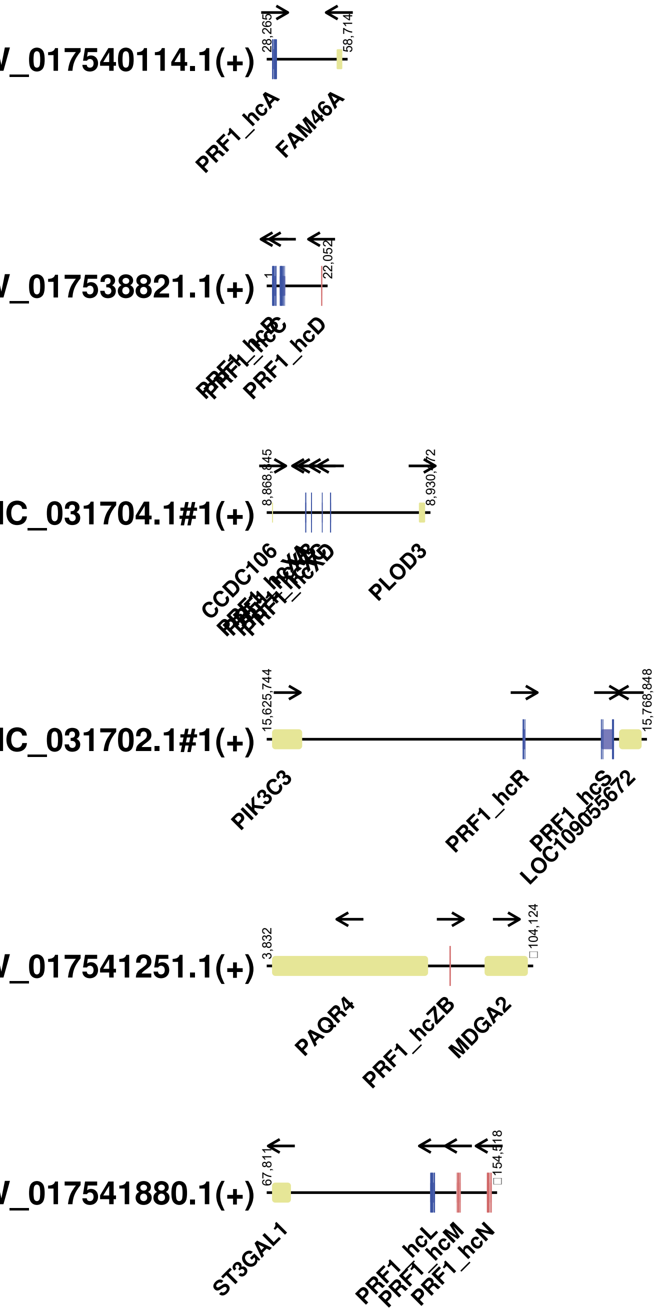

V_017540114.1(+)
28,265
58,714
PRF1_hcA
FAM46A
V_017538821.1(+)
22,052
PRF1_hcD
IC_031704.1#1(+)
8,868,845
CCDC106
PLOD3
IC_031702.1#1(+)
15,625,744
15,768,848
PIK3C3
PRF1_hcR
PRF1_hcS
V_017541251.1(+)
3,832
104,124
PAQR4
PRF1_hcZB
MDGA2
V_017541880.1(+)
67,811
ST3GAL1
PRF1_hcL
PRF1_hcM
PRF1_hcN

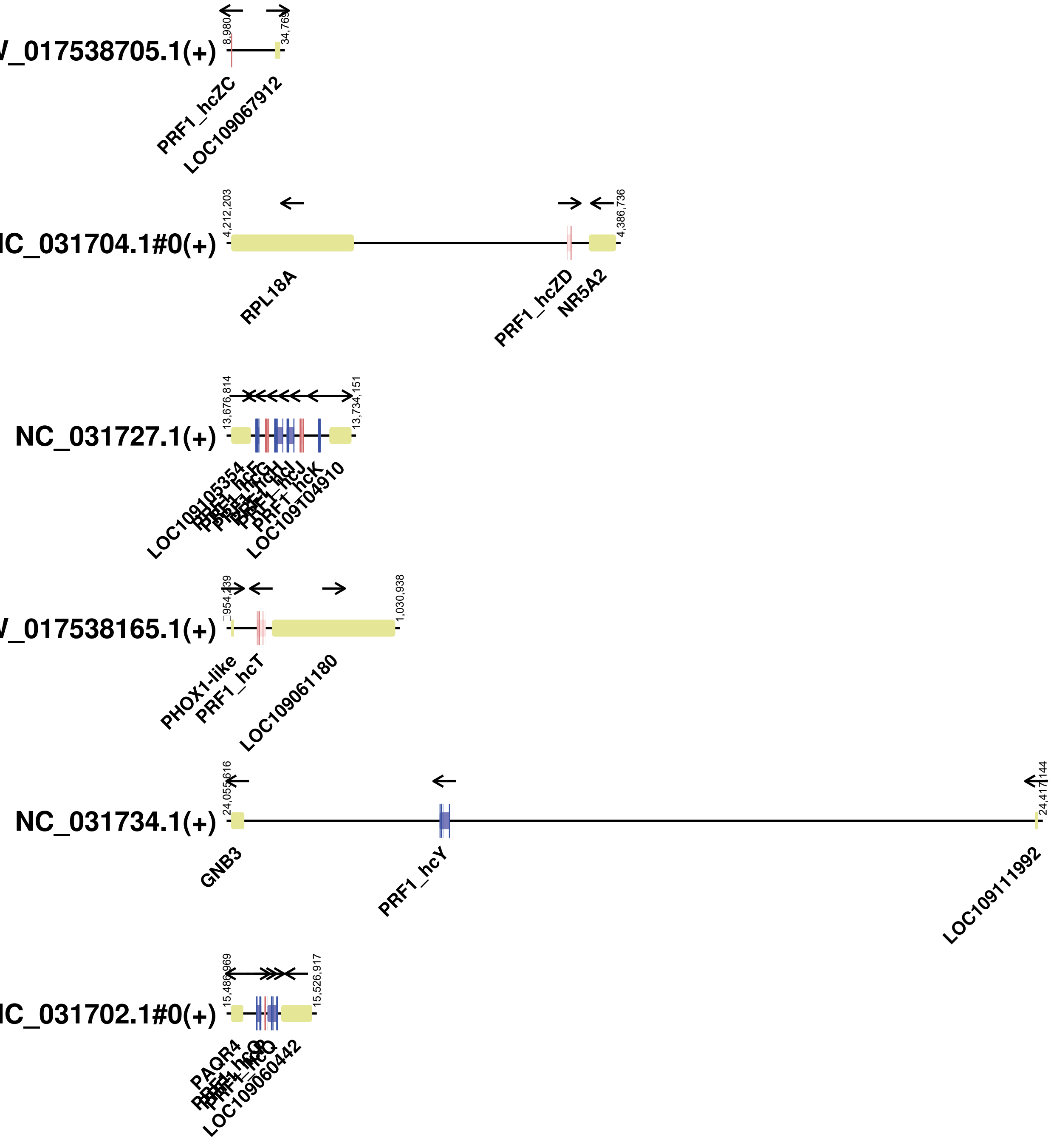

8,980
34,769
PRF1_hcZC
LOC109067912
NC_031704.1#0(+)
4,212,203
4,386,736
RPL18A
PRF1_hcZD
NR5A2
NC_031727.1(+)
13,676,814
13,734,151
LOC109105354
LOC109104910
954,139
1,030,938
PHOX1-like
PRF1_hcT
LOC109061180
NC_031734.1(+)
GNB3
PRF1_hcY
LOC109111992
NC_031702.1#0(+)
15,486,969
15,526,917
PAQR4
LOC109060442

Cynoglossus_semilaevis

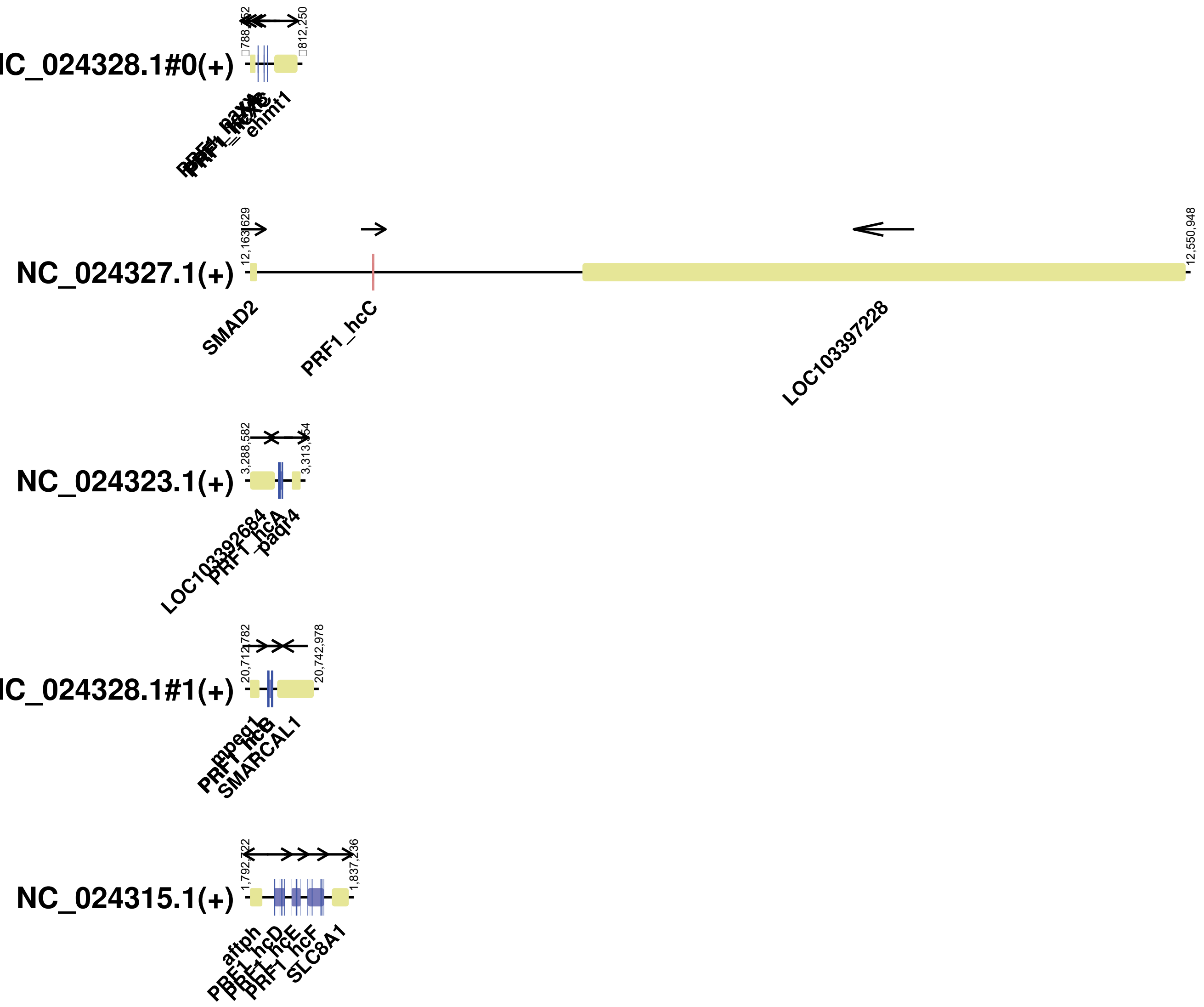

Clupea_harengus

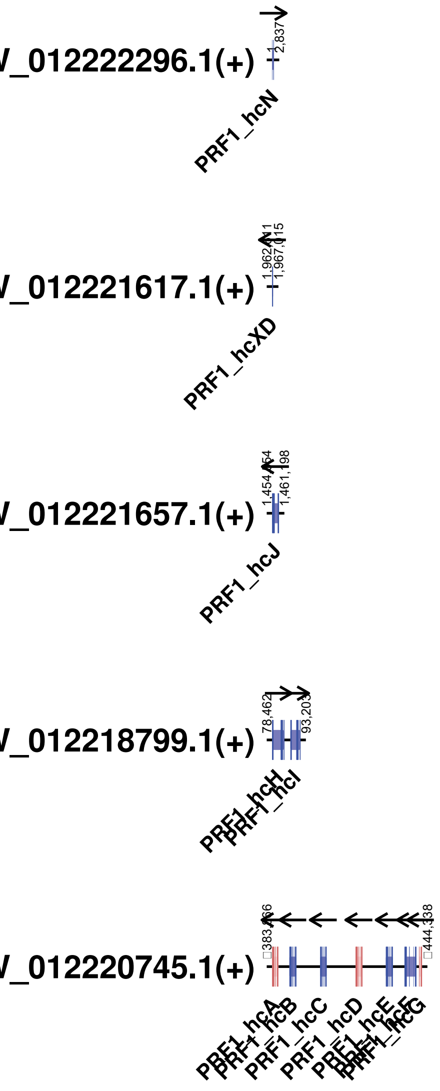

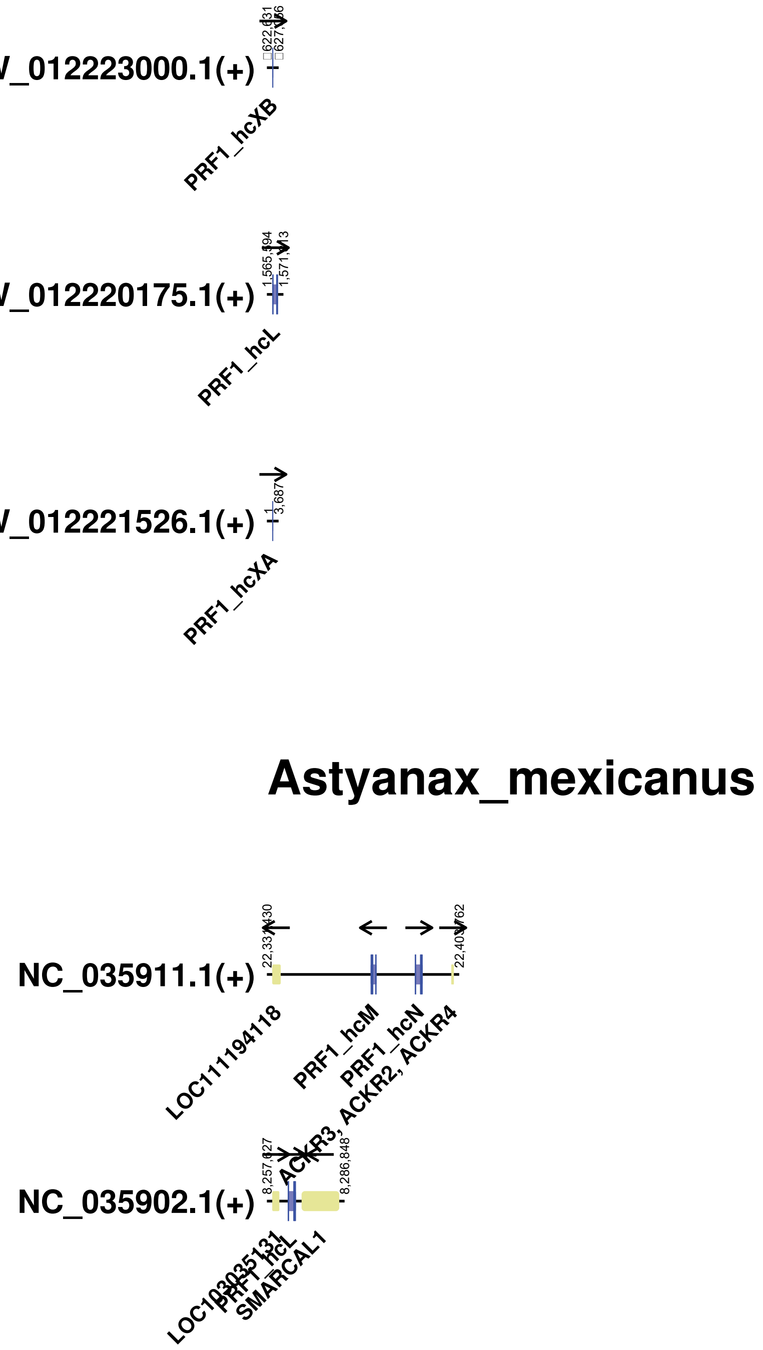

_012223000.1(+)
PRF1_hcXB
_012220175.1(+)
PRF1_hcL
_012221526.1(+)
PRF1_hcXA
Astyanax_mexicanus
NC_035911.1(+)
LOC111194118
PRF1_hcM
PRF1_hcN
ACKR2, ACKR4
NC_035902.1(+)
ACKR3
8,257,027
8,286,848
SMARCAL1

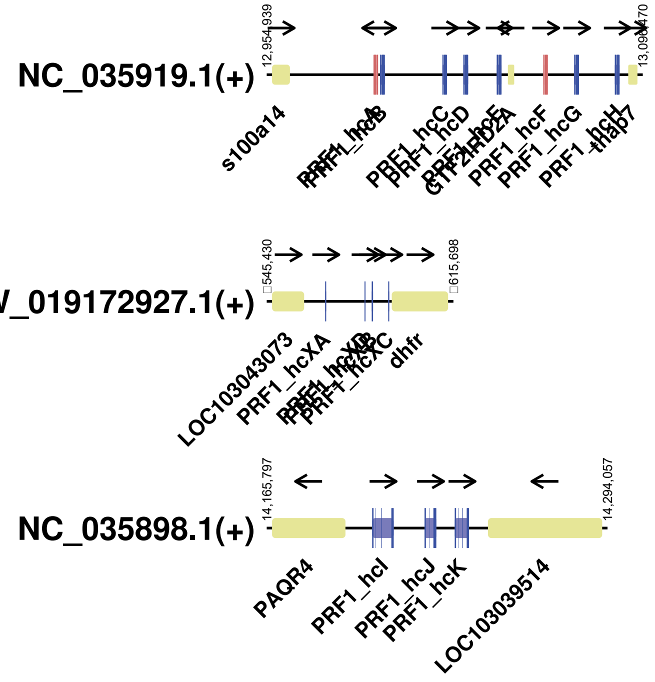

Anguilla_rostrata

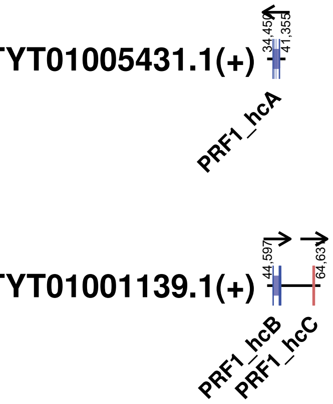

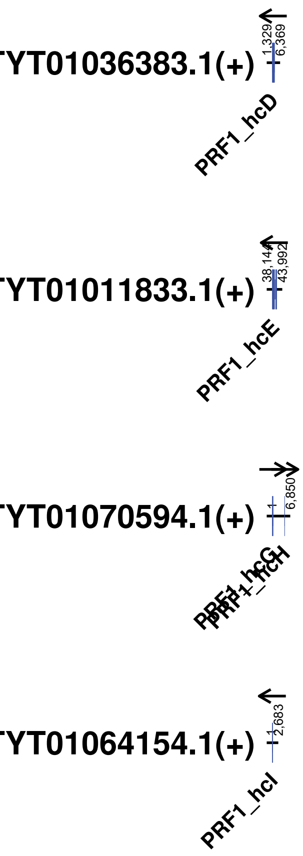

YT01036383.1(+)
1,329
6,369
PRF1_hcD
YT01011833.1(+)
38,144
43,992
PRF1_hcE
YT01070594.1(+)
1
6,850
PRF1_hcG
PRF1_hcH
YT01064154.1(+)
1
2,683
PRF1_hcI
