# Supplementary material for: Frequent birth-and-death events throughout perforin-1 evolution
Source: BMC Evol Biol. 2020 Oct 19;20:135. doi: 10.1186/s12862-020-01698-1 (PMC7574235; doi:10.1186/s12862-020-01698-1)
Supplement: Supplementary file 9 — Additional file 9 Sequence read archive entries used to study bird PRF1 expression [file 12862_2020_1698_MOESM9_ESM.pdf]

Experiment Accession	Organism Name	Instrument	Submitter	Study Accession
DRX026373	Taeniopygia guttata	Illumina HiSeq 2500	HOKUDAI	DRP004407
SRX1282775	Taeniopygia guttata	Illumina HiSeq 2000	Academia Sinice	SRP064028
SRX1282774	Taeniopygia guttata	Illumina HiSeq 2000	Academia Sinice	SRP064028
SRX1282773	Taeniopygia guttata	Illumina HiSeq 2000	Academia Sinice	SRP064028
DRX081219	Taeniopygia guttata	Illumina HiSeq 2500	HOKUDAI	DRP004407
SRX3733415	Taeniopygia guttata	Illumina HiSeq 2500	Institute of Molecular Biology	SRP133266
DRX081220	Taeniopygia guttata	Illumina HiSeq 2500	HOKUDAI	DRP004407
SRX6431682	Taeniopygia castanotis	Illumina HiSeq X Ten	University of Granada	SRP214279
SRX3733408	Taeniopygia guttata	Illumina HiSeq 2500	Institute of Molecular Biology	SRP133266
SRX6431685	Taeniopygia castanotis	Illumina HiSeq X Ten	University of Granada	SRP214279
SRX3733409	Taeniopygia guttata	Illumina HiSeq 2500	Institute of Molecular Biology	SRP133266
SRX3733414	Taeniopygia guttata	Illumina HiSeq 2500	Institute of Molecular Biology	SRP133266
DRX081223	Taeniopygia guttata	Illumina HiSeq 2500	HOKUDAI	DRP004407
DRX081221	Taeniopygia guttata	Illumina HiSeq 2500	HOKUDAI	DRP004407
DRX081222	Taeniopygia guttata	Illumina HiSeq 2500	HOKUDAI	DRP004407
SRX6431684	Taeniopygia castanotis	Illumina HiSeq X Ten	University of Granada	SRP214279
DRX026400	Taeniopygia guttata	Illumina HiSeq 2500	HOKUDAI	DRP004407
SRX2334143	Taeniopygia guttata	Illumina HiSeq 2500	East Carolina University	SRP092791
SRX6431683	Taeniopygia castanotis	Illumina HiSeq X Ten	University of Granada	SRP214279
SRX2334144	Taeniopygia guttata	Illumina HiSeq 2500	East Carolina University	SRP092791
SRX2334148	Taeniopygia guttata	Illumina HiSeq 2500	East Carolina University	SRP092791
SRX5353351	Taeniopygia guttata	Sequel	G10K	SRP184741
SRX3846484	bird metagenome	Illumina MiSeq	American University	SRP136445
SRX2334149	Taeniopygia guttata	Illumina HiSeq 2500	East Carolina University	SRP092791
DRX026394	Taeniopygia guttata	Illumina HiSeq 2500	HOKUDAI	DRP004407
SRX2334142	Taeniopygia guttata	Illumina HiSeq 2500	East Carolina University	SRP092791
SRX3846485	bird metagenome	Illumina MiSeq	American University	SRP136445
SRX2334145	Taeniopygia guttata	Illumina HiSeq 2500	East Carolina University	SRP092791
SRX2334147	Taeniopygia guttata	Illumina HiSeq 2500	East Carolina University	SRP092791
SRX2334141	Taeniopygia guttata	Illumina HiSeq 2500	East Carolina University	SRP092791
DRX026390	Taeniopygia guttata	Illumina HiSeq 2500	HOKUDAI	DRP004407
SRX1616452	Taeniopygia guttata	Illumina HiSeq 2000	East Carolina University	SRP071222
DRX026381	Taeniopygia guttata	Illumina HiSeq 2500	HOKUDAI	DRP004407
SRX5353347	Taeniopygia guttata	Sequel	G10K	SRP184741
DRX026386	Taeniopygia guttata	Illumina HiSeq 2500	HOKUDAI	DRP004407
DRX026435	Taeniopygia guttata	Illumina HiSeq 2500	HOKUDAI	DRP004407
SRX1616455	Taeniopygia guttata	Illumina HiSeq 2000	East Carolina University	SRP071222
SRX2334146	Taeniopygia guttata	Illumina HiSeq 2500	East Carolina University	SRP092791
SRX1616463	Taeniopygia guttata	Illumina HiSeq 2000	East Carolina University	SRP071222
SRX1616454	Taeniopygia guttata	Illumina HiSeq 2000	East Carolina University	SRP071222
SRX1616439	Taeniopygia guttata	Illumina HiSeq 2000	East Carolina University	SRP071222
SRX1616443	Taeniopygia guttata	Illumina HiSeq 2000	East Carolina University	SRP071222
SRX1616449	Taeniopygia guttata	Illumina HiSeq 2000	East Carolina University	SRP071222
DRX026377	Taeniopygia guttata	Illumina HiSeq 2500	HOKUDAI	DRP004407
SRX1205425	Taeniopygia guttata	Illumina HiSeq 2000	East Carolina University	SRP063457
SRX1616437	Taeniopygia guttata	Illumina HiSeq 2000	East Carolina University	SRP071222
SRX1616446	Taeniopygia guttata	Illumina HiSeq 2000	East Carolina University	SRP071222
DRX026431	Taeniopygia guttata	Illumina HiSeq 2500	HOKUDAI	DRP004407
DRX026423	Taeniopygia guttata	Illumina HiSeq 2500	HOKUDAI	DRP004407
SRX1205094	Taeniopygia guttata	Illumina HiSeq 2000	East Carolina University	SRP063457
SRX1725523	Taeniopygia guttata	Illumina HiSeq 2000	East Carolina University	SRP049460
DRX026427	Taeniopygia guttata	Illumina HiSeq 2500	HOKUDAI	DRP004407
SRX5353353	Taeniopygia guttata	Sequel	G10K	SRP184741
SRX5491157	Taeniopygia guttata	NextSeq 500	G10K	SRP184741
SRX1205088	Taeniopygia guttata	Illumina HiSeq 2000	East Carolina University	SRP063457
DRX026419	Taeniopygia guttata	Illumina HiSeq 2500	HOKUDAI	DRP004407
SRX1725470	Taeniopygia guttata	Illumina HiSeq 2000	East Carolina University	SRP049460
SRX5491151	Taeniopygia guttata	NextSeq 500	G10K	SRP184741
SRX5353352	Taeniopygia guttata	NextSeq 500	G10K	SRP184741
DRX026404	Taeniopygia guttata	Illumina HiSeq 2500	HOKUDAI	DRP004407
SRX1725529	Taeniopygia guttata	Illumina HiSeq 2000	East Carolina University	SRP049460
SRX1725471	Taeniopygia guttata	Illumina HiSeq 2000	East Carolina University	SRP049460
SRX5491155	Taeniopygia guttata	NextSeq 500	G10K	SRP184741
DRX176212	Taeniopygia guttata	Illumina HiSeq 2500	HOKUDAI	DRP005406
SRX5491149	Taeniopygia guttata	NextSeq 500	G10K	SRP184741
SRX5353344	Taeniopygia guttata	NextSeq 500	G10K	SRP184741
SRX1725524	Taeniopygia guttata	Illumina HiSeq 2000	East Carolina University	SRP049460
SRX5491153	Taeniopygia guttata	NextSeq 500	G10K	SRP184741
SRX5353350	Taeniopygia guttata	NextSeq 500	G10K	SRP184741
DRX176211	Taeniopygia guttata	Illumina HiSeq 2500	HOKUDAI	DRP005406

Experiment Accession	Organism Name	Instrument	Submitter	Study Accession
DRX176210	Taeniopygia guttata	Illumina HiSeq 2500	HOKUDAI	DRP005406
DRX026415	Taeniopygia guttata	Illumina HiSeq 2500	HOKUDAI	DRP004407
DRX176213	Taeniopygia guttata	Illumina HiSeq 2500	HOKUDAI	DRP005406
SRX1742583	Taeniopygia guttata	Illumina HiSeq 2000	East Carolina University	SRP049460
DRX176209	Taeniopygia guttata	Illumina HiSeq 2500	HOKUDAI	DRP005406
DRX176208	Taeniopygia guttata	Illumina HiSeq 2500	HOKUDAI	DRP005406
SRX493921	Taeniopygia guttata	Illumina Genome Analyzer	East Carolina University	SRP040242
SRX493920	Taeniopygia guttata	Illumina Genome Analyzer	East Carolina University	SRP040242
SRX493922	Taeniopygia guttata	Illumina Genome Analyzer	East Carolina University	SRP040242
SRX5353349	Taeniopygia guttata	Sequel	G10K	SRP184741
SRX5353345	Taeniopygia guttata	Sequel	G10K	SRP184741
SRX738987	Taeniopygia guttata	Illumina HiSeq 2500	Johannes Gutenberg University Mainz	SRP049198
DRX081527	Taeniopygia guttata	Illumina HiSeq 2500	HOKUDAI	DRP004407
ERX2785253	Taeniopygia guttata	Illumina HiSeq 2500	QUEEN MARY UNIVERSITY OF LONDON	ERP110251
ERX2785257	Taeniopygia guttata	Illumina HiSeq 2500	QUEEN MARY UNIVERSITY OF LONDON	ERP110251
ERX2785250	Taeniopygia guttata	Illumina HiSeq 2500	QUEEN MARY UNIVERSITY OF LONDON	ERP110251
ERX2785251	Taeniopygia guttata	Illumina HiSeq 2500	QUEEN MARY UNIVERSITY OF LONDON	ERP110251
ERX2785252	Taeniopygia guttata	Illumina HiSeq 2500	QUEEN MARY UNIVERSITY OF LONDON	ERP110251
ERX2785259	Taeniopygia guttata	Illumina HiSeq 2500	QUEEN MARY UNIVERSITY OF LONDON	ERP110251
ERX2785256	Taeniopygia guttata	Illumina HiSeq 2500	QUEEN MARY UNIVERSITY OF LONDON	ERP110251
ERX2785258	Taeniopygia guttata	Illumina HiSeq 2500	QUEEN MARY UNIVERSITY OF LONDON	ERP110251
ERX2785362	Taeniopygia guttata	Illumina HiSeq 2500	QUEEN MARY UNIVERSITY OF LONDON	ERP110251
ERX2785260	Taeniopygia guttata	Illumina HiSeq 2500	QUEEN MARY UNIVERSITY OF LONDON	ERP110251
ERX2785368	Taeniopygia guttata	Illumina HiSeq 2500	QUEEN MARY UNIVERSITY OF LONDON	ERP110251
ERX2741529	Taeniopygia guttata	Illumina HiSeq 2500	QUEEN MARY UNIVERSITY OF LONDON	ERP110251
ERX2785364	Taeniopygia guttata	Illumina HiSeq 2500	QUEEN MARY UNIVERSITY OF LONDON	ERP110251
ERX2785254	Taeniopygia guttata	Illumina HiSeq 2500	QUEEN MARY UNIVERSITY OF LONDON	ERP110251
ERX2785412	Taeniopygia guttata	Illumina HiSeq 2500	QUEEN MARY UNIVERSITY OF LONDON	ERP110251
ERX2785358	Taeniopygia guttata	Illumina HiSeq 2500	QUEEN MARY UNIVERSITY OF LONDON	ERP110251
ERX2741531	Taeniopygia guttata	Illumina HiSeq 2500	QUEEN MARY UNIVERSITY OF LONDON	ERP110251
ERX2785385	Taeniopygia guttata	Illumina HiSeq 2500	QUEEN MARY UNIVERSITY OF LONDON	ERP110251
ERX2785275	Taeniopygia guttata	Illumina HiSeq 2500	QUEEN MARY UNIVERSITY OF LONDON	ERP110251
ERX2785262	Taeniopygia guttata	Illumina HiSeq 2500	QUEEN MARY UNIVERSITY OF LONDON	ERP110251
ERX2785277	Taeniopygia guttata	Illumina HiSeq 2500	QUEEN MARY UNIVERSITY OF LONDON	ERP110251
ERX2785431	Taeniopygia guttata	Illumina HiSeq 2500	QUEEN MARY UNIVERSITY OF LONDON	ERP110251
ERX2785430	Taeniopygia guttata	Illumina HiSeq 2500	QUEEN MARY UNIVERSITY OF LONDON	ERP110251
ERX2785433	Taeniopygia guttata	Illumina HiSeq 2500	QUEEN MARY UNIVERSITY OF LONDON	ERP110251
ERX2785238	Taeniopygia guttata	Illumina HiSeq 2500	QUEEN MARY UNIVERSITY OF LONDON	ERP110251
ERX2785357	Taeniopygia guttata	Illumina HiSeq 2500	QUEEN MARY UNIVERSITY OF LONDON	ERP110251
ERX2785370	Taeniopygia guttata	Illumina HiSeq 2500	QUEEN MARY UNIVERSITY OF LONDON	ERP110251
ERX2785389	Taeniopygia guttata	Illumina HiSeq 2500	QUEEN MARY UNIVERSITY OF LONDON	ERP110251
ERX2785328	Taeniopygia guttata	Illumina HiSeq 2500	QUEEN MARY UNIVERSITY OF LONDON	ERP110251
ERX2785310	Taeniopygia guttata	Illumina HiSeq 2500	QUEEN MARY UNIVERSITY OF LONDON	ERP110251
ERX2785321	Taeniopygia guttata	Illumina HiSeq 2500	QUEEN MARY UNIVERSITY OF LONDON	ERP110251
ERX2785306	Taeniopygia guttata	Illumina HiSeq 2500	QUEEN MARY UNIVERSITY OF LONDON	ERP110251
ERX2785304	Taeniopygia guttata	Illumina HiSeq 2500	QUEEN MARY UNIVERSITY OF LONDON	ERP110251
ERX2785383	Taeniopygia guttata	Illumina HiSeq 2500	QUEEN MARY UNIVERSITY OF LONDON	ERP110251
ERX2785415	Taeniopygia guttata	Illumina HiSeq 2500	QUEEN MARY UNIVERSITY OF LONDON	ERP110251
ERX2785240	Taeniopygia guttata	Illumina HiSeq 2500	QUEEN MARY UNIVERSITY OF LONDON	ERP110251
ERX2785384	Taeniopygia guttata	Illumina HiSeq 2500	QUEEN MARY UNIVERSITY OF LONDON	ERP110251
ERX2785410	Taeniopygia guttata	Illumina HiSeq 2500	QUEEN MARY UNIVERSITY OF LONDON	ERP110251
ERX2785265	Taeniopygia guttata	Illumina HiSeq 2500	QUEEN MARY UNIVERSITY OF LONDON	ERP110251
ERX2785395	Taeniopygia guttata	Illumina HiSeq 2500	QUEEN MARY UNIVERSITY OF LONDON	ERP110251
ERX2785434	Taeniopygia guttata	Illumina HiSeq 2500	QUEEN MARY UNIVERSITY OF LONDON	ERP110251
ERX2785323	Taeniopygia guttata	Illumina HiSeq 2500	QUEEN MARY UNIVERSITY OF LONDON	ERP110251
ERX2785388	Taeniopygia guttata	Illumina HiSeq 2500	QUEEN MARY UNIVERSITY OF LONDON	ERP110251
ERX2785381	Taeniopygia guttata	Illumina HiSeq 2500	QUEEN MARY UNIVERSITY OF LONDON	ERP110251
ERX2785324	Taeniopygia guttata	Illumina HiSeq 2500	QUEEN MARY UNIVERSITY OF LONDON	ERP110251
ERX2785278	Taeniopygia guttata	Illumina HiSeq 2500	QUEEN MARY UNIVERSITY OF LONDON	ERP110251
ERX2785308	Taeniopygia guttata	Illumina HiSeq 2500	QUEEN MARY UNIVERSITY OF LONDON	ERP110251
ERX2785369	Taeniopygia guttata	Illumina HiSeq 2500	QUEEN MARY UNIVERSITY OF LONDON	ERP110251
ERX2785414	Taeniopygia guttata	Illumina HiSeq 2500	QUEEN MARY UNIVERSITY OF LONDON	ERP110251
ERX2785379	Taeniopygia guttata	Illumina HiSeq 2500	QUEEN MARY UNIVERSITY OF LONDON	ERP110251
ERX2785411	Taeniopygia guttata	Illumina HiSeq 2500	QUEEN MARY UNIVERSITY OF LONDON	ERP110251
ERX2785394	Taeniopygia guttata	Illumina HiSeq 2500	QUEEN MARY UNIVERSITY OF LONDON	ERP110251
ERX2785374	Taeniopygia guttata	Illumina HiSeq 2500	QUEEN MARY UNIVERSITY OF LONDON	ERP110251
ERX2785305	Taeniopygia guttata	Illumina HiSeq 2500	QUEEN MARY UNIVERSITY OF LONDON	ERP110251
ERX2785243	Taeniopygia guttata	Illumina HiSeq 2500	QUEEN MARY UNIVERSITY OF LONDON	ERP110251
ERX2785309	Taeniopygia guttata	Illumina HiSeq 2500	QUEEN MARY UNIVERSITY OF LONDON	ERP110251
ERX2785390	Taeniopygia guttata	Illumina HiSeq 2500	QUEEN MARY UNIVERSITY OF LONDON	ERP110251

Experiment Accession	Organism Name	Instrument	Submitter	Study Accession
ERX2785386	Taeniopygia guttata	Illumina HiSeq 2500	QUEEN MARY UNIVERSITY OF LONDON	ERP110251
ERX2785247	Taeniopygia guttata	Illumina HiSeq 2500	QUEEN MARY UNIVERSITY OF LONDON	ERP110251
ERX2785406	Taeniopygia guttata	Illumina HiSeq 2500	QUEEN MARY UNIVERSITY OF LONDON	ERP110251
ERX2785391	Taeniopygia guttata	Illumina HiSeq 2500	QUEEN MARY UNIVERSITY OF LONDON	ERP110251
ERX2785405	Taeniopygia guttata	Illumina HiSeq 2500	QUEEN MARY UNIVERSITY OF LONDON	ERP110251
ERX2785361	Taeniopygia guttata	Illumina HiSeq 2500	QUEEN MARY UNIVERSITY OF LONDON	ERP110251
ERX2785416	Taeniopygia guttata	Illumina HiSeq 2500	QUEEN MARY UNIVERSITY OF LONDON	ERP110251
ERX2785373	Taeniopygia guttata	Illumina HiSeq 2500	QUEEN MARY UNIVERSITY OF LONDON	ERP110251
ERX2785375	Taeniopygia guttata	Illumina HiSeq 2500	QUEEN MARY UNIVERSITY OF LONDON	ERP110251
ERX2785360	Taeniopygia guttata	Illumina HiSeq 2500	QUEEN MARY UNIVERSITY OF LONDON	ERP110251
ERX2785359	Taeniopygia guttata	Illumina HiSeq 2500	QUEEN MARY UNIVERSITY OF LONDON	ERP110251
ERX2785363	Taeniopygia guttata	Illumina HiSeq 2500	QUEEN MARY UNIVERSITY OF LONDON	ERP110251
ERX2785329	Taeniopygia guttata	Illumina HiSeq 2500	QUEEN MARY UNIVERSITY OF LONDON	ERP110251
ERX2785248	Taeniopygia guttata	Illumina HiSeq 2500	QUEEN MARY UNIVERSITY OF LONDON	ERP110251
ERX2785376	Taeniopygia guttata	Illumina HiSeq 2500	QUEEN MARY UNIVERSITY OF LONDON	ERP110251
ERX2785371	Taeniopygia guttata	Illumina HiSeq 2500	QUEEN MARY UNIVERSITY OF LONDON	ERP110251
ERX2785372	Taeniopygia guttata	Illumina HiSeq 2500	QUEEN MARY UNIVERSITY OF LONDON	ERP110251
ERX2785419	Taeniopygia guttata	Illumina HiSeq 2500	QUEEN MARY UNIVERSITY OF LONDON	ERP110251
ERX2785320	Taeniopygia guttata	Illumina HiSeq 2500	QUEEN MARY UNIVERSITY OF LONDON	ERP110251
ERX2785404	Taeniopygia guttata	Illumina HiSeq 2500	QUEEN MARY UNIVERSITY OF LONDON	ERP110251
ERX2785436	Taeniopygia guttata	Illumina HiSeq 2500	QUEEN MARY UNIVERSITY OF LONDON	ERP110251
ERX2785288	Taeniopygia guttata	Illumina HiSeq 2500	QUEEN MARY UNIVERSITY OF LONDON	ERP110251
ERX2785269	Taeniopygia guttata	Illumina HiSeq 2500	QUEEN MARY UNIVERSITY OF LONDON	ERP110251
ERX2785343	Taeniopygia guttata	Illumina HiSeq 2500	QUEEN MARY UNIVERSITY OF LONDON	ERP110251
ERX2785242	Taeniopygia guttata	Illumina HiSeq 2500	QUEEN MARY UNIVERSITY OF LONDON	ERP110251
ERX2785331	Taeniopygia guttata	Illumina HiSeq 2500	QUEEN MARY UNIVERSITY OF LONDON	ERP110251
ERX2785313	Taeniopygia guttata	Illumina HiSeq 2500	QUEEN MARY UNIVERSITY OF LONDON	ERP110251
ERX2785287	Taeniopygia guttata	Illumina HiSeq 2500	QUEEN MARY UNIVERSITY OF LONDON	ERP110251
ERX2785317	Taeniopygia guttata	Illumina HiSeq 2500	QUEEN MARY UNIVERSITY OF LONDON	ERP110251
ERX2785272	Taeniopygia guttata	Illumina HiSeq 2500	QUEEN MARY UNIVERSITY OF LONDON	ERP110251
ERX2785441	Taeniopygia guttata	Illumina HiSeq 2500	QUEEN MARY UNIVERSITY OF LONDON	ERP110251
ERX2785440	Taeniopygia guttata	Illumina HiSeq 2500	QUEEN MARY UNIVERSITY OF LONDON	ERP110251
ERX2785437	Taeniopygia guttata	Illumina HiSeq 2500	QUEEN MARY UNIVERSITY OF LONDON	ERP110251
ERX2785445	Taeniopygia guttata	Illumina HiSeq 2500	QUEEN MARY UNIVERSITY OF LONDON	ERP110251
ERX2785443	Taeniopygia guttata	Illumina HiSeq 2500	QUEEN MARY UNIVERSITY OF LONDON	ERP110251
ERX2785438	Taeniopygia guttata	Illumina HiSeq 2500	QUEEN MARY UNIVERSITY OF LONDON	ERP110251
ERX2785442	Taeniopygia guttata	Illumina HiSeq 2500	QUEEN MARY UNIVERSITY OF LONDON	ERP110251
ERX2785444	Taeniopygia guttata	Illumina HiSeq 2500	QUEEN MARY UNIVERSITY OF LONDON	ERP110251
ERX2785446	Taeniopygia guttata	Illumina HiSeq 2500	QUEEN MARY UNIVERSITY OF LONDON	ERP110251
ERX2785439	Taeniopygia guttata	Illumina HiSeq 2500	QUEEN MARY UNIVERSITY OF LONDON	ERP110251
SRX5491152	Taeniopygia guttata	Sequel	G10K	SRP184741
SRX5491150	Taeniopygia guttata	Sequel	G10K	SRP184741
SRX5491156	Taeniopygia guttata	Sequel	G10K	SRP184741
SRX5491148	Taeniopygia guttata	Sequel	G10K	SRP184741
SRX5491154	Taeniopygia guttata	Sequel	G10K	SRP184741
SRX1299468				
SRX1299467				
SRX1299466				
SRX1299465				
SRX1299464				
SRX1299463				
SRX1299462				
SRX1299461				
SRX1299460				
SRX1299459				
SRX1299458				
SRX1299457				
SRX1299456				
SRX1299455				
SRX1299454				
SRX1299453				
SRX1299452				
SRX1299451				
SRX1299450				
SRX1299449				
SRX1299448				
SRX1299447				
SRX1299446				
SRX1299445				
