# Supplementary material for: Frequent birth-and-death events throughout perforin-1 evolution
Source: BMC Evol Biol. 2020 Oct 19;20:135. doi: 10.1186/s12862-020-01698-1 (PMC7574235; doi:10.1186/s12862-020-01698-1)
Supplement: Supplementary file 11 — Additional file 11 SignalP prediction of secretion peptides in c2PRF1 products [file 12862_2020_1698_MOESM11_ESM.pdf]

```
{
  "INFO": {
    "failedjobs": 0,
    "size": 41
  },
  "CSV_FILE": "/services/SignalP-5.0/tmp/5E5001DD00004035309DA2C3/output_protein_type.txt",
  "MATURE_FILE": "/services/SignalP-5.0/tmp/5E5001DD00004035309DA2C3/output_mature.fasta",
  "GFF_FILE": "/services/SignalP-5.0/tmp/5E5001DD00004035309DA2C3/output.gff3",
  "ZIP_FILE": "/services/SignalP-5.0/tmp/5E5001DD00004035309DA2C3/output_all_results.zip",
  "SEQUENCES": {
    "astMex2.0_hcXA": {
      "CS_pos": "Cleavage site between pos. 22 and 23: TDA-AV. Probability: 0.9539",
      "Likelihood": [
        0.9968,
        0.0032
      ],
      "Name": "astMex2.0_hcXA",
      "Plot_eps": "/services/SignalP-5.0/tmp/5E5001DD00004035309DA2C3/output_astMex2.0_hcXA_plot.eps",
      "Plot_png": "/services/SignalP-5.0/tmp/5E5001DD00004035309DA2C3/output_astMex2.0_hcXA_plot.png",
      "Plot_txt": "/services/SignalP-5.0/tmp/5E5001DD00004035309DA2C3/output_astMex2.0_hcXA_pred.txt",
      "Prediction": "Signal peptide (Sec/SPI)",
      "Protein_types": [
        "Signal Peptide (Sec/SPI)",
        "Other"
      ]
    },
    "astMex2.0_hcXB": {
      "CS_pos": "Cleavage site between pos. 24 and 25: GDA-VV. Probability: 0.7121",
      "Likelihood": [
        0.9946,
        0.0054
      ],
      "Name": "astMex2.0_hcXB",
      "Plot_eps": "/services/SignalP-5.0/tmp/5E5001DD00004035309DA2C3/output_astMex2.0_hcXB_plot.eps",
      "Plot_png": "/services/SignalP-5.0/tmp/5E5001DD00004035309DA2C3/output_astMex2.0_hcXB_plot.png",
      "Plot_txt": "/services/SignalP-5.0/tmp/5E5001DD00004035309DA2C3/output_astMex2.0_hcXB_pred.txt",
      "Prediction": "Signal peptide (Sec/SPI)",
      "Protein_types": [
        "Signal Peptide (Sec/SPI)",
        "Other"
      ]
    },
    "astMex2.0_hcXC": {
      "CS_pos": "Cleavage site between pos. 20 and 21: GDA-AV. Probability: 0.8785",
      "Likelihood": [
        0.996,
        0.004
      ],
      "Name": "astMex2.0_hcXC",
      "Plot_eps": "/services/SignalP-5.0/tmp/5E5001DD00004035309DA2C3/output_astMex2.0_hcXC_plot.eps",
      "Plot_png": "/services/SignalP-5.0/tmp/5E5001DD00004035309DA2C3/output_astMex2.0_hcXC_plot.png",
      "Plot_txt": "/services/SignalP-5.0/tmp/5E5001DD00004035309DA2C3/output_astMex2.0_hcXC_pred.txt",
      "Prediction": "Signal peptide (Sec/SPI)",
      "Protein_types": [
        "Signal Peptide (Sec/SPI)",
        "Other"
      ]
    },
    "astMex2.0_hcXD": {
      "CS_pos": "Cleavage site between pos. 19 and 20: GDA-GT. Probability: 0.7557",
      "Likelihood": [
        0.9973,
        0.0027
      ],
      "Name": "astMex2.0_hcXD",
      "Plot_eps": "/services/SignalP-5.0/tmp/5E5001DD00004035309DA2C3/output_astMex2.0_hcXD_plot.eps",
      "Plot_png": "/services/SignalP-5.0/tmp/5E5001DD00004035309DA2C3/output_astMex2.0_hcXD_plot.png",
      "Plot_txt": "/services/SignalP-5.0/tmp/5E5001DD00004035309DA2C3/output_astMex2.0_hcXD_pred.txt",
      "Prediction": "Signal peptide (Sec/SPI)",
      "Protein_types": [
        "Signal Peptide (Sec/SPI)",
        "Other"
      ]
    },
    "cluHar1_hcXA": {
      "CS_pos": "Cleavage site between pos. 23 and 24: ATA-DV. Probability: 0.8034",
      "Likelihood": [
        0.9914,
        0.0086
      ],
      "Name": "cluHar1_hcXA",
      "Plot_eps": "/services/SignalP-5.0/tmp/5E5001DD00004035309DA2C3/output_cluHar1_hcXA_plot.eps",
      "Plot_png": "/services/SignalP-5.0/tmp/5E5001DD00004035309DA2C3/output_cluHar1_hcXA_plot.png",
      "Plot_txt": "/services/SignalP-5.0/tmp/5E5001DD00004035309DA2C3/output_cluHar1_hcXA_pred.txt",
      "Prediction": "Signal peptide (Sec/SPI)",
      "Protein_types": [
        "Signal Peptide (Sec/SPI)",
        "Other"
      ]
    },
    "cluHar1_hcXB": {
      "CS_pos": "Cleavage site between pos. 23 and 24: ATA-DV. Probability: 0.8258",
      "Likelihood": [
        0.9928,
        0.0072
      ],
      "Name": "cluHar1_hcXB",
      "Plot_eps": "/services/SignalP-5.0/tmp/5E5001DD00004035309DA2C3/output_cluHar1_hcXB_plot.eps",
      "Plot_png": "/services/SignalP-5.0/tmp/5E5001DD00004035309DA2C3/output_cluHar1_hcXB_plot.png",
```

```
    "Plot_txt": "/services/SignalP-5.0/tmp/5E5001DD00004035309DA2C3/output_cluHar1_hcXB_pred.txt",
    "Prediction": "Signal peptide (Sec/SPI)",
    "Protein_types": [
      "Signal Peptide (Sec/SPI)",
      "Other"
    ]
  },
  "cluHar1_hcXD": {
    "CS_pos": "Cleavage site between pos. 23 and 24: ATA-DV. Probability: 0.7810",
    "Likelihood": [
      0.9921,
      0.0079
    ],
    "Name": "cluHar1_hcXD",
    "Plot_eps": "/services/SignalP-5.0/tmp/5E5001DD00004035309DA2C3/output_cluHar1_hcXD_plot.eps",
    "Plot_png": "/services/SignalP-5.0/tmp/5E5001DD00004035309DA2C3/output_cluHar1_hcXD_plot.png",
    "Plot_txt": "/services/SignalP-5.0/tmp/5E5001DD00004035309DA2C3/output_cluHar1_hcXD_pred.txt",
    "Prediction": "Signal peptide (Sec/SPI)",
    "Protein_types": [
      "Signal Peptide (Sec/SPI)",
      "Other"
    ]
  },
  "cynSem1.0_hcXA": {
    "CS_pos": "Cleavage site between pos. 21 and 22: AKA-QL. Probability: 0.8271",
    "Likelihood": [
      0.9961,
      0.0039
    ],
    "Name": "cynSem1.0_hcXA",
    "Plot_eps": "/services/SignalP-5.0/tmp/5E5001DD00004035309DA2C3/output_cynSem1.0_hcXA_plot.eps",
    "Plot_png": "/services/SignalP-5.0/tmp/5E5001DD00004035309DA2C3/output_cynSem1.0_hcXA_plot.png",
    "Plot_txt": "/services/SignalP-5.0/tmp/5E5001DD00004035309DA2C3/output_cynSem1.0_hcXA_pred.txt",
    "Prediction": "Signal peptide (Sec/SPI)",
    "Protein_types": [
      "Signal Peptide (Sec/SPI)",
      "Other"
    ]
  },
  "cynSem1.0_hcXB": {
    "CS_pos": "Cleavage site between pos. 21 and 22: AKA-QL. Probability: 0.8273",
    "Likelihood": [
      0.9968,
      0.0032
    ],
    "Name": "cynSem1.0_hcXB",
    "Plot_eps": "/services/SignalP-5.0/tmp/5E5001DD00004035309DA2C3/output_cynSem1.0_hcXB_plot.eps",
    "Plot_png": "/services/SignalP-5.0/tmp/5E5001DD00004035309DA2C3/output_cynSem1.0_hcXB_plot.png",
    "Plot_txt": "/services/SignalP-5.0/tmp/5E5001DD00004035309DA2C3/output_cynSem1.0_hcXB_pred.txt",
    "Prediction": "Signal peptide (Sec/SPI)",
    "Protein_types": [
      "Signal Peptide (Sec/SPI)",
      "Other"
    ]
  },
  "cynSem1.0_hcXC": {
    "CS_pos": "Cleavage site between pos. 20 and 21: AAA-QL. Probability: 0.6857",
    "Likelihood": [
      0.9601,
      0.0399
    ],
    "Name": "cynSem1.0_hcXC",
    "Plot_eps": "/services/SignalP-5.0/tmp/5E5001DD00004035309DA2C3/output_cynSem1.0_hcXC_plot.eps",
    "Plot_png": "/services/SignalP-5.0/tmp/5E5001DD00004035309DA2C3/output_cynSem1.0_hcXC_plot.png",
    "Plot_txt": "/services/SignalP-5.0/tmp/5E5001DD00004035309DA2C3/output_cynSem1.0_hcXC_pred.txt",
    "Prediction": "Signal peptide (Sec/SPI)",
    "Protein_types": [
      "Signal Peptide (Sec/SPI)",
      "Other"
    ]
  },
  "cypCar_hcXA": {
    "CS_pos": "Cleavage site between pos. 26 and 27: SSA-AV. Probability: 0.8648",
    "Likelihood": [
      0.9941,
      0.0059
    ],
    "Name": "cypCar_hcXA",
    "Plot_eps": "/services/SignalP-5.0/tmp/5E5001DD00004035309DA2C3/output_cypCar_hcXA_plot.eps",
    "Plot_png": "/services/SignalP-5.0/tmp/5E5001DD00004035309DA2C3/output_cypCar_hcXA_plot.png",
    "Plot_txt": "/services/SignalP-5.0/tmp/5E5001DD00004035309DA2C3/output_cypCar_hcXA_pred.txt",
    "Prediction": "Signal peptide (Sec/SPI)",
    "Protein_types": [
      "Signal Peptide (Sec/SPI)",
      "Other"
    ]
  },
  "cypCar_hcXB": {
    "CS_pos": "",
    "Likelihood": [
      0.0799,
      0.9201
    ],
    "Name": "cypCar_hcXB",
    "Plot_eps": "/services/SignalP-5.0/tmp/5E5001DD00004035309DA2C3/output_cypCar_hcXB_plot.eps",
    "Plot_png": "/services/SignalP-5.0/tmp/5E5001DD00004035309DA2C3/output_cypCar_hcXB_plot.png",
    "Plot_txt": "/services/SignalP-5.0/tmp/5E5001DD00004035309DA2C3/output_cypCar_hcXB_pred.txt",
    "Prediction": "Other",
    "Protein_types": [
```

```
    "Signal Peptide (Sec/SPI)",
    "Other"
  ]
},
"cypCar_hcXC": {
  "CS_pos": "",
  "Likelihood": [
    0.0735,
    0.9265
  ],
  "Name": "cypCar_hcXC",
  "Plot_eps": "/services/SignalP-5.0/tmp/5E5001DD00004035309DA2C3/output_cypCar_hcXC_plot.eps",
  "Plot_png": "/services/SignalP-5.0/tmp/5E5001DD00004035309DA2C3/output_cypCar_hcXC_plot.png",
  "Plot_txt": "/services/SignalP-5.0/tmp/5E5001DD00004035309DA2C3/output_cypCar_hcXC_pred.txt",
  "Prediction": "Other",
  "Protein_types": [
    "Signal Peptide (Sec/SPI)",
    "Other"
  ]
},
"cypCar_hcXD": {
  "CS_pos": "Cleavage site between pos. 26 and 27: ASA-GV. Probability: 0.8776",
  "Likelihood": [
    0.9835,
    0.0165
  ],
  "Name": "cypCar_hcXD",
  "Plot_eps": "/services/SignalP-5.0/tmp/5E5001DD00004035309DA2C3/output_cypCar_hcXD_plot.eps",
  "Plot_png": "/services/SignalP-5.0/tmp/5E5001DD00004035309DA2C3/output_cypCar_hcXD_plot.png",
  "Plot_txt": "/services/SignalP-5.0/tmp/5E5001DD00004035309DA2C3/output_cypCar_hcXD_pred.txt",
  "Prediction": "Signal peptide (Sec/SPI)",
  "Protein_types": [
    "Signal Peptide (Sec/SPI)",
    "Other"
  ]
},
"danRer11_hcXA": {
  "CS_pos": "Cleavage site between pos. 26 and 27: ASA-SV. Probability: 0.8796",
  "Likelihood": [
    0.98,
    0.02
  ],
  "Name": "danRer11_hcXA",
  "Plot_eps": "/services/SignalP-5.0/tmp/5E5001DD00004035309DA2C3/output_danRer11_hcXA_plot.eps",
  "Plot_png": "/services/SignalP-5.0/tmp/5E5001DD00004035309DA2C3/output_danRer11_hcXA_plot.png",
  "Plot_txt": "/services/SignalP-5.0/tmp/5E5001DD00004035309DA2C3/output_danRer11_hcXA_pred.txt",
  "Prediction": "Signal peptide (Sec/SPI)",
  "Protein_types": [
    "Signal Peptide (Sec/SPI)",
    "Other"
  ]
},
"danRer11_hcXB": {
  "CS_pos": "Cleavage site between pos. 26 and 27: AKA-AV. Probability: 0.9341",
  "Likelihood": [
    0.9966,
    0.0034
  ],
  "Name": "danRer11_hcXB",
  "Plot_eps": "/services/SignalP-5.0/tmp/5E5001DD00004035309DA2C3/output_danRer11_hcXB_plot.eps",
  "Plot_png": "/services/SignalP-5.0/tmp/5E5001DD00004035309DA2C3/output_danRer11_hcXB_plot.png",
  "Plot_txt": "/services/SignalP-5.0/tmp/5E5001DD00004035309DA2C3/output_danRer11_hcXB_pred.txt",
  "Prediction": "Signal peptide (Sec/SPI)",
  "Protein_types": [
    "Signal Peptide (Sec/SPI)",
    "Other"
  ]
},
"danRer11_hcXC": {
  "CS_pos": "Cleavage site between pos. 26 and 27: ASA-SV. Probability: 0.8856",
  "Likelihood": [
    0.9917,
    0.0083
  ],
  "Name": "danRer11_hcXC",
  "Plot_eps": "/services/SignalP-5.0/tmp/5E5001DD00004035309DA2C3/output_danRer11_hcXC_plot.eps",
  "Plot_png": "/services/SignalP-5.0/tmp/5E5001DD00004035309DA2C3/output_danRer11_hcXC_plot.png",
  "Plot_txt": "/services/SignalP-5.0/tmp/5E5001DD00004035309DA2C3/output_danRer11_hcXC_pred.txt",
  "Prediction": "Signal peptide (Sec/SPI)",
  "Protein_types": [
    "Signal Peptide (Sec/SPI)",
    "Other"
  ]
},
"danRer11_hcXD": {
  "CS_pos": "Cleavage site between pos. 26 and 27: GKA-DV. Probability: 0.9261",
  "Likelihood": [
    0.9713,
    0.0287
  ],
  "Name": "danRer11_hcXD",
  "Plot_eps": "/services/SignalP-5.0/tmp/5E5001DD00004035309DA2C3/output_danRer11_hcXD_plot.eps",
  "Plot_png": "/services/SignalP-5.0/tmp/5E5001DD00004035309DA2C3/output_danRer11_hcXD_plot.png",
  "Plot_txt": "/services/SignalP-5.0/tmp/5E5001DD00004035309DA2C3/output_danRer11_hcXD_pred.txt",
  "Prediction": "Signal peptide (Sec/SPI)",
  "Protein_types": [
    "Signal Peptide (Sec/SPI)",
    "Other"
  ]
```

```
  },
  "danRer11_hcXE": {
    "CS_pos": "Cleavage site between pos. 26 and 27: ASA-SV. Probability: 0.8796",
    "Likelihood": [
      0.98,
      0.02
    ],
    "Name": "danRer11_hcXE",
    "Plot_eps": "/services/SignalP-5.0/tmp/5E5001DD00004035309DA2C3/output_danRer11_hcXE_plot.eps",
    "Plot_png": "/services/SignalP-5.0/tmp/5E5001DD00004035309DA2C3/output_danRer11_hcXE_plot.png",
    "Plot_txt": "/services/SignalP-5.0/tmp/5E5001DD00004035309DA2C3/output_danRer11_hcXE_pred.txt",
    "Prediction": "Signal peptide (Sec/SPI)",
    "Protein_types": [
      "Signal Peptide (Sec/SPI)",
      "Other"
    ]
  },
  "danRer11_hcXF": {
    "CS_pos": "Cleavage site between pos. 26 and 27: AKA-AV. Probability: 0.8523",
    "Likelihood": [
      0.9982,
      0.0018
    ],
    "Name": "danRer11_hcXF",
    "Plot_eps": "/services/SignalP-5.0/tmp/5E5001DD00004035309DA2C3/output_danRer11_hcXF_plot.eps",
    "Plot_png": "/services/SignalP-5.0/tmp/5E5001DD00004035309DA2C3/output_danRer11_hcXF_plot.png",
    "Plot_txt": "/services/SignalP-5.0/tmp/5E5001DD00004035309DA2C3/output_danRer11_hcXF_pred.txt",
    "Prediction": "Signal peptide (Sec/SPI)",
    "Protein_types": [
      "Signal Peptide (Sec/SPI)",
      "Other"
    ]
  },
  "danRer11_hcXG": {
    "CS_pos": "Cleavage site between pos. 26 and 27: ASA-SV. Probability: 0.8856",
    "Likelihood": [
      0.9917,
      0.0083
    ],
    "Name": "danRer11_hcXG",
    "Plot_eps": "/services/SignalP-5.0/tmp/5E5001DD00004035309DA2C3/output_danRer11_hcXG_plot.eps",
    "Plot_png": "/services/SignalP-5.0/tmp/5E5001DD00004035309DA2C3/output_danRer11_hcXG_plot.png",
    "Plot_txt": "/services/SignalP-5.0/tmp/5E5001DD00004035309DA2C3/output_danRer11_hcXG_pred.txt",
    "Prediction": "Signal peptide (Sec/SPI)",
    "Protein_types": [
      "Signal Peptide (Sec/SPI)",
      "Other"
    ]
  },
  "danRer11_hcXH": {
    "CS_pos": "Cleavage site between pos. 26 and 27: GKA-DV. Probability: 0.9307",
    "Likelihood": [
      0.9761,
      0.0239
    ],
    "Name": "danRer11_hcXH",
    "Plot_eps": "/services/SignalP-5.0/tmp/5E5001DD00004035309DA2C3/output_danRer11_hcXH_plot.eps",
    "Plot_png": "/services/SignalP-5.0/tmp/5E5001DD00004035309DA2C3/output_danRer11_hcXH_plot.png",
    "Plot_txt": "/services/SignalP-5.0/tmp/5E5001DD00004035309DA2C3/output_danRer11_hcXH_pred.txt",
    "Prediction": "Signal peptide (Sec/SPI)",
    "Protein_types": [
      "Signal Peptide (Sec/SPI)",
      "Other"
    ]
  },
  "esoLuc3_hcXA": {
    "CS_pos": "Cleavage site between pos. 22 and 23: IYG-EE. Probability: 0.8242",
    "Likelihood": [
      0.9488,
      0.0512
    ],
    "Name": "esoLuc3_hcXA",
    "Plot_eps": "/services/SignalP-5.0/tmp/5E5001DD00004035309DA2C3/output_esoLuc3_hcXA_plot.eps",
    "Plot_png": "/services/SignalP-5.0/tmp/5E5001DD00004035309DA2C3/output_esoLuc3_hcXA_plot.png",
    "Plot_txt": "/services/SignalP-5.0/tmp/5E5001DD00004035309DA2C3/output_esoLuc3_hcXA_pred.txt",
    "Prediction": "Signal peptide (Sec/SPI)",
    "Protein_types": [
      "Signal Peptide (Sec/SPI)",
      "Other"
    ]
  },
  "esoLuc3_hcXB": {
    "CS_pos": "Cleavage site between pos. 23 and 24: IYG-EE. Probability: 0.6817",
    "Likelihood": [
      0.9404,
      0.0596
    ],
    "Name": "esoLuc3_hcXB",
    "Plot_eps": "/services/SignalP-5.0/tmp/5E5001DD00004035309DA2C3/output_esoLuc3_hcXB_plot.eps",
    "Plot_png": "/services/SignalP-5.0/tmp/5E5001DD00004035309DA2C3/output_esoLuc3_hcXB_plot.png",
    "Plot_txt": "/services/SignalP-5.0/tmp/5E5001DD00004035309DA2C3/output_esoLuc3_hcXB_pred.txt",
    "Prediction": "Signal peptide (Sec/SPI)",
    "Protein_types": [
      "Signal Peptide (Sec/SPI)",
      "Other"
    ]
  },
  "esoLuc3_hcXC": {
    "CS_pos": "Cleavage site between pos. 21 and 22: VHC-DQ. Probability: 0.9646",
```

```
    "Likelihood": [
      0.9937,
      0.0063
    ],
    "Name": "esoLuc3_hcXC",
    "Plot_eps": "/services/SignalP-5.0/tmp/5E5001DD00004035309DA2C3/output_esoLuc3_hcXC_plot.eps",
    "Plot_png": "/services/SignalP-5.0/tmp/5E5001DD00004035309DA2C3/output_esoLuc3_hcXC_plot.png",
    "Plot_txt": "/services/SignalP-5.0/tmp/5E5001DD00004035309DA2C3/output_esoLuc3_hcXC_pred.txt",
    "Prediction": "Signal peptide (Sec/SPI)",
    "Protein_types": [
      "Signal Peptide (Sec/SPI)",
      "Other"
    ]
  },
  "oryLat1_hcXA": {
    "CS_pos": "Cleavage site between pos. 19 and 20: AEA-QL. Probability: 0.9416",
    "Likelihood": [
      0.998,
      0.002
    ],
    "Name": "oryLat1_hcXA",
    "Plot_eps": "/services/SignalP-5.0/tmp/5E5001DD00004035309DA2C3/output_oryLat1_hcXA_plot.eps",
    "Plot_png": "/services/SignalP-5.0/tmp/5E5001DD00004035309DA2C3/output_oryLat1_hcXA_plot.png",
    "Plot_txt": "/services/SignalP-5.0/tmp/5E5001DD00004035309DA2C3/output_oryLat1_hcXA_pred.txt",
    "Prediction": "Signal peptide (Sec/SPI)",
    "Protein_types": [
      "Signal Peptide (Sec/SPI)",
      "Other"
    ]
  },
  "oryLat1_hcXB": {
    "CS_pos": "Cleavage site between pos. 22 and 23: VLS-CR. Probability: 0.9020",
    "Likelihood": [
      0.9983,
      0.0017
    ],
    "Name": "oryLat1_hcXB",
    "Plot_eps": "/services/SignalP-5.0/tmp/5E5001DD00004035309DA2C3/output_oryLat1_hcXB_plot.eps",
    "Plot_png": "/services/SignalP-5.0/tmp/5E5001DD00004035309DA2C3/output_oryLat1_hcXB_plot.png",
    "Plot_txt": "/services/SignalP-5.0/tmp/5E5001DD00004035309DA2C3/output_oryLat1_hcXB_pred.txt",
    "Prediction": "Signal peptide (Sec/SPI)",
    "Protein_types": [
      "Signal Peptide (Sec/SPI)",
      "Other"
    ]
  },
  "salSal2_hcXA": {
    "CS_pos": "Cleavage site between pos. 21 and 22: VHC-DL. Probability: 0.8984",
    "Likelihood": [
      0.9975,
      0.0025
    ],
    "Name": "salSal2_hcXA",
    "Plot_eps": "/services/SignalP-5.0/tmp/5E5001DD00004035309DA2C3/output_salSal2_hcXA_plot.eps",
    "Plot_png": "/services/SignalP-5.0/tmp/5E5001DD00004035309DA2C3/output_salSal2_hcXA_plot.png",
    "Plot_txt": "/services/SignalP-5.0/tmp/5E5001DD00004035309DA2C3/output_salSal2_hcXA_pred.txt",
    "Prediction": "Signal peptide (Sec/SPI)",
    "Protein_types": [
      "Signal Peptide (Sec/SPI)",
      "Other"
    ]
  },
  "salSal2_hcXB": {
    "CS_pos": "Cleavage site between pos. 21 and 22: VHC-DL. Probability: 0.8984",
    "Likelihood": [
      0.9975,
      0.0025
    ],
    "Name": "salSal2_hcXB",
    "Plot_eps": "/services/SignalP-5.0/tmp/5E5001DD00004035309DA2C3/output_salSal2_hcXB_plot.eps",
    "Plot_png": "/services/SignalP-5.0/tmp/5E5001DD00004035309DA2C3/output_salSal2_hcXB_plot.png",
    "Plot_txt": "/services/SignalP-5.0/tmp/5E5001DD00004035309DA2C3/output_salSal2_hcXB_pred.txt",
    "Prediction": "Signal peptide (Sec/SPI)",
    "Protein_types": [
      "Signal Peptide (Sec/SPI)",
      "Other"
    ]
  },
  "salSal2_hcXD": {
    "CS_pos": "Cleavage site between pos. 21 and 22: VHC-DL. Probability: 0.9706",
    "Likelihood": [
      0.9977,
      0.0023
    ],
    "Name": "salSal2_hcXD",
    "Plot_eps": "/services/SignalP-5.0/tmp/5E5001DD00004035309DA2C3/output_salSal2_hcXD_plot.eps",
    "Plot_png": "/services/SignalP-5.0/tmp/5E5001DD00004035309DA2C3/output_salSal2_hcXD_plot.png",
    "Plot_txt": "/services/SignalP-5.0/tmp/5E5001DD00004035309DA2C3/output_salSal2_hcXD_pred.txt",
    "Prediction": "Signal peptide (Sec/SPI)",
    "Protein_types": [
      "Signal Peptide (Sec/SPI)",
      "Other"
    ]
  },
  "salSal2_hcXE": {
    "CS_pos": "Cleavage site between pos. 21 and 22: VHC-DL. Probability: 0.9685",
    "Likelihood": [
      0.9983,
      0.0017
```

```
  ],
  "Name": "salSal2_hcXE",
  "Plot_eps": "/services/SignalP-5.0/tmp/5E5001DD00004035309DA2C3/output_salSal2_hcXE_plot.eps",
  "Plot_png": "/services/SignalP-5.0/tmp/5E5001DD00004035309DA2C3/output_salSal2_hcXE_plot.png",
  "Plot_txt": "/services/SignalP-5.0/tmp/5E5001DD00004035309DA2C3/output_salSal2_hcXE_pred.txt",
  "Prediction": "Signal peptide (Sec/SPI)",
  "Protein_types": [
    "Signal Peptide (Sec/SPI)",
    "Other"
  ]
},
"salSal2_hcXG": {
  "CS_pos": "Cleavage site between pos. 21 and 22: VHC-DL. Probability: 0.9781",
  "Likelihood": [
    0.9974,
    0.0026
  ],
  "Name": "salSal2_hcXG",
  "Plot_eps": "/services/SignalP-5.0/tmp/5E5001DD00004035309DA2C3/output_salSal2_hcXG_plot.eps",
  "Plot_png": "/services/SignalP-5.0/tmp/5E5001DD00004035309DA2C3/output_salSal2_hcXG_plot.png",
  "Plot_txt": "/services/SignalP-5.0/tmp/5E5001DD00004035309DA2C3/output_salSal2_hcXG_pred.txt",
  "Prediction": "Signal peptide (Sec/SPI)",
  "Protein_types": [
    "Signal Peptide (Sec/SPI)",
    "Other"
  ]
},
"salSal2_hcXH": {
  "CS_pos": "Cleavage site between pos. 21 and 22: VHC-DL. Probability: 0.9782",
  "Likelihood": [
    0.9975,
    0.0025
  ],
  "Name": "salSal2_hcXH",
  "Plot_eps": "/services/SignalP-5.0/tmp/5E5001DD00004035309DA2C3/output_salSal2_hcXH_plot.eps",
  "Plot_png": "/services/SignalP-5.0/tmp/5E5001DD00004035309DA2C3/output_salSal2_hcXH_plot.png",
  "Plot_txt": "/services/SignalP-5.0/tmp/5E5001DD00004035309DA2C3/output_salSal2_hcXH_pred.txt",
  "Prediction": "Signal peptide (Sec/SPI)",
  "Protein_types": [
    "Signal Peptide (Sec/SPI)",
    "Other"
  ]
},
"salSal2_hcXI": {
  "CS_pos": "Cleavage site between pos. 21 and 22: VHC-DL. Probability: 0.9719",
  "Likelihood": [
    0.9983,
    0.0017
  ],
  "Name": "salSal2_hcXI",
  "Plot_eps": "/services/SignalP-5.0/tmp/5E5001DD00004035309DA2C3/output_salSal2_hcXI_plot.eps",
  "Plot_png": "/services/SignalP-5.0/tmp/5E5001DD00004035309DA2C3/output_salSal2_hcXI_plot.png",
  "Plot_txt": "/services/SignalP-5.0/tmp/5E5001DD00004035309DA2C3/output_salSal2_hcXI_pred.txt",
  "Prediction": "Signal peptide (Sec/SPI)",
  "Protein_types": [
    "Signal Peptide (Sec/SPI)",
    "Other"
  ]
},
"salSal2_hcXJ": {
  "CS_pos": "Cleavage site between pos. 23 and 24: IYA-AE. Probability: 0.9126",
  "Likelihood": [
    0.9874,
    0.0126
  ],
  "Name": "salSal2_hcXJ",
  "Plot_eps": "/services/SignalP-5.0/tmp/5E5001DD00004035309DA2C3/output_salSal2_hcXJ_plot.eps",
  "Plot_png": "/services/SignalP-5.0/tmp/5E5001DD00004035309DA2C3/output_salSal2_hcXJ_plot.png",
  "Plot_txt": "/services/SignalP-5.0/tmp/5E5001DD00004035309DA2C3/output_salSal2_hcXJ_pred.txt",
  "Prediction": "Signal peptide (Sec/SPI)",
  "Protein_types": [
    "Signal Peptide (Sec/SPI)",
    "Other"
  ]
},
"salSal2_hcXK": {
  "CS_pos": "Cleavage site between pos. 23 and 24: IYA-AE. Probability: 0.9311",
  "Likelihood": [
    0.993,
    0.007
  ],
  "Name": "salSal2_hcXK",
  "Plot_eps": "/services/SignalP-5.0/tmp/5E5001DD00004035309DA2C3/output_salSal2_hcXK_plot.eps",
  "Plot_png": "/services/SignalP-5.0/tmp/5E5001DD00004035309DA2C3/output_salSal2_hcXK_plot.png",
  "Plot_txt": "/services/SignalP-5.0/tmp/5E5001DD00004035309DA2C3/output_salSal2_hcXK_pred.txt",
  "Prediction": "Signal peptide (Sec/SPI)",
  "Protein_types": [
    "Signal Peptide (Sec/SPI)",
    "Other"
  ]
},
"salSal2_hcXL": {
  "CS_pos": "Cleavage site between pos. 23 and 24: IYA-AE. Probability: 0.9274",
  "Likelihood": [
    0.9902,
    0.0098
  ],
  "Name": "salSal2_hcXL",
  "Plot_eps": "/services/SignalP-5.0/tmp/5E5001DD00004035309DA2C3/output_salSal2_hcXL_plot.eps",
```

```
      "Plot_png": "/services/SignalP-5.0/tmp/5E5001DD00004035309DA2C3/output_salSal2_hcXL_plot.png",
      "Plot_txt": "/services/SignalP-5.0/tmp/5E5001DD00004035309DA2C3/output_salSal2_hcXL_pred.txt",
      "Prediction": "Signal peptide (Sec/SPI)",
      "Protein_types": [
        "Signal Peptide (Sec/SPI)",
        "Other"
      ]
    },
    "salSal2_hcXM": {
      "CS_pos": "Cleavage site between pos. 23 and 24: IYA-AE. Probability: 0.9300",
      "Likelihood": [
        0.9907,
        0.0093
      ],
      "Name": "salSal2_hcXM",
      "Plot_eps": "/services/SignalP-5.0/tmp/5E5001DD00004035309DA2C3/output_salSal2_hcXM_plot.eps",
      "Plot_png": "/services/SignalP-5.0/tmp/5E5001DD00004035309DA2C3/output_salSal2_hcXM_plot.png",
      "Plot_txt": "/services/SignalP-5.0/tmp/5E5001DD00004035309DA2C3/output_salSal2_hcXM_pred.txt",
      "Prediction": "Signal peptide (Sec/SPI)",
      "Protein_types": [
        "Signal Peptide (Sec/SPI)",
        "Other"
      ]
    },
    "salSal2_hcXN": {
      "CS_pos": "Cleavage site between pos. 23 and 24: VYA-AE. Probability: 0.9632",
      "Likelihood": [
        0.9964,
        0.0036
      ],
      "Name": "salSal2_hcXN",
      "Plot_eps": "/services/SignalP-5.0/tmp/5E5001DD00004035309DA2C3/output_salSal2_hcXN_plot.eps",
      "Plot_png": "/services/SignalP-5.0/tmp/5E5001DD00004035309DA2C3/output_salSal2_hcXN_plot.png",
      "Plot_txt": "/services/SignalP-5.0/tmp/5E5001DD00004035309DA2C3/output_salSal2_hcXN_pred.txt",
      "Prediction": "Signal peptide (Sec/SPI)",
      "Protein_types": [
        "Signal Peptide (Sec/SPI)",
        "Other"
      ]
    },
    "salSal2_hcXO": {
      "CS_pos": "Cleavage site between pos. 19 and 20: VHG-AN. Probability: 0.9511",
      "Likelihood": [
        0.999,
        0.001
      ],
      "Name": "salSal2_hcXO",
      "Plot_eps": "/services/SignalP-5.0/tmp/5E5001DD00004035309DA2C3/output_salSal2_hcXO_plot.eps",
      "Plot_png": "/services/SignalP-5.0/tmp/5E5001DD00004035309DA2C3/output_salSal2_hcXO_plot.png",
      "Plot_txt": "/services/SignalP-5.0/tmp/5E5001DD00004035309DA2C3/output_salSal2_hcXO_pred.txt",
      "Prediction": "Signal peptide (Sec/SPI)",
      "Protein_types": [
        "Signal Peptide (Sec/SPI)",
        "Other"
      ]
    },
    "salSal2_hcXP": {
      "CS_pos": "Cleavage site between pos. 19 and 20: VHG-AN. Probability: 0.9527",
      "Likelihood": [
        0.999,
        0.001
      ],
      "Name": "salSal2_hcXP",
      "Plot_eps": "/services/SignalP-5.0/tmp/5E5001DD00004035309DA2C3/output_salSal2_hcXP_plot.eps",
      "Plot_png": "/services/SignalP-5.0/tmp/5E5001DD00004035309DA2C3/output_salSal2_hcXP_plot.png",
      "Plot_txt": "/services/SignalP-5.0/tmp/5E5001DD00004035309DA2C3/output_salSal2_hcXP_pred.txt",
      "Prediction": "Signal peptide (Sec/SPI)",
      "Protein_types": [
        "Signal Peptide (Sec/SPI)",
        "Other"
      ]
    }
  },
  "FORMAT": "long",
  "ORG": "Eukarya"
}
```
