# Supplementary material for: Frequent birth-and-death events throughout perforin-1 evolution
Source: BMC Evol Biol. 2020 Oct 19;20:135. doi: 10.1186/s12862-020-01698-1 (PMC7574235; doi:10.1186/s12862-020-01698-1)
Supplement: Supplementary file 12 — Additional file 12 RNA-Seq reads aligned to Rhincodon typusPRF1. Top, Alignment of RNA-Seq reads from whale-shark blood cells to a genomic region belonging to PRF1 and showing premature stop codons. The genomic sequence is shown on a green background. RNA-Seq reads are depicted on a blue (high-quality base) or red (low-quality base) background. Dots and commas represent bases equal to those of the corresponding genomic location. Dashes represent deletions of the reads as compared to the genomic sequence. Bottom, translation of the genomic sequence (green background) and two of the RNA-Seq reads from the top panel. [file 12862_2020_1698_MOESM12_ESM.pdf]

DRX104781 (RNA-seq of whale shark adult blood cells)

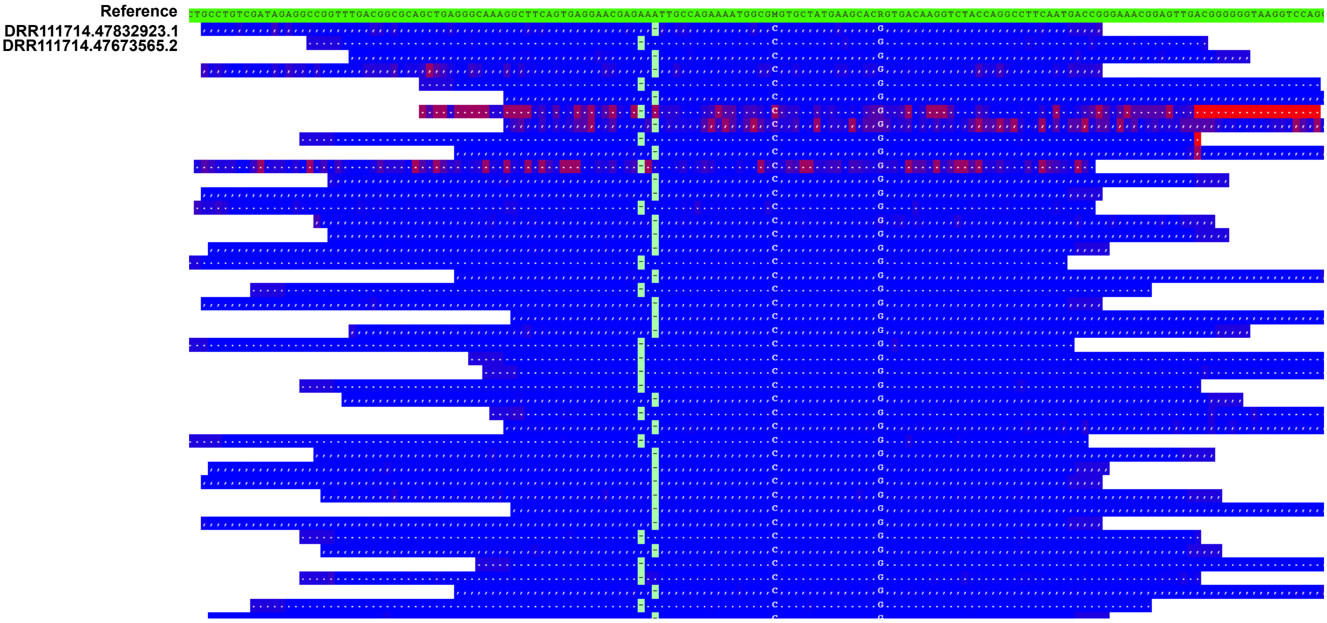

Reference	DCLSIEAGLTAQLRAKASVRNEKLPENGXCYEAX*QGLPGLQ*PGNGVDGG*GPAGGRPF
DRR111714.47832923.1	LSIEAGLTAQLRAKASVRNENCQKMARAMKHGDKVYQAFND
DRR111714.47673565.2	GLTAQLRAKASVRNENCQKMARAMKHGDKVYQAFNDRETEL
